# 1,3-Dideazaguanosine in Atomic Mutagenesis Provides Unprecedented Insight Into Hydrogen Bonding and Stacking Interactions in Folded RNA

**DOI:** 10.1021/jacsau.5c01109

**Published:** 2025-10-31

**Authors:** Marco Oberlechner, Ronald Micura

**Affiliations:** Institute of Organic Chemistry, Center for Molecular Biosciences, Innsbruck (CMBI), 27255 University of Innsbruck, Innrain 80-82, 6020 Innsbruck, Austria

**Keywords:** RNA modifications, deazapurines, nucleoside phosphoramidites, RNA solid-phase synthesis, ribozymes

## Abstract

The central goal of RNA atomic mutagenesis is to evaluate the presumed contacts between individual atoms and their interaction partners with regard to function. This is made possible, for instance, by deaza-modified nucleobases, which are introduced site-specifically into RNA. Mostly, nucleotides with a single nitrogen-to-carbon exchange have been used so far while double exchanges are largely missing although such modification patterns would be highly useful. Here, a systematic study on 1,3-deazaguanosine (c^1^c^3^G) is reported. We present the first synthesis of this nucleoside and an appropriately protected c^1^c^3^G phosphoramidite for RNA solid-phase synthesis. Comprehensive experimentation on c^1^c^3^G modified RNAs, using UV melting profile analysis together with NMR spectroscopy, shed light on the thermodynamics and base pairing properties. We found that c^1^c^3^G destabilizes RNA double helices, but it can integrate well therein without impairing neighboring base pairs. Our data also show that, although two hydrogen bonds are possible in a c^1^c^3^G – C Watson–Crick base pair geometry, the pairing strength is significantly weaker than that of an A–U pair. This can be explained by a loss of stacking capability when the guanine heterocyclic core is replaced by the shape-complementary benzimidazole analog. This observation has implications for the etiology nucleic acids and may explain why purines have evolved as a dominating heterocyclic component of these fundamental biomacromolecules. Furthermore, our findings help to properly apply c^1^c^3^G in atomic mutagenesis experiments, particularly for probing the transition state of self-cleaving nucleolytic RNA. We demonstrate this for the twister ribozyme by identifying a double contact of a guanine in its active site that impacts catalytic activity by 5 orders of magnitude.

## Graphical abstract

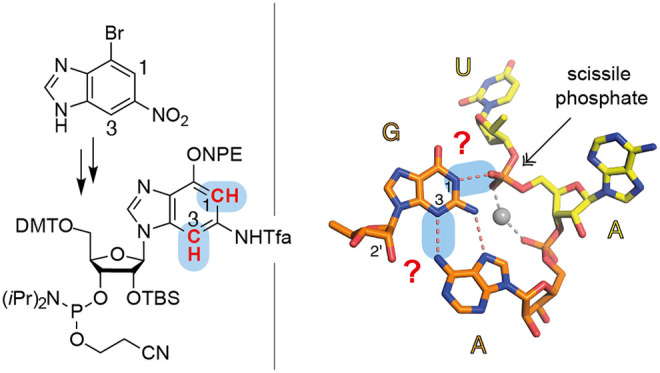

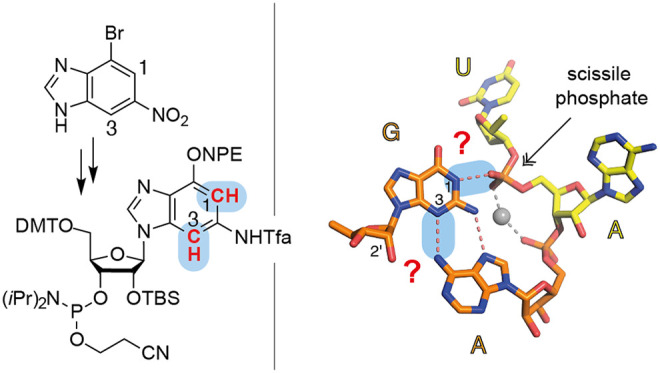

## Introduction

RNA atomic mutagenesis critically assesses the potential interactions between individual atoms (or functional groups) of this RNA and potential intra- or intermolecular interaction partners. To achieve this, deaza-modified nucleotides can be introduced into the target RNA by solid-phase synthesis at the site of interest and the activity of the so-mutated RNA is tested. So far, mostly deaza nucleotides with a *single* nitrogen-to-carbon exchange in their nucleobases have been used to explore RNA structure, function, and reactivity.^1–14^ These include 3-deazacytidine (c^3^C),^14–16^ 7-deazaadenosine (c^7^A),^4,12–14,17,18^ 3-deazaadenosine (c^3^A),^16,19^ 1-deazaadenosine (c^1^A),^12,13,16,19^ 7-deazaguanosine (c^7^G),^14,17^ 3-deazaguanosine (c^3^G),^20,21^ and 1-deazaguanosine (c^1^G).^22,23^ More rarely, atomic mutagenesis based on deaza nucleosides with more than one nitrogen-to-carbon exchange in their nucleobases are found in the literature. One prominent example concerns 1,3-dideaza-adenosine (Figure 1A) that was applied in a structure–function evaluation of two ribozymes (twister^19^ and pistol^18^). For the much larger hatchet ribozyme, this modification also provided valuable insights into structurally and mechanistically relevant adenosine residues, long before the crystal structure became available.^14^ Interestingly, the corresponding 1,3-dideaza guanosine counterpart (Figure 1B) is not yet found in the literature, although in principle this modification would usefully add to the deazanucleoside toolbox for atomic mutagenesis experiments.

**Figure 1:**
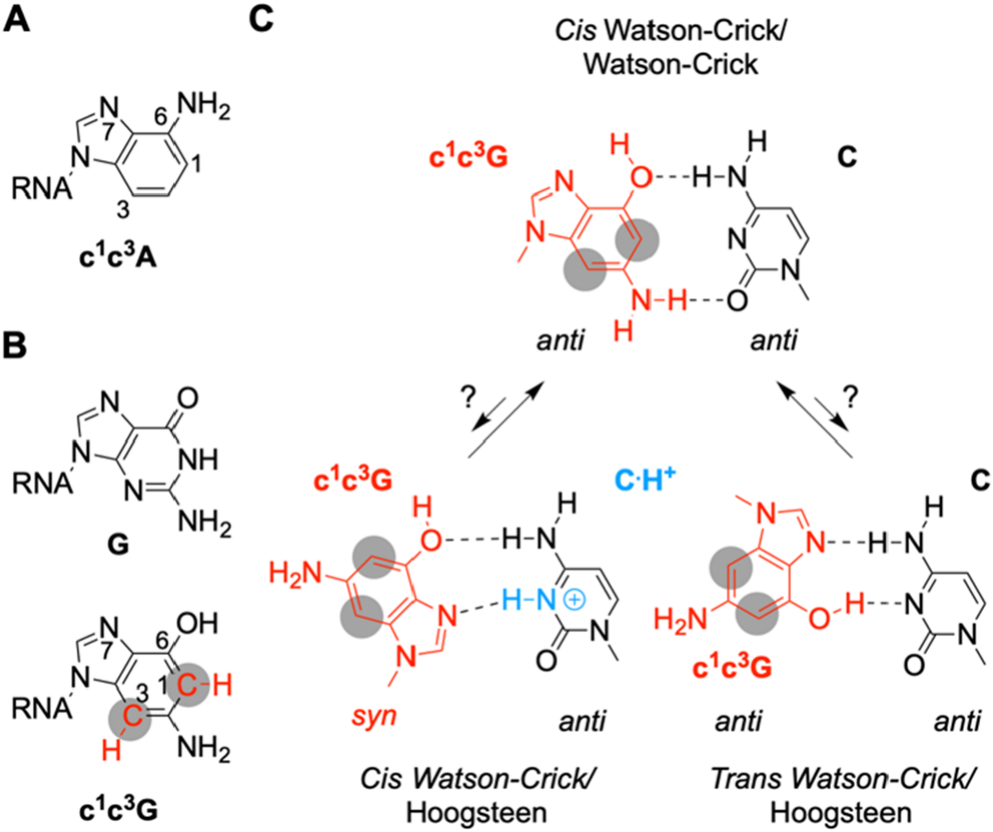
Nucleobase interactions. (A) Chemical structure of 1,3-dideazaadenine in RNA (c^1^c^3^A). (B) Chemical structure of guanosine (G) and 1,3-dideazaguanine (c^1^c^3^G) in RNA. (C) Putative base pairing interactions between c^1^c^3^G and cytidine (c^1^c^3^G -C). Gray shadings indicate deaza positions.

In the present work, we report a robust synthesis of an appropriately protected c^1^c^3^G phosphoramidite building block and its incorporation into oligoribonucleotides by solid-phase synthesis. Furthermore, we describe the impact of c^1^c^3^G on the thermodynamic stability of RNA double helices analyzed by UV spectroscopic melting profile analysis. In addition, using nuclear magnetic resonance (NMR) spectroscopy, we investigated short RNAs to assess pairing interactions in c^1^c^3^G modified double helices (Figure 1C). Finally, c^1^c^3^G was applied in atomic mutagenesis experiments for probing the transition state of a self-cleaving nucleolytic ribozyme (the twister ribozyme), and identified a double contact of a guanine in its active site that is crucial for phosphodiester cleavage.

## Results and Discussion

### Synthesis of 1,3-Deazaguanosine (c^1^c^3^G) Nucleoside

The synthetic route to c^1^c^3^G nucleoside started with bromination of 2,4-dinitroaniline to obtain compound **1** in excellent yields (Scheme 1). Then, regiospecific reduction of the 2-nitro group using octasulfur and sodium sulfide was accomplished, followed by treatment of the crude product with formic acid at elevated temperature to form the imidazo moiety of 2-bromo-6-nitro-1,3-dideazapurine **2**. Next, silyl-Hilbert–Johnson glycosylation of **2** and 1,2,3,5-tetra-*O*-acetyl-β-D-ribofuranose in the presence of *N*,*O*-bis­(trimethylsilyl)-acetamide (BSA) and trimethylsilyl trifluoromethanesulfonate (TMSOTf) provided nucleoside **3**. The exchange of acetyl to *tert*-butyldimethylsilyl (TBS) protection of the ribose hydroxyl groups (compound **4**) was required to enable the installation of the O6-benzyl functionality in a Pd catalyzed cross coupling reaction between benzyl alcohol and the bromo compound **4** by using Buchwald-Hartwig conditions (tBuBrettPhos Pd G3) which gave derivative **5** in very high yields.^24,25^ Finally, selective reduction of the nitro group was conducted by HSiCl_3_ referring to Benaglia and co-workers,^26^ followed by the cleavage of the benzyl group by hydrogenation, and cleavage of the silyl ethers to give 1,3-dideazaguanosine **6** in 28% overall yield in six steps with five chromatographic purifications; in total, 0.35 g of **6** was obtained in the course of this study.

**Scheme 1:**
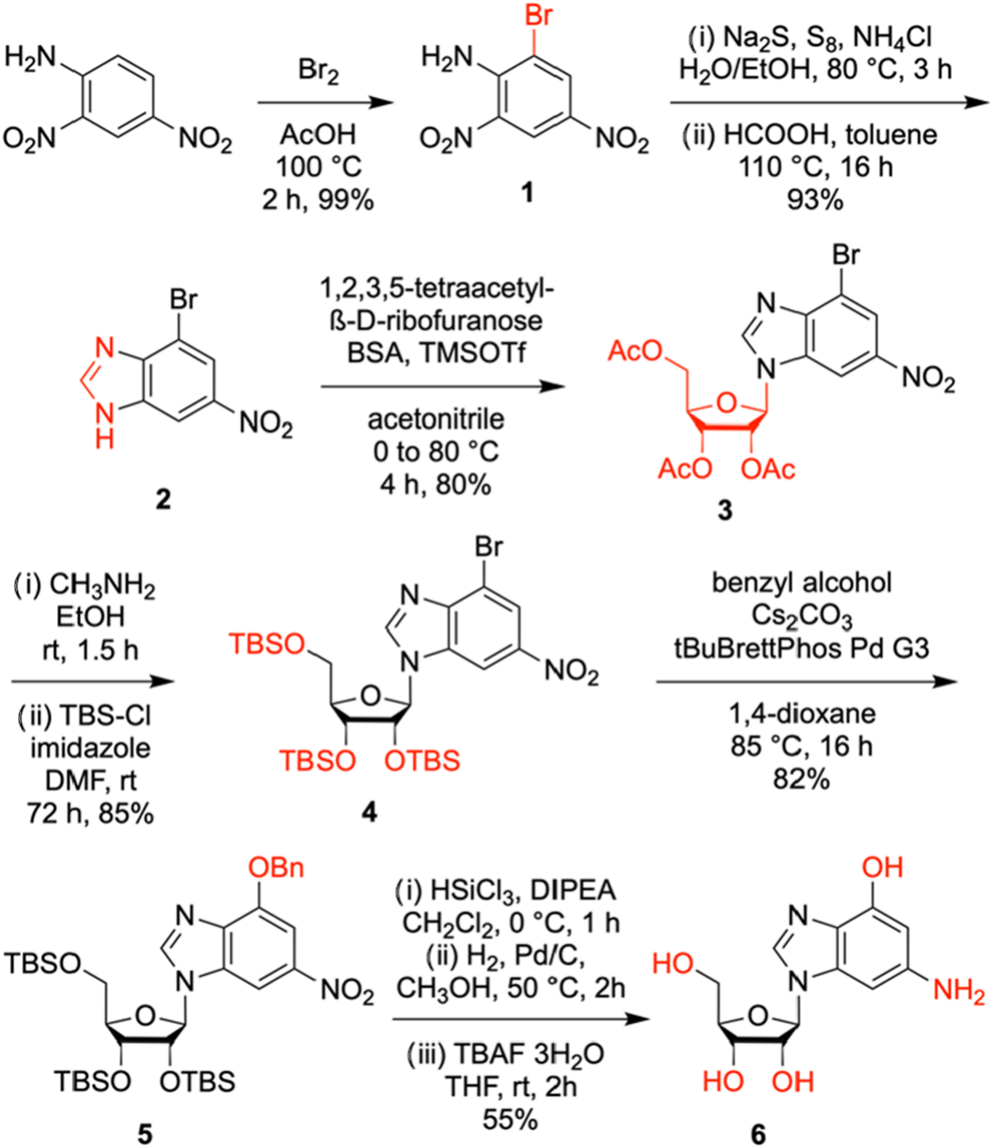
Synthesis of 1,3-Dideazaguanosine (c^1^c^3^G)^a^

a Reaction conditions and yields as indicated. *N*,*O*-Bis­(trimethylsilyl)­acetamide, BSA; trimethylsilyl trifluoromethanesulfonate, TMSOTf; *tert*-butyldimethylsilyl, TBS; *N*,*N*-diisopropylethylamine, DIPEA; tetrabutylammonium fluoride, TBAF.

### Synthesis of c^1^c^3^G Phosphoramidite

The synthetic route to c^1^c^3^G phosphoramidite made use of precursor **5** described in the preceding section (Scheme 1). Selective reduction of its nitro group was again conducted using HSiCl_3_, however, the generated NH_2_ group was then protected using trifluoroacetic anhydride (TFAA) (compound **7**) (Scheme 2). Subsequently, the benzyl protection was removed under hydrogenation conditions to release the hydroxyl group at C6 to furnish nucleoside **8**. Under Mitsunobu conditions using triphenylphosphine, diisopropyl azodicarboxylate (DIAD) and 2-(*p*-nitrophenyl)­ethanol (Npe–OH), the C6-OH of compound **8** was protected with a *p*-nitrophenylethyl (Npe) group, and subsequent deprotection of the *tert*-butyldimethylsilyl (TBS) groups with tetra-*n*-butylammonium fluoride (TBAF) afforded the triol **9**.

**Scheme 2:**
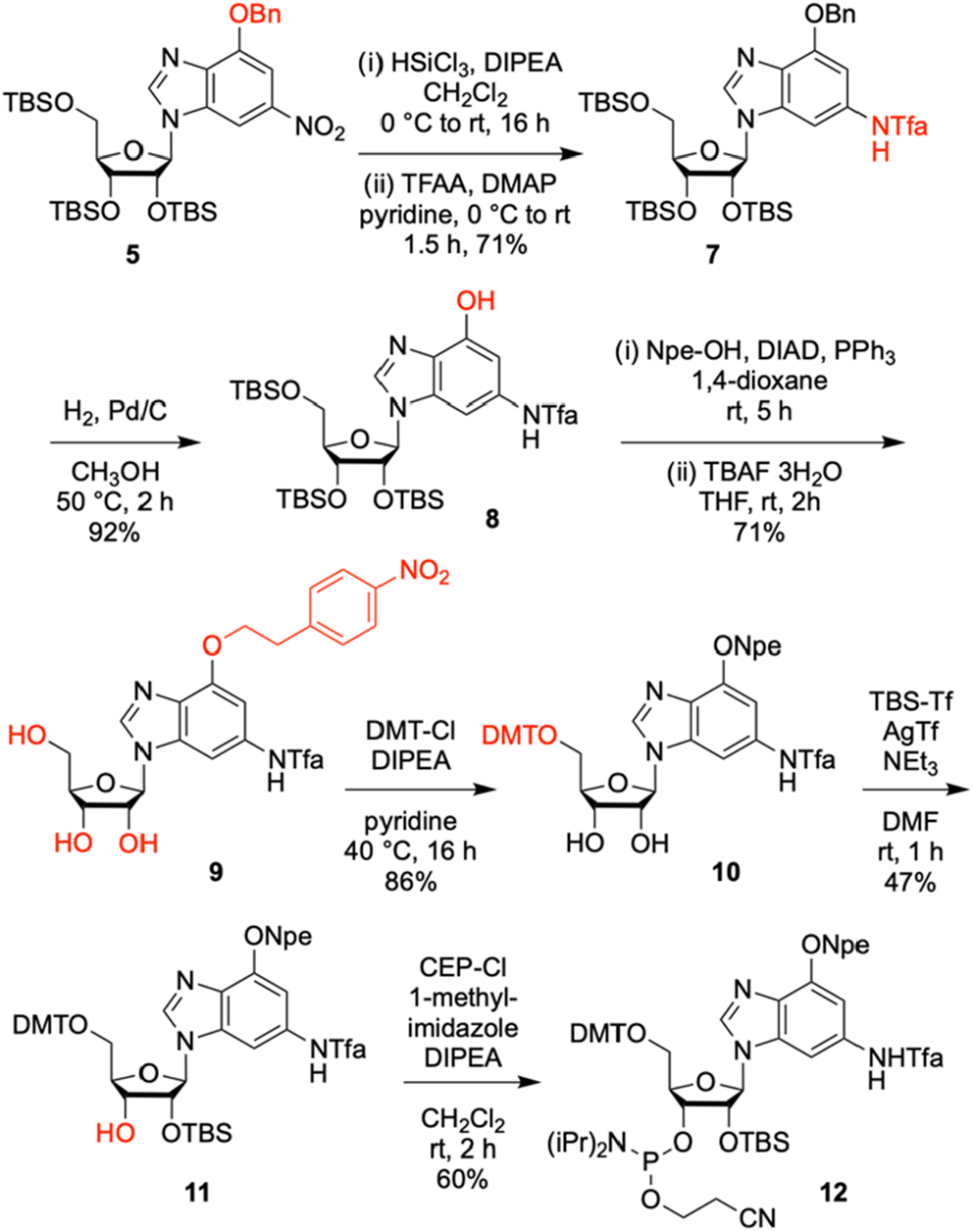
Synthesis of c^1^c^3^G Phosphoramidite^a^

a Reaction conditions and yields as indicated. *tert*-Butyldimethylsilyl, TBS; *N*,*N*-diisopropylethylamine, DIPEA; trifluoroacetic anhydride, TFAA; 4-(dimethylamino)­pyridine, DMAP; trifluoroacetyl, Tfa; 2-(4-nitrophenyl)­ethyl, Npe; diisopropyl azodicarboxylate, DIAD; tetrabutylammonium fluoride, TBAF; 4,4′-dimethoxytrityl, DMT; *tert*-butyldimethylsilyl trifluoromethanesulfonate, TBS-Tf; 2-cyanoethyl-*N*,*N*-diisopropylchlorophosphoramidite, CEP-Cl.

The functionalization of **9** into the desired c^1^c^3^G phosphoramidite **12** required three more transformations. First, nucleoside **9** was converted into the dimethoxytritylated compound **10**, using 4,4′-dimethoxytriphenylmethyl chloride in pyridine. Then, TBS-protection was performed in analogy to Ogilvie^27^ but the procedure was only successful when we replaced TBS-Cl by *tert*-butyldimethylsilyl trifluoromethanesulfonate (TBS-triflate).^28^ The reaction proceeded with high stereoselectivity for silylation of the 3′–OH, therefore base-induced equilibration lead to a 1:1 mixture of 2′ and 3′-O silylated nucleosides that were separated by chromatography, yielding 47% for compound **11**. We note that we did not test the Beigelman 5′,3′-silyl clamp and 2′-O-TBS protection concept^29,30^ which may be an alternative route toward compound **11**. Finally, the phosphoramidite was synthesized under basic conditions using 2-cyanoethyl-*N,N*-diisopropylchlorophosphoramidite (CEP-Cl), 1-methylimidazole and diisopropylethylamine (DIPEA) to give the target compound **12**. Starting from the 6-bromo-2-nitro-1,3-dideazapurine **2**, the c^1^c^3^G-phosphoramidte **12** was synthesized in ten steps, with ten chromatographic purifications and an overall yield of 6%; in total, 0.40 g of **12** was obtained in the course of this study.

### Synthesis of c^1^c^3^G Modified RNA

RNAs with site-specific c^1^c^3^G modifications were synthesized on solid-phase using the new building block **12** together with 2′-O-TBS protected A, C, G U phosphoramidites with *N*-acetyl protection at the nucleobases A, C, and G.^31^ The novel building block **12** was coupled with yields higher than 98% according to the trityl assay. The deprotection of c^1^c^3^G containing RNA needed to be carefully optimized (see also the SI, Supporting Methods, Figures S1, and S2) and starts with 1,8-diazabicyclo[5.4.0]­undec-7-en (DBU) in acetonitrile for 5 h at room temperature to cleave the Npe group under concomitant cleavage of the cyanoethyl groups. Then, acetyl deprotection and the release of the oligonucleotides from the solid support were conducted in a mixture of concentrated ammonia in water and ethanol (3:1) for 3 h at 50 °C. Finally, the 2′-O-silyl protection was cleaved with triethylamine trihydrofluoride (TEA.3HF) in DMSO (1:1). Salts were removed by size-exclusion chromatography, and RNAs were purified by anion-exchange chromatography under denaturating conditions (80 °C column temperature; Figure 2 and Supporting Table S1). The molecular weights of the purified RNAs were confirmed by liquid chromatography (LC) electrospray-ionization (ESI) mass spectrometry (MS). The sequences of c^1^c^3^G containing RNAs synthesized in the course of this study are listed in Supporting Table S1.

**Figure 2:**
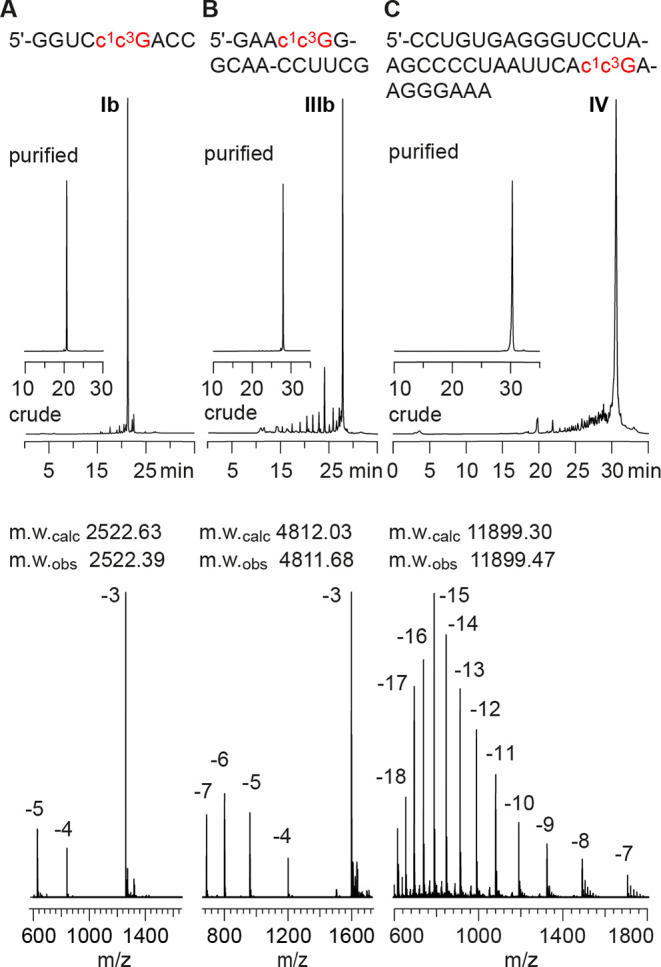
Characterization of c^1^c^3^G-modified RNA using c^1^c^3^G building block 12 for solid-phase synthesis and optimized deprotection conditions. Anion-exchange HPLC traces (top) of purified 9 nt RNA (A), 15 nt RNA (B), and 37 nt RNA (C), and corresponding LC-ESI mass spectra (bottom). HPLC conditions: Dionex DNAPac column (4 × 250 mm), 80 °C, 1 mL min^–1^, 0–60% buffer B in 60 min; buffer A: Tris–HCl (25 mM), 10 mM NaClO_4_, pH 8.0, 20% acetonitrile; buffer B: Tris–HCl (25 mM), 600 mM NaClO_4_, pH 8.0, 20% acetonitrile. For LC-ESI MS conditions, see the Supporting Information.

### Impact of c^1^c^3^G on RNA Base Pairing Stability

In regular RNA double helices, guanosine pairs with cytidine (G-C) in a cis-Watson–Crick geometry. Qualitatively, the strength of a G-C pair is significantly higher than of an adenosine-uridine (A-U) pair, which is attributed to the formation of three instead of only two interstrand hydrogen bonds. The replacement of guanosine by c^1^c^3^G is expected to impair pairing strength because the N1–H of G is replaced by C–H, thereby depriving the capability for the formation of a strong hydrogen bond with N3 of C (Figure 1C). To investigate the thermodynamic impact of the c^1^c^3^G modification we employed different types of RNA double helices, as shown in Figure 3A. The first motif (Type I) represents a bimolecular duplex of nine base pairs with the modification in the center. The second RNA duplex (Type II) forms from a palindromic 10 nt strand, resulting in two sites of modification. This design is very sensitive for the impact arising from a modification on base pairing because only two or three regular Watson–Crick base pairs can form next to the modification and the nucleation of such duplexes is usually substantially hindered.^32,33^ The third RNA motif (Type III) is represented by a hairpin with an extrastable GNRA loop (here GCAA) and the modification residing in the short 5 bp stem.

**Figure 3:**
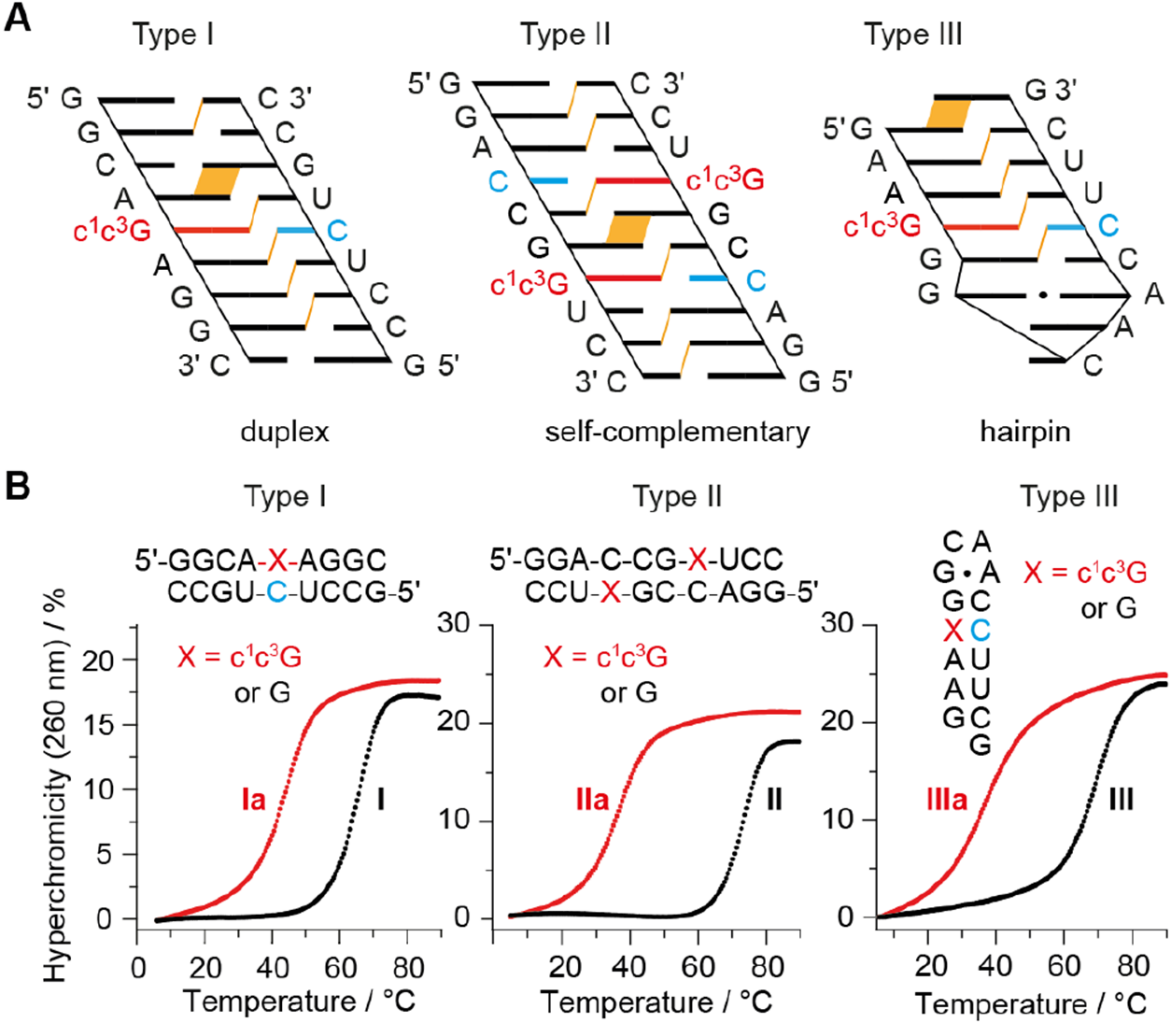
Base pairing stability investigated by UV melting study. (A) Sequence design for thermodynamic analysis of base pairing of c^1^c^3^G modified RNAs. Cartoon presentation to highlight interstrand stacking interactions (in orange). (B) Exemplary UV melting profiles of c^1^c^3^G modified RNAs and unmodified references.

The thermodynamic data we obtained for the three RNA systems by UV-spectroscopic melting measurements are exemplarily illustrated in Figure 3B and summarized in Table 1 (for the corresponding melting profiles, see the Supporting Figures S3 to S8).^34,35^ The *type I* RNA **I** melts at 66.1 °C (Figure 3B). When the central guanosine of the G-C base pair is replaced by c^1^c^3^G, the duplex (**Ia**) is destabilized by 22.0 °C (ΔΔ*G* = 6.2 kcal mol^–1^). The same trend of thermodynamic stabilities for guanosine and 1,3-dideazaguanosine was also found in *type II* palindromic RNA (**II** versus **IIa**; G replaced by c^1^c^3^G results in two c^1^c^3^G-C bp: Δ*T* = −18.7 °C per bp, ΔΔ*G* = 6.0 kcal mol^–1^ per bp) and *type III* hairpin RNA (**III** versus **IIIa**; G replaced by c^1^c^3^G: Δ*T* = −33.8 °C, ΔΔ*G* = 5.5 kcal mol^–1^) investigated here (Figure 3B and Table 1).

**Table 1:** Thermodynamic Parameters of c^1^c^3^G-Modified RNA (and References) Obtained by UV Melting Profile Analysis^a^

#	RNA sequence^a^	*T*_m_ [°C]	Δ*G* ^0^ ^b^ [kcal mol^–1^]	Δ*H* ^0^ ^b^ [kcal mol^–1^]	Δ*S* ^0^ ^b^ [cal mol^–1^ K^–1^]
I	5′GGCA **G** AGGC	66.1	–17.0 ± 0.4	–84.5 ± 1.9	–226 ± 5
	3′CCGUCUCCG				
Ia	5′GGCA **c** ^**1**^ **c** ^**3**^ **G** AGGC	44.1	–10.8 ± 0.3	–68.2 ± 3.6	–193 ± 12
	3′CCGUCUCCG				
ref 36	5′GGCA **A** AGGC	59.5	–15.4 ± 0.1	–84.0 ± 0.7	–230 ± 2
	3′CCGUUUCCG				
II	5′GGACCG **G** UCC	73.4	–21.3 ± 0.1	–104.5 ± 0.6	–279 ± 2
IIa	5′GGACCG **c** ^**1**^ **c** ^**3**^ **G** UCC	36.1	–9.4 ± 0.3	–74.1 ± 2.5	–217 ± 7
ref 36	5′GGAUCG **A** UCC	59.7	–15.8 ± 0.1	–87.0 ± 0.3	–239 ± 1
III	5′GAA **G** G-GCAA-CCUUCG	70.9	–6.9 ± 0.5	–54.1 ± 3.5	–159 ± 10
IIIa	5′GAA **c** ^**1**^ **c** ^**3**^ **G** G-GCAA-CCUUCG	37.1	–1.4 ± 0.1	–32.4 ± 4.7	–105 ± 15
	5′GAA **A** G-GCAA-CUUUCG	57.7	–4.9 ± 0.3	–51.8 ± 3.3	–157 ± 10

a Buffer: 10 mM Na_2_HPO_4_, 150 mM NaCl, pH 7.0. *T*_m_ values are listed at a concentration of 12 μM RNA (calculated from ln c versus 1/T plots). The estimated errors of UV-spectroscopically determined *T*_m_ values are ±0.2 °C. Δ*H* and Δ*S* values were obtained by van’t Hoff analysis according to refs 34 and 35. Errors for Δ*H* and Δ*S*, arising from noninfinite cooperativity of two-state transitions and from the assumption of a temperature-independent enthalpy, are typically 10–15%. Additional error is introduced when free energies are extrapolated far from melting transitions; errors for Δ*G* are typically 3–5%.

b At 298 K. Measurements were performed in three independent experiments. Mean values ± s.e.m. are listed.

Notably, the lower entropic penalty of hybridization of the modified duplexes (especially **IIa** and **IIIa**) could be interpreted as evidence for favorable hydrophobic interaction upon desolvation of the relatively hydrophobic 1,3-dideazaguanine base, as it becomes embedded within the base stack.

In earlier studies, the thermodynamic impact of G replacements in RNA double helices by either 1-deazaguanosine (c^1^G) and 3-deazaguanosine (c^3^G), respectively, were tested.^21,22^ In those studies, the same RNA sequences were used, a direct comparison to the current data set is thus possible. The extent of destabilization caused by 1,3-dideazanucleotide (c^1^c^3^G) is comparable to the sum of the individual contributions from c^1^G and c^3^G (see Supporting Table S2: *type 1* RNA G vs c^1^G), Δ*T* = −15.2 °C, ΔΔ*G* = 3.9 kcal mol^–1^, and G vs c^3^G (Δ*T* = −6.4 °C, ΔΔ*G* = 1.8 kcal mol^–1^). The same context was also found for *type III* hairpin RNA (see Supporting Table S2: G vs c^1^G), Δ*T* = −26.1 °C, ΔΔ*G* = 4.1 kcal mol^–1^, and G vs c^3^G (Δ*T* = −6.7 °C, ΔΔ*G* = 0.7 kcal mol^–1^).

Interestingly, c^1^c^3^G–C containing RNA duplexes are significantly more unstable compared to the corresponding A–U containing RNA, despite both base pairs being able to form two hydrogen bonds (Tables 1 and S2). This suggests that differences in the stacking capabilities are likely responsible for the significant difference in thermodynamic stability.

Notably, for c^1^c^3^G opposite of C, a trans Watson–Crick–Hoogsteen base pair geometry involving a *protonated* C is conceivable as an alternative to the Watson–Crick pairing mode (Figure 1C).^37^ To shed some light on this potential pairing alternative, we conducted pH dependent UV-melting experiments illustrated in Figure 4 (and Supporting Table S3). Strikingly, and in contrast to the unmodified references, for the c^1^c^3^G containing palindrome **II** and hairpin **III**, strong pH dependencies were observed which is consistent with a putative cis Watson–Crick–Hoogsteen base pair geometry involving a *protonated* C (Figure 4C).^37^ We therefore set out to conduct NMR spectroscopy to obtain further evidence for the c^1^c^3^G base pairing mode. These experiments are described below.

**Figure 4:**
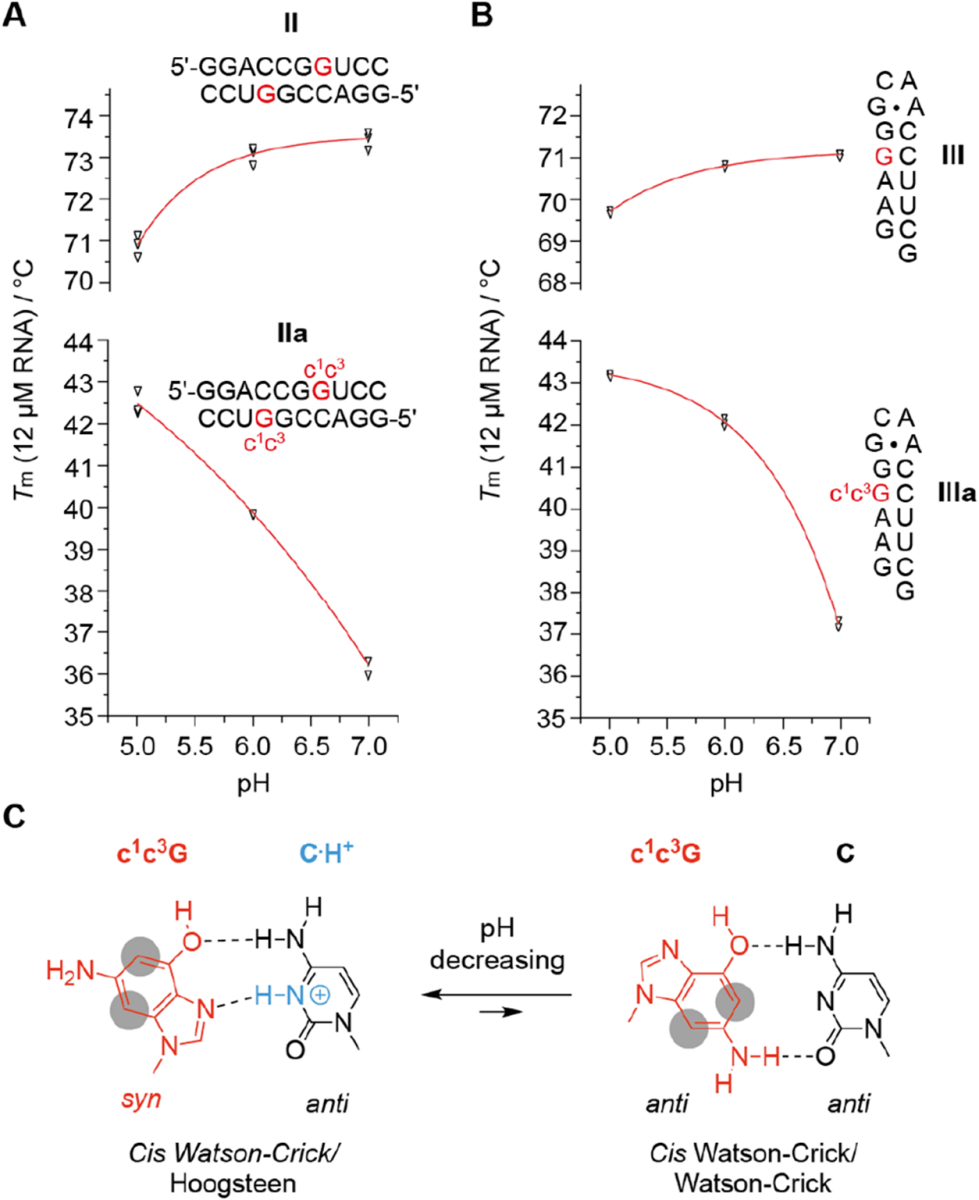
pH dependent UV-melting analysis of c^1^c^3^G modified RNAs. (A) *T*_m_ vs pH plot for the palindromic RNAs II (top) and IIa (bottom). (B) *T*_m_ vs pH plot for the palindromic RNAs III (top) and IIIa (bottom). (C) Putative base pair conformation of c^1^c^3^G modified RNA depending on the pH value of the solution. Conditions: c­(RNA) = 12 μM; 10 mM Na_2_HPO_4_, 150 mM NaCl, pH as indicated. Individual data points (open triangles) (*n* = 3 independent experiments), mean ± s.e.m.

### NMR Spectroscopy of c^1^c^3^G Containing RNA

Watson–Crick base pairs can be readily identified using ^1^H NMR spectroscopy. The signals from hydrogen-bonded protons, known as “imino protons,” provide direct insight into the formation of double helices in folded RNA.^38,39^ The chemical shifts of these signals are distinctive for A-U pairs (approximately 13 to 14.5 ppm) and C-G pairs (approximately 12–13 ppm), while the line widths indicate proton exchange with the surrounding solvent. Additionally, the chemical shifts of imino protons are influenced by chemical modifications, especially those affecting the nucleobase.

Comparative imino proton ^1^H NMR spectra of the hairpin 5′-GAAGG-GCAA-CCUUCG **III** and the c^1^c^3^G–C modified counterpart **IIIa** are depicted in Figure 5. At a temperature of 10 °C, the hairpin **III** displayed five distinct resonances that were assigned to the five Watson Crick base pairs in the stem (Supporting Figure S9),^40^ complemented by a sixth resonance assigned to the G-A loop closing base pair.^41,42^ When G4 was replaced by c^1^c^3^G, one imino proton signal disappeared, consistent with the lack of N1–H in c^1^c^3^G. All other standard Watson–Crick base pairs in the stem retained their imino proton chemical shifts (Figure 5A), indicating that the c^1^c^3^G opposite of C integrates well into an A-form RNA double helix, with little effect on the neighborhood. Of note, when the temperature was raised to 25 °C, the imino proton resonances of the modified hairpin were significantly broadened and lost intensities, indicating pronounced exchange with the solvent (Supporting Figure S10).

**Figure 5:**
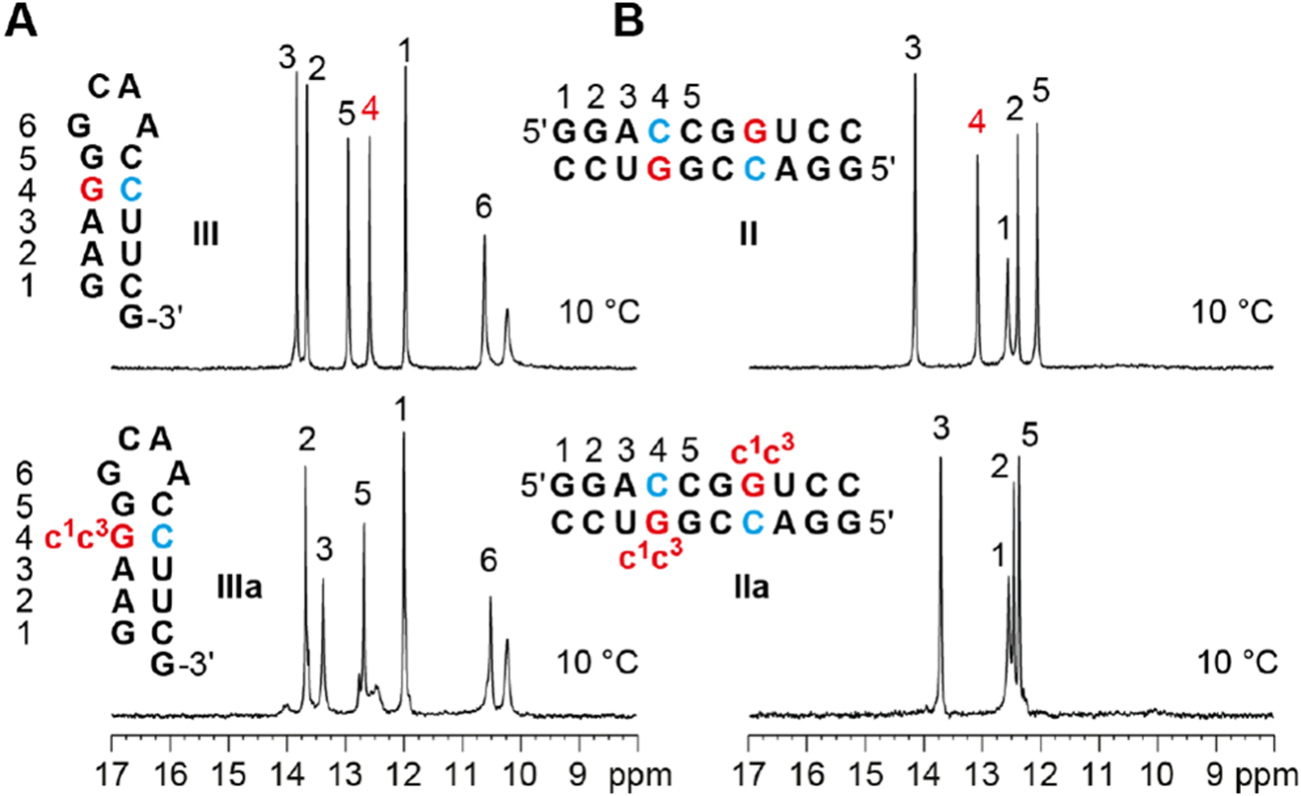
Comparative ^1^H NMR imino proton spectroscopy of unmodified and c^1^c^3^G modified RNA. (A) ^1^H NMR spectra of 15 nt hairpins **III** and **IIIa**, recorded at 10 °C (for measurements at 25 °C see the Supporting Information). Although the c^1^c^3^G modification causes thermodynamic destabilization, the hairpin stem of the c^1^c^3^G hairpin is well formed (**IIIa**); a second minor conformational population is present (duplex competition). (B) ^1^H NMR spectra of 10 nt unmodified (II) and c^1^c^3^G modified palindromes (IIa); the double helix is formed including the direct neighboring base pairs to the c^1^c^3^G–C mismatch. Conditions: c­(RNA): 0.2 mM, 15 mM sodium phosphate, 25 mM NaCl, 10% D_2_O, pH 6.5. All NMR experiments were done at least twice.

Comparative imino proton ^1^H NMR spectra of the palindrome of 5′-GGACCGGUCC **II** and its c^1^c^3^G–C modified counterpart **IIa** are depicted in Figure 5B and reflect essentially the same behavior as observed for the hairpin system. The palindrome **II** displayed five distinct resonances as expected for the five Watson Crick base pairs (Supporting Figure S9).^40^ Upon replacement of G4 by c^1^c^3^G, one signal disappeared (for **IIa**) while the other 4 were clearly visible, and only slightly shifted (Figure 5B).

With the intention to detect a resonance for a putative protonated cytidine in Hoogsteen pairing mode, we lowered the pH to 5.5 (for both RNAs **IIa** and **IIIa**), however, we could not observe a signal (or signal shift) in the range between 14 to 15 ppm that would be expected for such a protonated cytidine.^22^ Likely, proton exchange of the particular c^1^c^3^G-C^+^H pair with the solvent was too fast to be detected with the method used.

### c^1^c^3^G Reveals an Active-Site Guanosine That Causes A More than 10,000-Fold Increase in Ribozyme Activity

Deazanucleobase-modified RNAs are widely used in atomic mutagenesis studies to investigate ribozyme-catalyzed chemical reactions.^10,16,43–48^ These studies have greatly advanced our understanding of general acid–base catalysis in small nucleolytic ribozymes that cleave their phosphodiester backbone. They have highlighted the essential role of imino groups in pyrimidines and purines within the active site. For example, in the twister ribozyme (Figure 6A),^49^ proton transfer from the protonated N3 of a conserved adenine (A6) at the cleavage site plays a significant role in catalysis.^50^ Substituting this adenine with c^3^A or c^1^c^3^A rendered the ribozyme inactive.^19,50^ Similarly, the pistol ribozyme relies on a highly conserved guanine (G33) in the active site.^51^ Substituting this purine with c^7^G drastically reduced activity, and therefore pointed at a crucial contact observed in the crystal structure, namely an inner-sphere coordination to Mg^2+^ ion to N7 of G33.^17,18^ The substitution with c^7^G impairs positioning of a Mg^2+^ first-shell water molecule that acts as a general acid to protonate the 5′-O leaving group in the reaction course.

**Figure 6:**
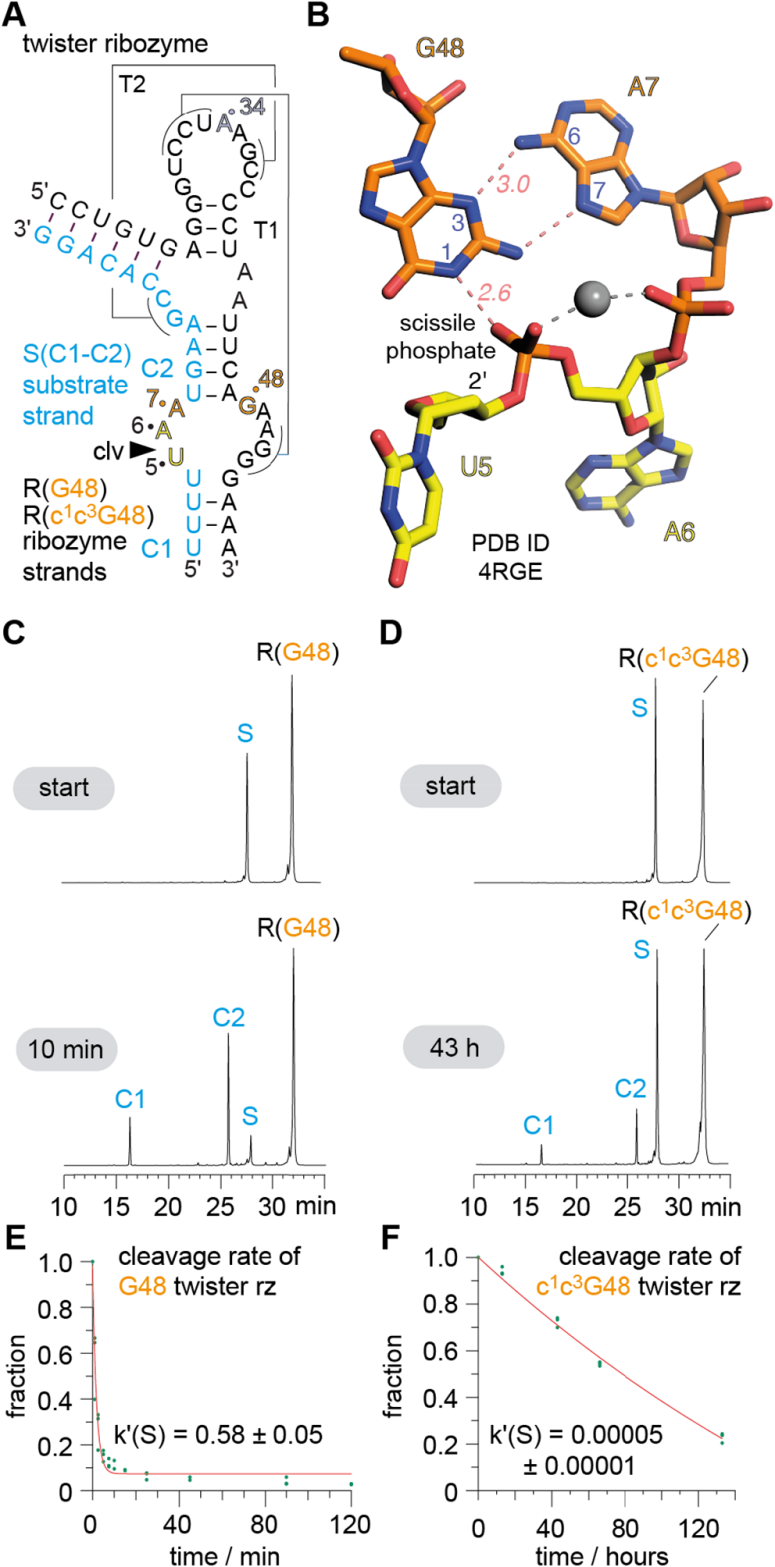
Atomic mutagenesis of the twister ribozyme: active site G48-to-c^1^c^3^G mutation impedes cleavage and highlights crucial interactions between G48 and the scissile phosphodiester. (A) Secondary structure of the two-strand ribozyme assembly used for functional cleavage assays. (B) Crystal structure of the twister ribozyme in a precatalytic state (PDB ID 4RGE).^52^ Active site with G48 interacting with A7 (orange) and the to-be-cleaved phosphate between dU5-A6 (yellow); distances (italics style) in Å. (C) HPLC traces of wild-type G48 (left) and c^1^c^3^G48 modified (D) ribozyme at two time points illustrate that product formation of the c^1^c^3^G-modified ribozyme is significantly impeded under otherwise same reaction conditions. Cleavage rate determination of wild-type G48 (E) and c^1^c^3^G48 (F) ribozymes. Individual data points (green circles) (*n* = 2 (for E), and *n* = 3 (for F) independent experiments), mean ± s.e.m.

The new c^1^c^3^G modification now makes it possible to investigate whether specific *double* contacts of active-site guanines contribute to RNA catalysis. On such putative double contact (which actually has been the driving force to develop the c^1^c^3^G modification) is an active site guanine in the twister ribozyme.^50^ Several precatalytic crystal structures have been described for twister.^19,52–58^ At the cleavage site U5-A6, a guanine (G48 in PDB structures 4RGE and 5DUN)^19,52^ is located within hydrogen-bonding distance (2.6 Å) between its N1 atom and the nonbridging pro-R oxygen of the scissile phosphate. Simultaneously, the N3 atom of this G48 is involved in trans Hoogsteen/sugar edge base pair formation with an adenine (A7) (Figure 6B). In principle, G48 could function as a general base in phosphodiester cleavage,^59,60^ either by N1 deprotonation, or by enol tautomerization, or at the very least, by hydrogen bonding to activate the attacking 2′–OH nucleophile (β-catalysis).^59,60^ G48 may additionally contribute to stabilizing the pentavalent phosphorane transition state (γ-catalysis),^59,60^ as suggested by the G48N1–H···O = P interaction observed in crystal structures (Figure 6B).^19,50,52^

Replacing G48 with c^1^c^3^G should significantly reduce the specific N1 and N3 hydrogen bonding interactions observed in the crystal structure. Our expectation that this would impair ribozyme activity was confirmed by cleavage experiments using the c^1^c^3^G48 modified twister variant. The modified ribozyme showed drastically reduced phosphodiester cleavage, at a rate 11,600 times slower than the unmodified ribozyme (Figures 6C–F, S11 and S12). When we mutated G48 to c^1^G, which retains the ability to form an A7-G48 pair via its N3 imino group, the cleavage rate was only 275 times slower.^22^ Together, these findings support the idea that the proper positioning of G48, enabled by its interaction with the Hoogsteen face of A7, is crucial for catalysis. In this context we also note that the A7–G48 base pair stacks onto the U8–A47 pair, thereby extending the central stem, and additionally G48 stacks on U4.^52^ Therefore, the drastically reduced cleavage rate observed for the c^1^c^3^G48 mutant is not only a result of a loss of hydrogen bonding but additionally, the stacking arrangement (G48 sandwiched between A47 and U4) seems perturbed.

The minimal residual activity of c^1^c^3^G modified twister suggests that the other catalytic strategies (α- and δ-catalysis)^59,60^ are only sufficient to maintain a very minor level of function. It is likely that the guanine exocyclic 2-NH_2_ group, which is retained in c^1^c^3^G (and in c^1^G) still contributes to phosphorane stabilization, along with a weak, stabilizing effect from a C1–H interaction.

Of note, a recent review lists six out of eight known nucleolytic ribozymes that contain a conserved G in the active site.^36^ This G plays the role of a base in a general acid–base mechanistic scenario that explains phosphodiester cleavage. These ribozymes are all suitable candidates for functional analysis with c^1^c^3^G mutants, as well as c^1^G and c^3^G.^21–23^ Such experiments allow us to evaluate the general acid–base mechanism proposal and deepen our mechanistic understanding of RNA catalysis. They may also provide a clearer explanation for why G is often found in these ribozyme positions.

## Conclusions

Our research presents robust methods for synthesizing c^1^c^3^G, its corresponding phosphoramidite, and c^1^c^3^G-modified RNA. This synthetic framework facilitated a thorough investigation of the biophysical properties of c^1^c^3^G-modified RNA. Our studies provided evidence that c^1^c^3^G has a lower propensity for stacking than native G, which is likely responsible for the lower observed pairing strength when c^1^c^3^G is placed in a regular double helix. Furthermore, c^1^c^3^G tends to switch from Watson–Crick to Hoogsteen pairing mode which involves a protonated cytosine. Importantly, c^1^c^3^G atomic mutagenesis can be applied to functional RNA assays to directly test ribozyme mechanistic hypotheses that propose a functional role for guanosines through their N1 and N3 positions. For the twister ribozymes, we found that a conserved guanine in the active site can only catalyze via the N1 position (i.e., activating the attacking 2′–OH (β-catalysis) and stabilizing the phosphorane transition state (γ-catalysis)) when properly positioned by forming an intact Hoogsteen base pair with its N3 face. Catalytic guanosines are also present in many other ribozymes.^10,61^ Using the double dissecting c^1^c^3^G modification will advance our understanding of RNA catalysis in ribozymes and is therefore an invaluable addition to the RNA functional atom mutagenesis toolbox.

## Supplementary Material



## Notes

Additional methods information, synthetic procedures and NMR spectra of all compounds, table of all RNAs synthesized and MS data, RNA melting curves, NMR spectra, and HPLC cleavage assays of the twister ribozyme (PDF)
