# Supplementary material for: 1,3-Dideazaguanosine in Atomic Mutagenesis Provides Unprecedented Insight Into Hydrogen Bonding and Stacking Interactions in Folded RNA
Source: JACS Au. 2025 Oct 31;5(11):5626–34. doi: 10.1021/jacsau.5c01109 (PMC12648317; doi:10.1021/jacsau.5c01109)
Supplement: Supplementary file 1 [file au5c01109_si_001.pdf]

Supporting Information

1,3-Dideazaguanosine in atomic mutagenesis provides unprecedented insight into hydrogen bonding and stacking interactions in folded RNA

*Marco Oberlechner and Ronald Micura**

Institute of Organic Chemistry, Center for Molecular Biosciences, Innsbruck (CMBI), University of Innsbruck, Innrain 80-82, 6020 Innsbruck, Austria

Contents

Supporting Methods

Materials and general procedures S02
Synthesis of 1,3-dideazaguanosine **6** S05
Synthesis of 1,3-deazaguanosine phosphoramidite **12** S20

Supporting Tables

Supporting Table S1 S36
Supporting Table S2 S37
Supporting Table S3 S38

Supporting Figures

Supporting Figure S1 S39
Supporting Figure S2 S40
Supporting Figure S3 S41
Supporting Figure S4 S42
Supporting Figure S5 S43
Supporting Figure S6 S44
Supporting Figure S7 S45
Supporting Figure S8 S46
Supporting Figure S9 S47
Supporting Figure S10 S48
Supporting Figure S11 S49
Supporting Figure S12 S50

Supporting Methods

Materials and general procedures

Materials

Reagents were purchased in the highest available quality from commercial suppliers (Merck / Sigma-Aldrich, ABCR, VWR, ChemGenes, CarboSynth, Synthonix) and used without further purification. All reactions were carried out under argon atmosphere, unless otherwise noted. Analytical thin-layer chromatography (TLC) was performed on Macherey-Nagel Polygram® SIL G/UV254 plates. 0.2 mm Silica gel 60 for column chromatography was purchased from Macherey-Nagel.

NMR measurements of compounds

^{1}H, ^{13}C, and ^{31}P spectra were recorded on a Bruker Ultrashield™ 400 Plus spectrometer. Chemical shifts (δ) are reported relative to tetramethylsilane (TMS), referenced to the residual solvent signal (DMSO-d_6: 2.50 ppm for ^{1}H and 39.52 ppm for ^{13}C spectra; CDC_{l3}: 7.26 ppm for ^{1}H and 77.16 ppm for ^{13}C spectra). The following abbreviations were used to denote multiplicities: s = singlet, d = doublet, t = triplet, q = quadruplet, m = multiplet, b = broad. Signal assignments are based on ^{1}H-^{1}H-COSY, ^{1}H-^{13}C-HSQC and ^{1}H-^{13}C-HMBC experiments.

High-resolution mass spectrometry of compounds

High resolution mass spectra were recorded in positive ion mode on a Thermo Scientific Q Exactive Orbitrap, ionized via electrospray at 3.7 kV spray voltage.

RNA solid-phase synthesis

Standard phosphoramidite chemistry was applied for RNA strand elongation and incorporation of 1,3-dideazaguanosine. 2'-*O*-TBDMS & N-acetyl protected nucleoside phosphoramidite building blocks and 2'-*O*-TBDMS 1000 Å CPG solid support (>15nt) were purchased from ChemGenes, Primer support™ 5G (<15nt) was purchased from GE Healthcare. All oligonucleotides were synthesized on a ABI 392 Nucleic Acid Synthesizer following standard methods: detritylation (100 sec) with dichloroacetic acid/1,2-dichloroethane (4/96); coupling (7.0 min) with phosphoramidites/acetonitrile (100 mM, 200 µL) and benzylthiotetrazole / acetonitrile (300 mM, 500 µL); capping (2 x 25 sec) with Cap A mild / Cap B mild (1/1 v/v), Cap A mild: phenoxyacetic anhydride/acetonitrile (100 mM), Cap B mild: *N*-methylimidazole / *sym*-collidine / tetrahydrofuran (0.160/0.265/10 v/v/v); oxidation (30 sec) with iodine (20 mM) in tetrahydrofuran/pyridine/H_2O (35/10/5 v/v/v). Phosphoramidites were diluted to a concentration of 0.1 mol/l, 1,3-dideazaguanosine to 0.07 mol/l and dried over activated molecular sieves (3 Å) overnight.

Deprotection, purification and quantification of RNA

c^1c^3G modified RNA. For basic deprotection of the modified c^1c^3G - RNA, the solid support was pretreated with a 1M DBU in acetonitrile solution for 7 hours at 25 °C (fresh 1M DBU every 30 min) to remove cyanoethyl and 4-nitrophenylethyl NPE moieties. The solid support was then washed with acetonitrile (15 mL) three times each, dried under high vacuum and deprotected with 3:1 aqueous ammonia (28 %, 0.90 mL) in ethanol (0.30 mL) for 3 hours at 50 °C. The supernatant was removed and the solid support was washed twice with 0.5 mL tetrahydrofuran/H_2O (1/1). Combined

supernatant and washings were evaporated to dryness and the residue was dissolved in a 1:1 solution of triethylamine trishydrofluoride (0.3 mL) in dimethyl sulfoxide (0.3 mL) and incubated for 14 hours at 37 °C, for the removal of 2'-*O*-silyl protecting groups. The reaction was quenched by the addition of GLEN research quenching Buffer for RNA (water/TRIS base/Tris HCl (7:2:1), pH = 7-8) and the sample was desalted. Desalting was performed via size-exclusion column chromatography (GE Healthcare, HiPrep^{TM} 26/10 Desalting; Sephadex G25) eluting with H_2O; collected fractions were evaporated and the RNA dissolved in H_2O (0.6 mL). The crude RNA was purified by anion exchange chromatography on a GE Healthcare Äkta Explorer HPLC System containing a semipreparative Dionex DNAPac^{TM} PA-100 column (9 mm x 250 mm) 80 °C, a pressure between 4.0 and 4.6 MPa and a flow rate of 2 mL/min (Eluent A: 25 mM Tris·HCl, 0.01 M $NaClO_4$, 20% acetonitrile, pH 8.0; Eluent B: 25 mM Tris·HCl, 0.6 M NaClO4, 20 % acetonitrile, pH 8.0). Fractions containing RNA were diluted with 0.1 M triethylammonium bicarbonate solution, loaded on a C18 SepPak Plus® cartridge (Waters/Millipore), washed with H_2O and eluted with acetonitrile/H_2O (1/1). Crude and purified RNA were analyzed by anion exchange chromatography on a GE Healthcare äkta explorer HPLC System containing a Dionex DNAPac^{TM} PA-100 column (4 mm x 250 mm) at 80 °C with a flow rate of 1 mL/min and a gradient of 0 – 60 % B in 60 minutes was used; Eluent A: 25 mM Tris·HCl, 0.01 M $NaClO_4$, 20 % acetonitrile, pH 8.0; Eluent B: 25 mM Tris·HCl, 0.6 M $NaClO_4$, 20 % acetonitrile, pH 8.0 HPLC traces were recorded at UV absorption by 260 nm. RNA quantification was performed on an Implen P300 Nanophotometer.

Unmodified RNA. For basic deprotection of unmodified RNA, the solid support was mixed with aqueous methylamine (40 %, 0.65 mL) and aqueous ammonia (28 %, 0.65 mL) for 15 minutes at 65 °C. The supernatant was removed and the solid support was washed twice with 0.5 mL tetrahydrofuran/H_2O (1/1). Combined supernatant and washings were evaporated to dryness and the residue was dissolved in a solution of tetra-*n*-butylammonium-fluoride in tetrahydrofuran (1.0 M, 1.5 mL) and incubated for 14 hours at 37 °C, for the removal of 2'-*O*-silyl protecting groups. The reaction was quenched by the addition of aqueous tetraethylammonium acetate solution (1.0 M, 1.5 mL, pH 7.4). Tetrahydrofuran was removed under reduced pressure and the sample was desalted, purified and quantified equally to the c^1c^3G modification procedure above.

Mass spectrometry of unmodified and modified RNA

RNA samples (3 µL) were diluted with 40 mM Na_2H_2(EDTA)/H_2O (5/4) for a total volume of 30 µL, injected onto a C18 XBridge 2.5 µm (2.1 mm x 50 mm) column at a flow rate of 0.1 mL/min and eluted with 0 – 100 % B gradient at 30 °C (Eluent A: 8.6 mM triethylamine, 100 mM 1,1,1,3,3,3-hexafluoroisopropanol in H_2O; Eluent B: methanol). RNA traces were analyzed on a Finnigan LCQ Advantage Max electrospray ionization mass spectrometer with 4.0 kV spray voltage in negative mode.

Melting Curve measurements of unmodified and modified RNA

RNA samples were lyophilized as triethylammonium salts, dissolved in 800 or 330 µL Buffer (10 mM Na_2HPO_4 containing 150 mM NaCl at pH 7.0) and transferred into UV permeable high precision cells made of quartz SUPRASIL® with a light path of 10 mm or 1 mm. UV melting profiles were recorded at 250 and 260 nm on Varian Cary 100 or Agilent Cary 3500 UV-Vis spectrophotometers equipped with multiple cell holders and peltier temperature control devices. Each RNA was measured at five different concentrations (between ~1 and ~100 µM) and with at least four ramps (heating-cooling-

heating-cooling; 1°C min^{-1} heating/cooling rate). T_m values were determined by calculating the first derivative, usually from data of the third ramp (heating).

RNA NMR experiments

RNA samples were lyophilized as sodium salts, dissolved in 430 µL NMR buffer (15 mM NaP, 25 NaCl, 10 % D_2O, pH 6.5) and transferred into 5 mm NMR tubes. A sample concentration of 0.7 mM was used. All NMR experiments were conducted on a Bruker 600 MHz Avance II+ NMR or a 700 MHz Avance Neo NMR both equipped with a Prodigy TCI probe.

Ribozyme cleavage assays

The ribozyme and substrate strands (2.42 nmol each) were lyophilized as triethylammonium salts and dissolved in 33 µL H_2O. The dissolved RNA strands were heated to 90 °C for 2 minutes and subsequently cooled to room temperature. 2 µL of this solution were used for time point zero. The remaining was mixed with 6 µL of 200 mM HEPES pH 7.5, 2 µL KCl 2 M and 2 µL $MgCl_2$ 40 mM to reach a final concentration of 55 µM of each RNA strand, 30 mM HEPES, 100 mM KCl and 2 mM Mg^{2+}. Cleavage reaction was initiated by the addition of $MgCl_2$ solution. Samples (3 x 2 µL each) were drawn after the indicated time points and quenched by the addition of equal amounts of 40 mM Na_2H_2EDTA and diluted to 100 µl. For statistical reasons, each time point was measured three times, therefore the procedure has to be adapted in terms of RNA quantity, water, salt and buffer addition between wildtype and mutant. We assayed the ribozyme under single turnover conditions for reasons of comparison to our previous studies on the twister ribozyme.

Synthesis of 1,3-dideazaguanosine 6

2-Bromo-4,6-dinitroaniline (1)

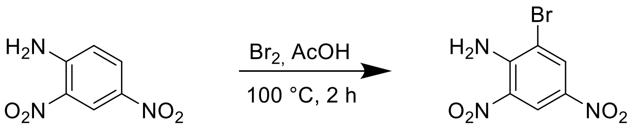

1

Acetic acid (40 mL) was added to 2,4-dinitroaniline (2.2 g, 12.0 mmol) followed by dropwise addition of bromine (2.88 g, 1.0 mL, 4.09 mol) over 20 min at room temperature. The reaction was then heated to 100 °C for 2 h. Subsequently, the reaction mixture was cooled to room temperature and poured onto ice-cold water (40 mL). The suspension was adjusted to basic pH (pH 8-10) and the solid was filtered off, washed with ice cold water and n-pentane. The resulting solid was dried under high vacuum to give compound **1**.

Yield: 1.42 g of compound **1** as a yellow solid (quantitative)
TLC (dichloromethane/methanol, 95/5)**:** R_f = 0.78
HR-ESI-MS (m/z): $[M+H]^+$ calcd. 262.9359; found 262.9323

^{1}H-NMR (400 MHz, DMSO-d_6, 25 °C):
δ = 8.13 (2H, s*,* ***H₂****N*-C(1)); 8.59 (1H, d, J=2.62 Hz, ***H***-C(5)); 8.82 (1H, d, J= 2.68 Hz, ***H***-C(3)) ppm.

^{13}C-NMR (100 MHz, DMSO-d_6, 25 °C):
δ = 111.66 (***C***(6)); 123.12 (***C***(3)); 130.55 (***C***(2)); 133.00 (***C***(5)); 135.35 (***C***(1)); 147.14 (***C***(4)) ppm.

^{1}H-NMR (400 MHz, DMSO-d_6, 25 °C):

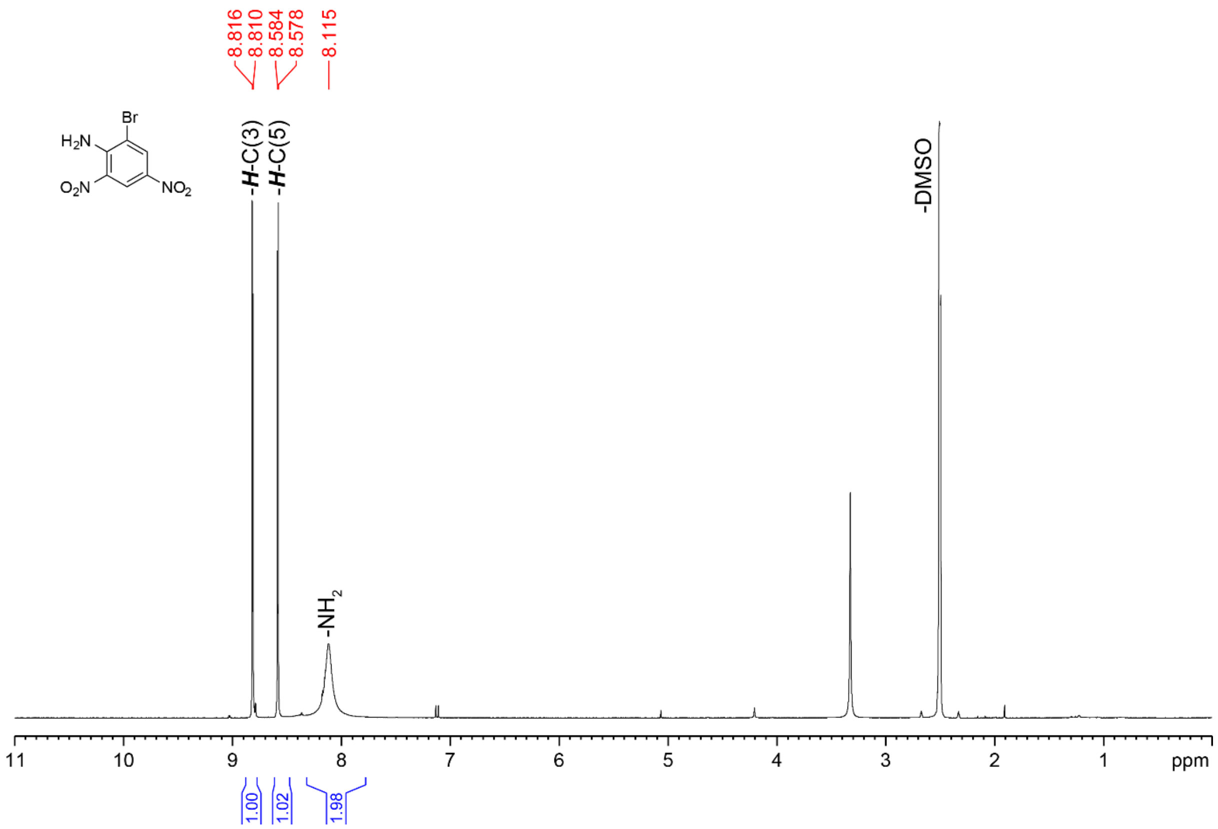

^{13}C-NMR (100 MHz, DMSO-d_6, 25 °C):

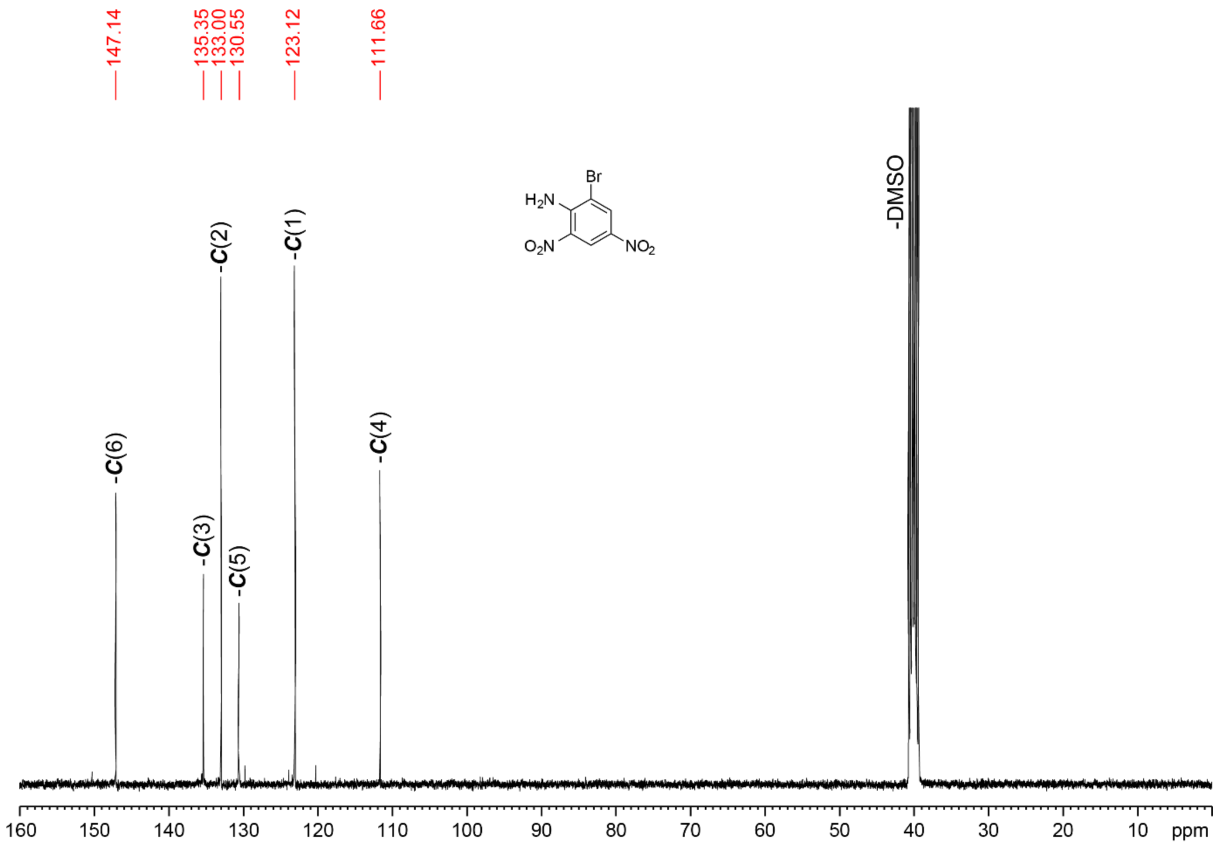

6-Bromo-2-nitropurine (2)

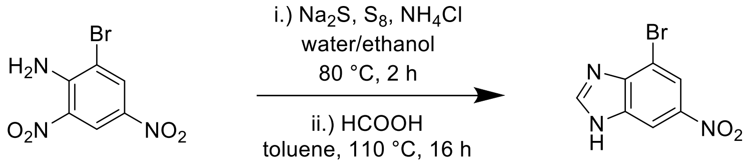

1 2

Step i.) Water (40 mL) and ethanol (10 mL) were added to a flask charged with sodium sulfide (938 mg, 12.0 mmol) and sulfur (385 mg, 12.0 mmol). The mixture was refluxed at 80 °C for 1 h. The solution was then added to a flask containing 2-bromo-4,6-dinitroaniline **1** (3.15 g, 12.0 mmol) and ammonium chloride (964 mg, 18.0 mmol) in water (40 mL) and ethanol (70 mL). The reaction mixture was stirred at 80 °C for 30 min. Subsequently, a solution of 2N NaOH (40 mL) was added dropwise to the reaction mixture, which was further stirred at 80 °C for 30 min. The resulting dark solution was allowed to rest to room temperature, poured into ice water, neutralized to pH 7 with a 2 N HCl solution and extracted three times with ethyl acetate. The combined organic layers were washed with brine, dried over sodium sulfate, filtered, and concentrated under reduced pressure to afford the crude title product as a rust colored solid.
Step ii.) Formic acid (2.46 g, 2.02 mL, 53 mmol) was added to 3-bromo-5-nitrobenzene-1,2-diamine (3.10 g, 13.4 mmol) in toluene (10 mL). The solution was heated to reflux overnight. The product precipitated and was triturated with toluene, diluted with water and adjusted to pH 9 with 6N NaOH. The solid was filtered, washed with water and placed in a vacuum oven overnight to yield compound **2** as a brown solid. For the c^{13}-NMR sample, the purine derivative was then dissolved in 3 M HCl and the excess water is removed by evaporation and dried under high vacuum.

Yield: 3.18 g of compound **2** as a brown solid (96%)
TLC (dichloromethane/methanol, 95/5)**:** R_f = 0.50
HR-ESI-MS (m/z): $[M+H]^+$ calcd. 241.9515; found 241.9558

^{1}H-NMR (400 MHz, DMSO-d$_6$, 25 °C):
δ = 8.26 (1H, d, J=2.59 Hz, ***H***-C(1)); 7.63 (1H, d, J=2.59 Hz, ***H***-C(3)); 8.65 (1H, s, ***H***-C(8)) ppm.

^{13}C-NMR (100 MHz, DMSO-d$_6$, 25 °C):
δ = 109.36 (***C***(6)); 110.94 (***C***(3)); 120.87 (***C***(1)); 135.16 (***C***(4)); 142.07 (***C***(5)); 143.97 (***C***(2)); 143.97 (***C***(8)) ppm.

^{1}H-NMR (400 MHz, DMSO-d$_6$, 25 °C):

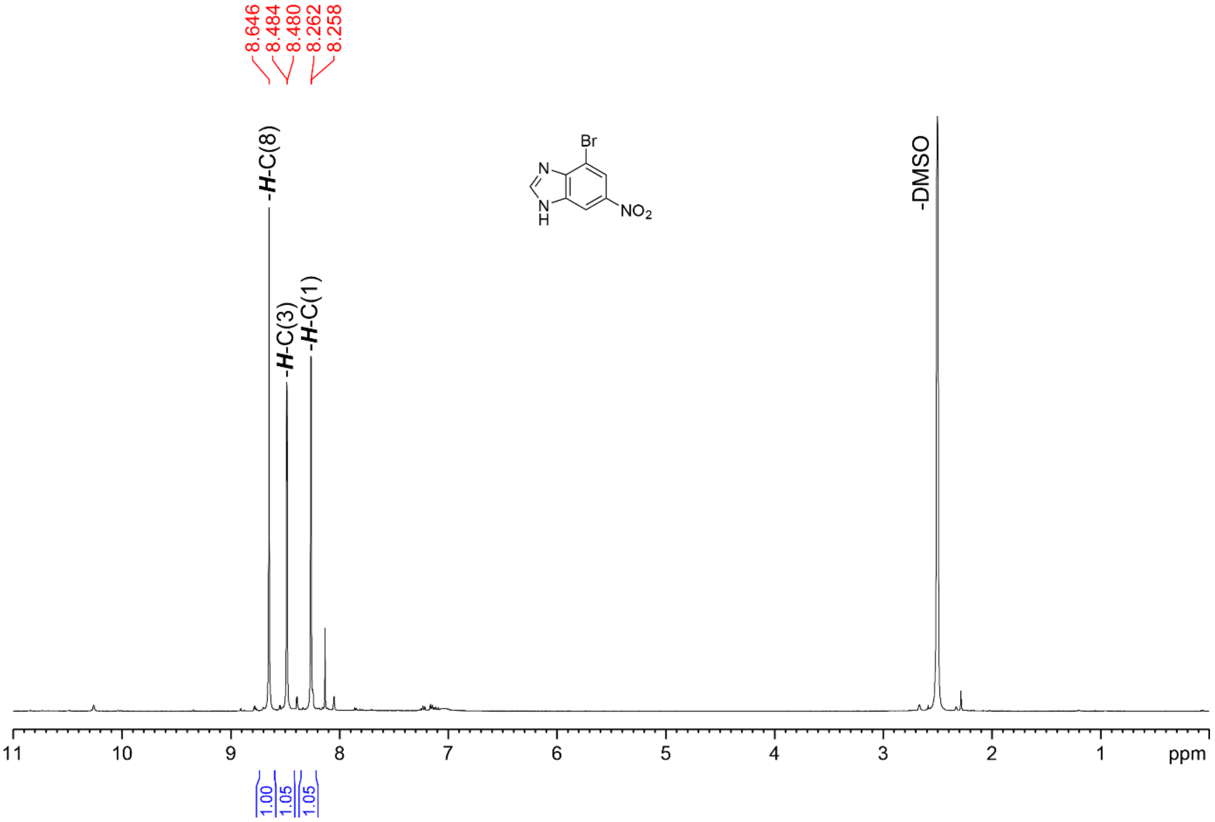

^{13}C-NMR (100 MHz, DMSO-d$_6$, 25 °C):

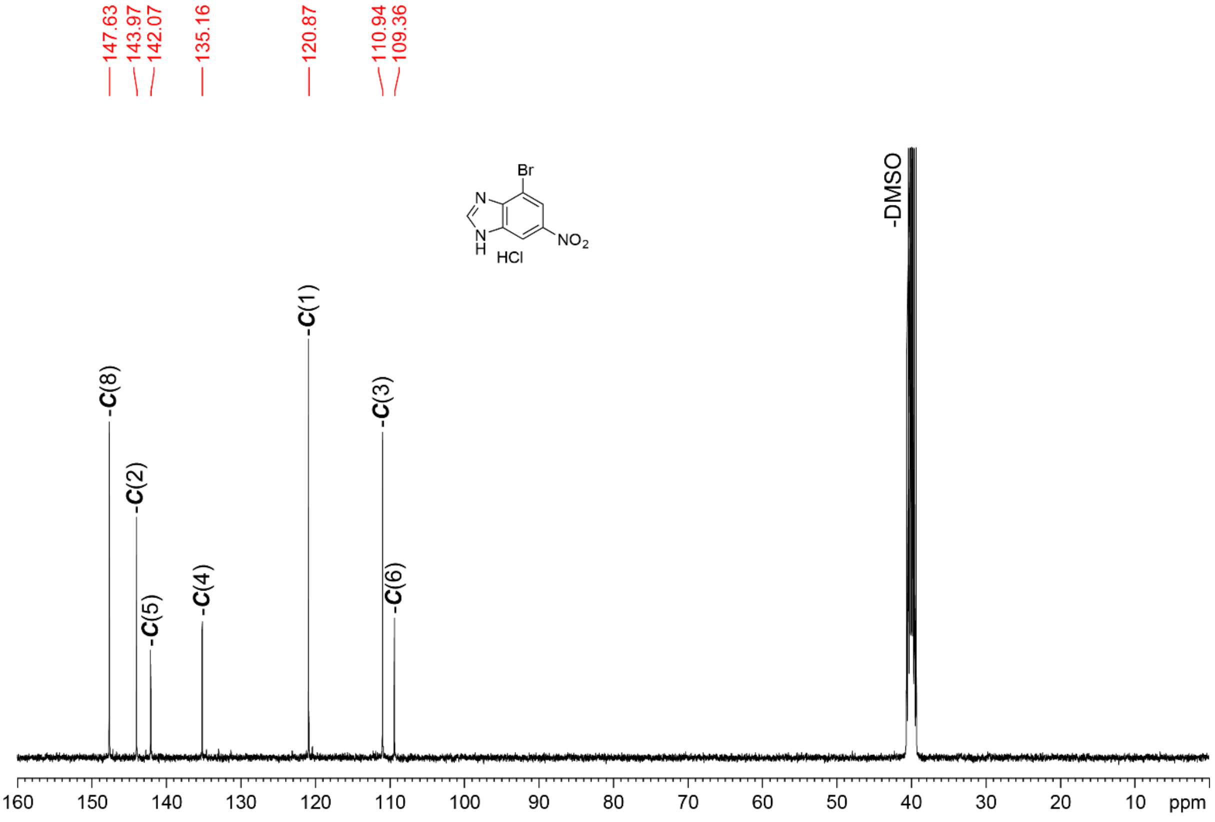

6-Bromo-2-nitro-9-(2',3',5'-tri-O-acetyl-ß-D-ribofuranosyl)-1,3-dideazapurine (3)

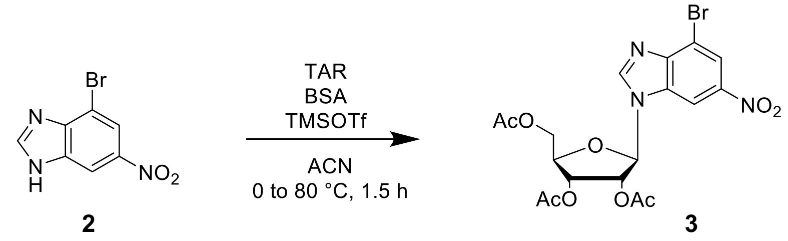

Compound **2** (460 mg, 1.90 mmol) was suspended in dry acetonitrile (15 mL) and *N*,*O*-bis(trimethylsilyl)acetamide (432 mg, 512 µL, 2.09 mmol) was added at room temperature. The reaction mixture was heated up to 80 °C and stirred for 2 hours. After cooling to 0 °C, 1,2,3,5-tetra-*O*-acetyl-ß-D-ribofuranose (665 mg, 2.09 mmol) was added and trimethylsilyl trifluoromethane-sulfonate (465 mg, 378 µL, 2.09 mmol) was added dropwise to the solution. The reaction solution was heated up again to 80 °C and refluxed for one and a half hour. Afterwards, the solvent and all volatiles were evaporated and the oily residue was diluted in dichloromethane, washed twice with saturated sodium bicarbonate solution and brine. The organic layer was dried over sodium sulfate and the crude product was purified by column chromatography using a gradient of 0 to 5% acetone in dichloromethane to yield compound **3**.

Yield: 771 mg of compound **3** as a slightly yellow foam (81%)
TLC (dichloromethane/acetone, 92/8)**:** R_f = 0.48
HR-ESI-MS (m/z): $[M+H]^+$ calcd. 501.0200; found 501.01980

^{1}H-NMR (400 MHz, $CDCl_3$, 25 °C):
δ = 2.06 (3H, s, C-C***H₃***); 2.16 (6H, s, 2x C-C***H₃***); 4.34-4.38 (1H, m, ***H(a)***-C(5')); 4.44-4.49 (2H, d, ***H***-C(4') & ***H(b)***-C(5')); 5.38-5.44 (1H, m, ***H***-C(3')); 5.48 (1H, t, J=5.86 Hz, ***H***-C(2')); 6.09 (1H, d, J=6.16 Hz, ***H***-C(1')); 8.39 (1H, s, ***H***-C(8)); 8.42 (1H, s, ***H***-C(1)); 8.55 (1H, s, ***H***-C(3)) ppm.

^{13}C-NMR (100 MHz, $CDCl_3$, 25 °C):
δ = 20.33-20.78 (3x***C***H_3); 62.71 (***C***(5')); 70.18 (***C***(3')); 73.48 (***C***(2')); 81.14 (***C***(4')); 87.48 (***C***(1')); 107.20 (***C***(3)); 114.43 (***C***(6)); 121.81 (***C***(1)); 131.69 (***C***(4)); 144.49 (***C***(2)); 145.02 (***C***(8)); 147.17 (***C***(5)); 169.42-170.23 (3x ***C***O) ppm.

^{1}H-NMR (400 MHz, CDCl$_3$, 25 °C):

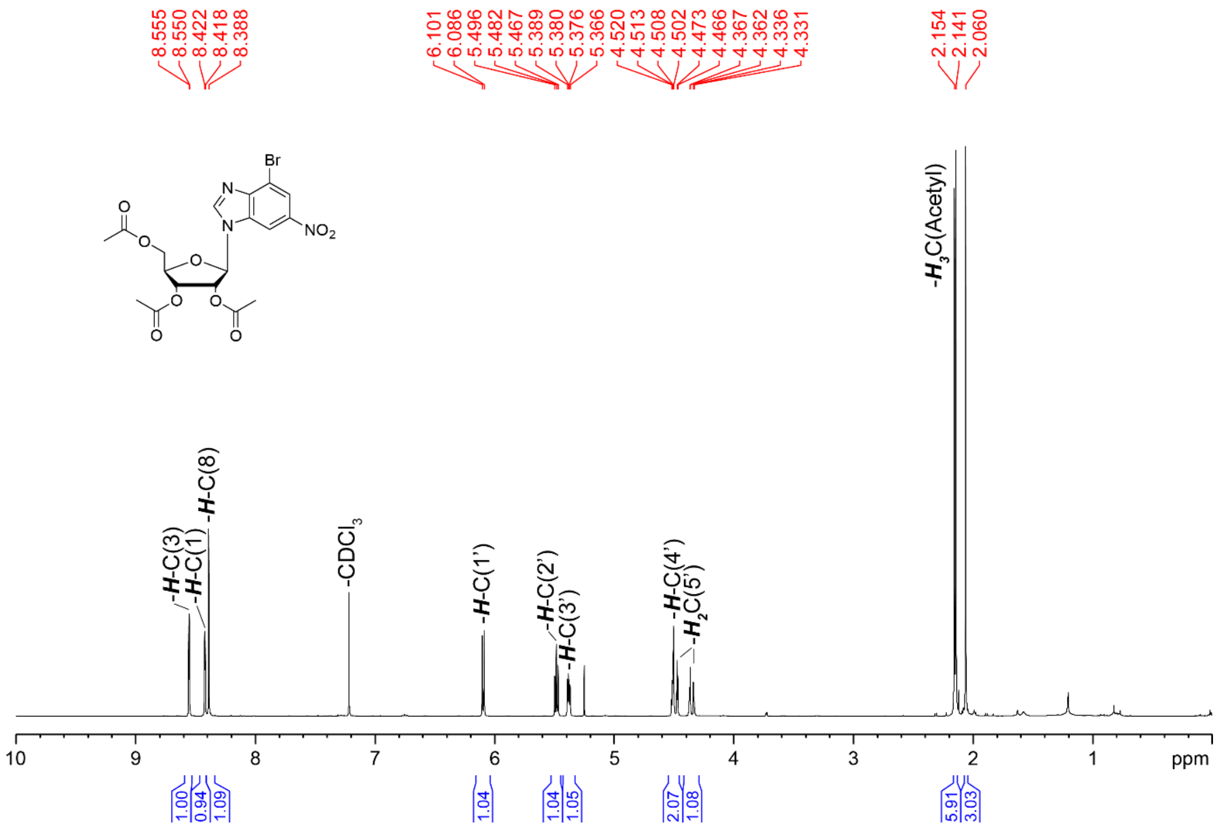

^{13}C-NMR (100 MHz, CDCl$_3$, 25 °C):

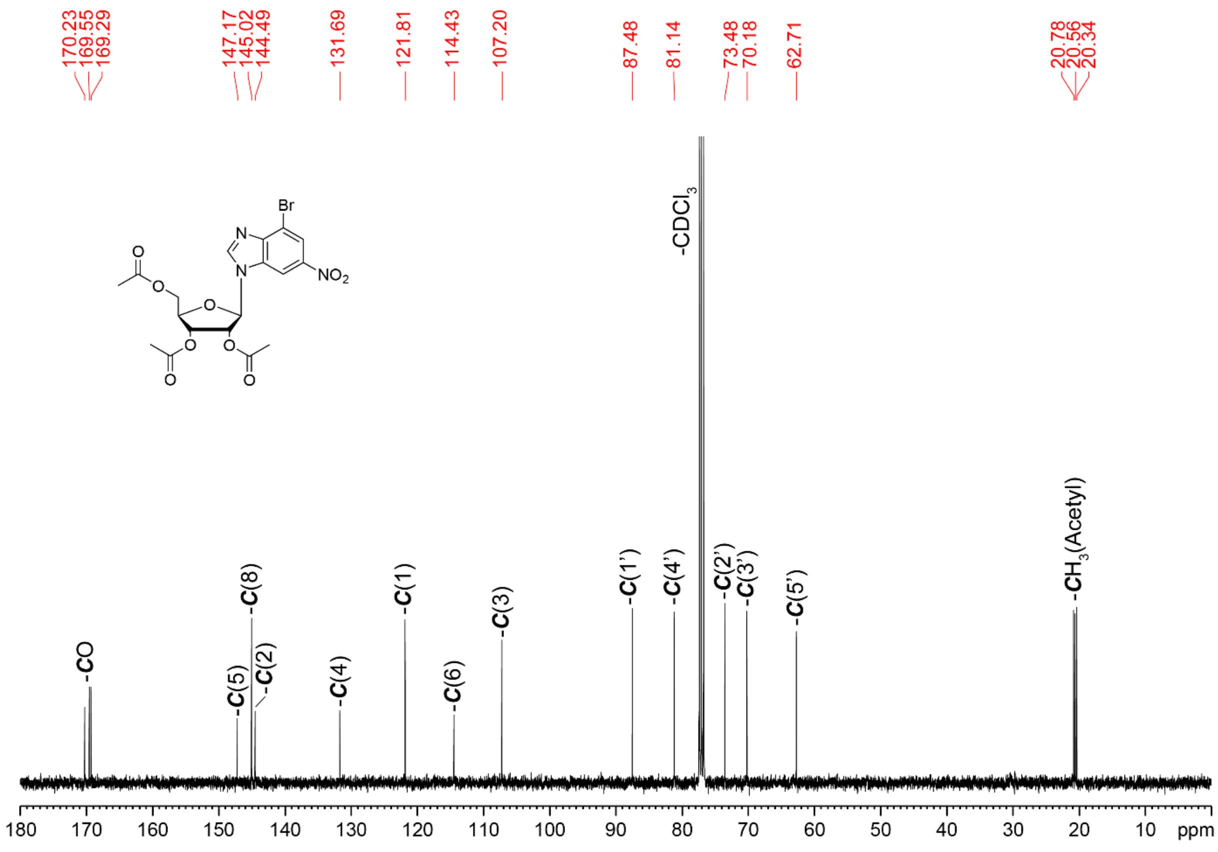

^{1}H-NMR, ^{13}C-HMBC NMR (400 MHz, $CDCl_3$, 25 °C):

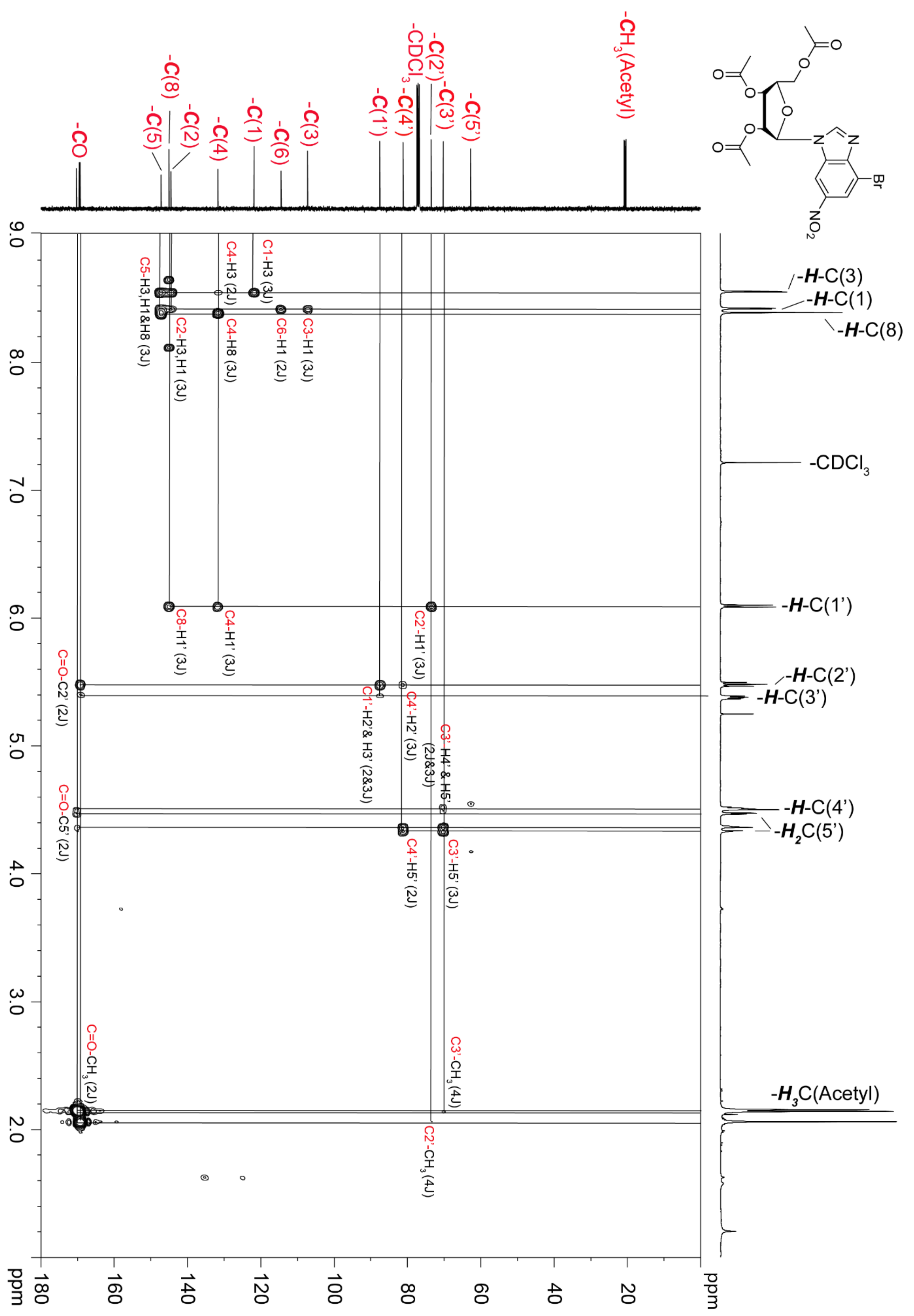

6-Bromo-2-nitro-9-[2',3',5'-*O*-tris(tert.-butyldimethylsilyl)-*ß*-D-ribofuranosyl]-1,3-dideazapurine (4)

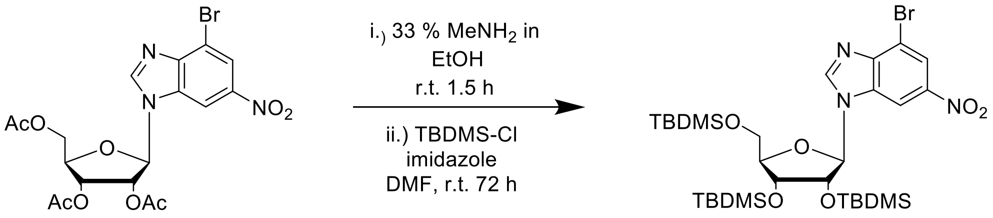

3 **4**

Compound **3** (6.40 g, 12.79 mmol) was dissolved in a solution of 33 wt% methylamine in ethanol (42 mL) and stirred at room temperature for one and a half hour. After complete consumption of the starting material, the mixture was diluted with dichloromethane and the solvents were evaporated. The crude product (TLC: dichloromethane/methanol, 92:8; R_f = 0.5) was used without further purifications. It was dissolved in DMF (70 mL) and *tert*-butyldimethylsilyl chloride (TBDMS-Cl, 11.58 g, 76.81 mmol) together with imidazole (6.97 g, 79.59 mmol) were added. The resulting mixture was stirred for 48 hours at room temperature. Then, the solvent was evaporated and the resulting oily residue was diluted with ethyl acetate, washed once with water, two times with brine and dried over sodium sulfate. The crude product was purified by column chromatography using a gradient of 0 to 10% ethyl acetate in cyclohexane to yield compound **4**.

Yield: 7.82 g of compound **4** as a white foam (85%)
TLC (dichloromethane/methanol, 99/1)**:** R_f = 0.63
HR-ESI-MS (m/z): $[M+H]^+$ calcd. 716.2532; found 716.2554

^{1}H-NMR (400 MHz, $CDCl_3$, 25 °C):
δ = -0.59 (3H, s, Si-C***H₃***); -0.11 (3H, s, Si-C***H₃***); 0.14 (12H, m, Si-C***H₃***); 0.73 (9H, s, Si-C(C***H₃***)₃); 0.96 (18H, d, J=4.44 Hz, Si-C(C***H₃***)₃); 3.86-3.97 (2H, m, ***H(a)*** & ***H(b)***-C(5')); 4.19 (1H, d, J=1.80 Hz, ***H***-C(4')); 4.23 (1H, d, J=1.80 Hz, ***H***-C(3')); 4.36 (1H, q, J=3.89 Hz, ***H***-C(2')); 5.92 (1H, d, J=6.16 Hz, ***H***-C(1')); 8.43 (1H, s, ***H***-C(1)); 8.48 (2H, m, ***H***-C(3) & ***H***-C(8)) ppm.

^{13}C-NMR (100 MHz, $CDCl_3$, 25 °C):
δ = -5.08 (***C***H_3-Si-***C***H_3); 18.15 (Si-***C***$(CH_3)_3$); 25.87 (Si-C(***C***$H_3)_3$; 63.32 (***C***(5')); 73.02 (***C***(3')); 77.71 (***C***(2')); 87.58 (***C***(4')); 89.21 (***C***(1')); 107.45 (***C***(3)); 113.90 (***C***(6)); 121.23 (***C***(1)); 132.32 (***C***(4)); 144.03 (***C***(2)); 145.78 (***C***(8)); 146.80 (***C***(5)) ppm.

^{1}H-NMR (400 MHz, $CDCl_3$, 25 °C):

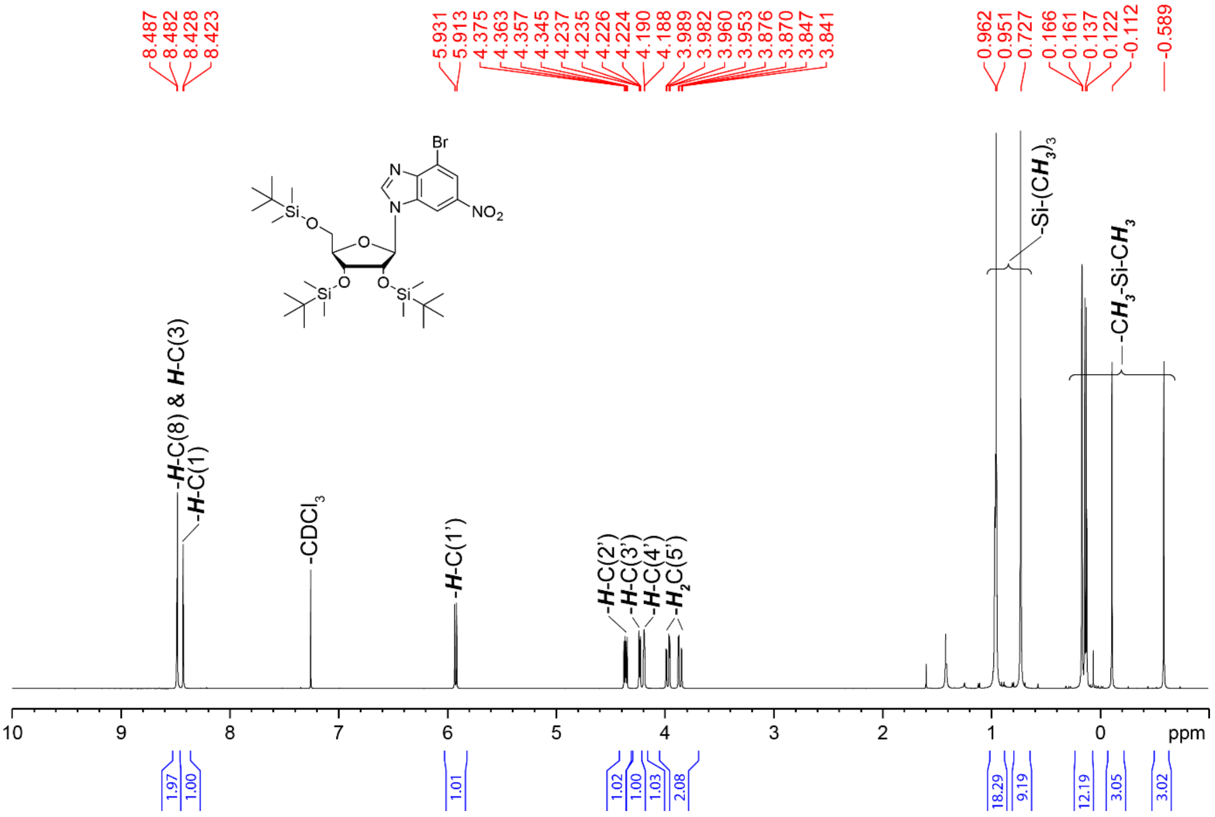

^{13}C-NMR (100 MHz, $CDCl_3$, 25 °C):

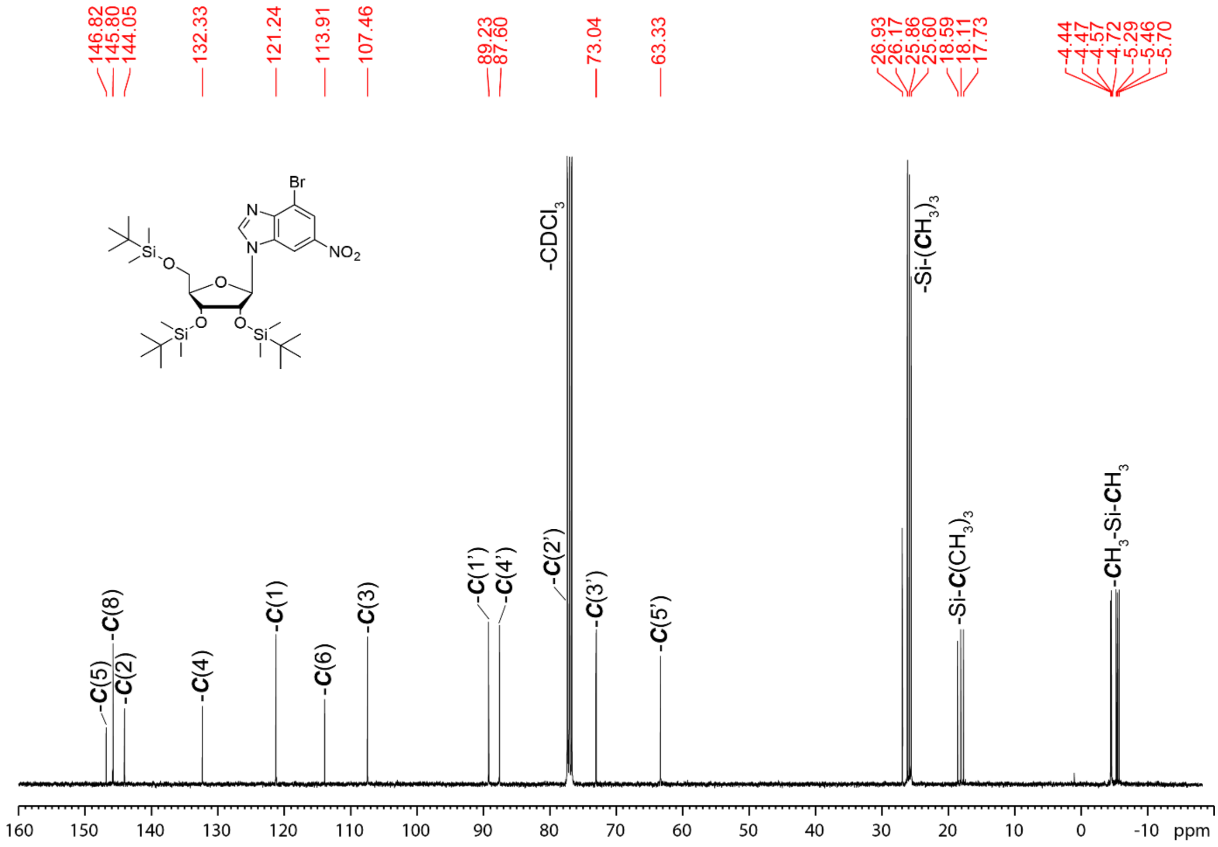

6-Benzyloxy-2-nitro-9-[2',3',5'-*O*-tris(*tert*-butyldimethylsilyl)-*ß*-D-ribofuranosyl]-1,3-dideazapurine (5)

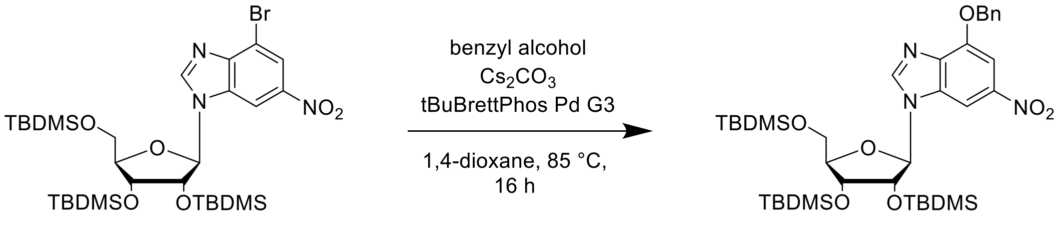

An oven dried flask was charged with Cs_2CO_3 (218 mg, 669 µmol). The flask was placed under vacuum and heated at 450 °C with a heat gun for around 3 minutes, or until the Cs_2CO_3 stopped moving. Afterwards, tBuBrettPhos Pd G3 (10.7 mg, 3 mmol %) was added under argon atmosphere and dried overnight at 45 °C under high vacuum. The flask was then charged with dry aryl bromide **4** (300 mg, 418 mmol) under argon atmosphere, evacuated and backfilled with argon three times. Dry degassed 1,4-dioxane (0.3 mL, 1 M) was added via syringe and the septum was wrapped completely with parafilm. The reaction mixture was sonicated for one minute, and stirred for 10 min at 80 °C in a preheated oil bath. Subsequently, benzyl alcohol (543 mg, 522 µL, 502 mmol) was placed in the sealed tube via syringe and stirring was continued until TLC showed full conversion. After completion, the reaction mixture was cooled to room temperature, diluted with dichloromethane and filtered over celite. The crude product was purified by column chromatography using a gradient of 0 to 10% ethyl acetate in cyclohexane as gradient to yield compound **5**.

Yield: 290 mg of compound **5** as a white foam (92%)
TLC (cyclohexane/ethyl acetate, 8/2)**:** R_f = 0.58
HR-ESI-MS (m/z): $[M+H]^+$ calcd. 743.3812; found 743.3832

^{1}H-NMR (400 MHz, $CDCl_3$, 25 °C):
δ = -0.55 (3H, s, Si-C***H₃***); -0.11 (3H, s, Si-C***H₃***); 0.15 (12H, m, Si-C***H₃***); 0.74 (9H, s, Si-C(C***H₃***)₃); 0.97 (18H, d, J=4.44 Hz, Si-C(C***H₃***)₃); 3.84-3.99 (2H, m, ***H(a)*** & ***H(b)***-C(5')); 4.18 (1H, d, J=1.80 Hz, ***H***-C(4')); 4.24 (1H, t, J=2.20 Hz, ***H***-C(3')); 4.37 (1H, q, J=3.81 Hz, ***H***-C(2')); 5.43 (1H, s, C***H₂***(benzyl)); 5.92 (1H, d, J=7.00 Hz, ***H***-C(1')); 7.29-7.53 (5H, m, ***H***-C(benzyl)); 7.69 (1H, s, ***H***-C(1)); 8.15 (1H, s, ***H***-C(3)); 8.37 (1H, m, ***H***-C(8)) ppm.

^{13}C-NMR (100 MHz, $CDCl_3$, 25 °C):
δ = -5.05 (***C***H₃-Si-***C***H₃); 18.16 (Si-***C***(CH₃)₃); 25.89 (Si-C(***C***H₃)₃; 63.31 (***C***(5')); 71.22 (***C***H₂(benzyl)); 72.95 (***C***(3')); 77.05 (***C***(2')); 87.15 (***C***(4')); 88.93 (***C***(1')); 100.88 (***C***(3)); 101.51 (***C***(1)); 128.21 (***C***H(benzyl)); 133.48 (***C***(4)); 135.90 (***C***(benzyl)); 138.79 (***C***(5)); 143.91 (***C***(8)); 144.56 (***C***(2)); 150.26 (***C***(6)) ppm.

^{1}H-NMR (400 MHz, $CDCl_3$, 25 °C):

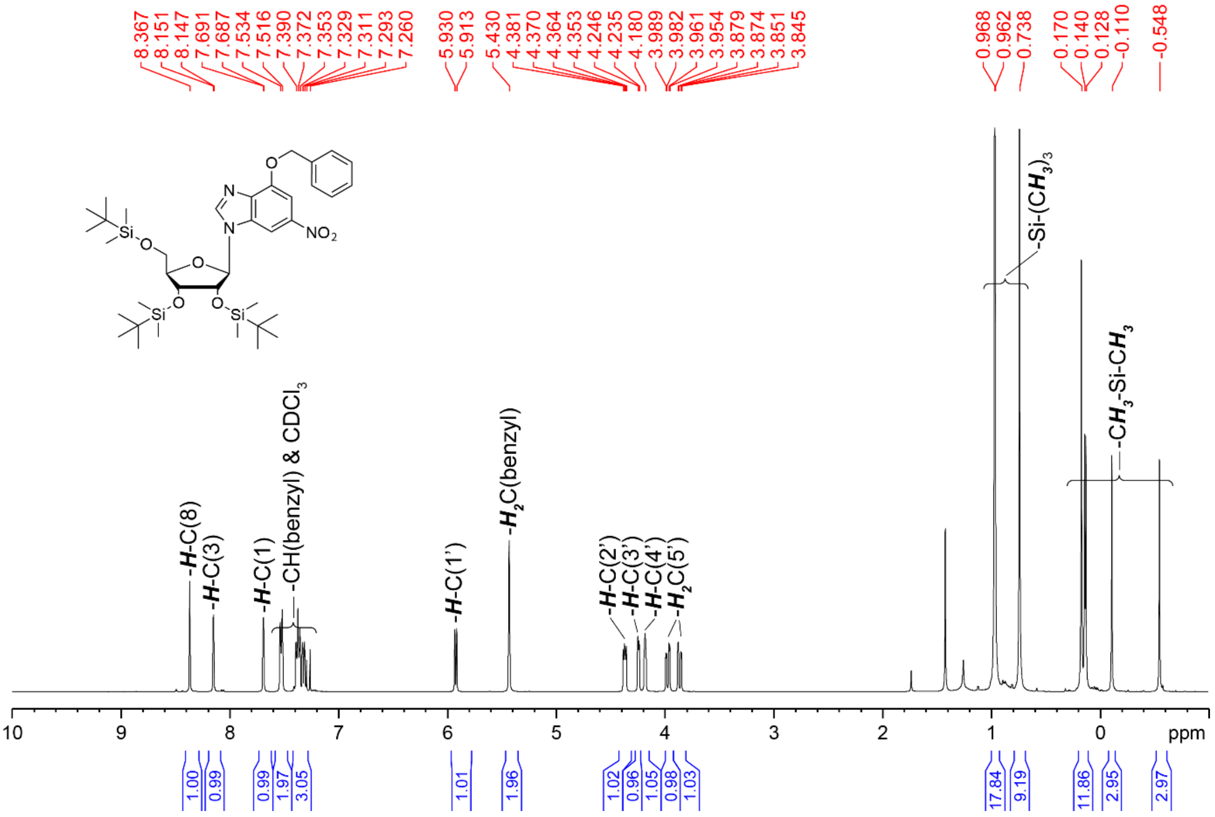

^{13}C-NMR (100 MHz, $CDCl_3$, 25 °C):

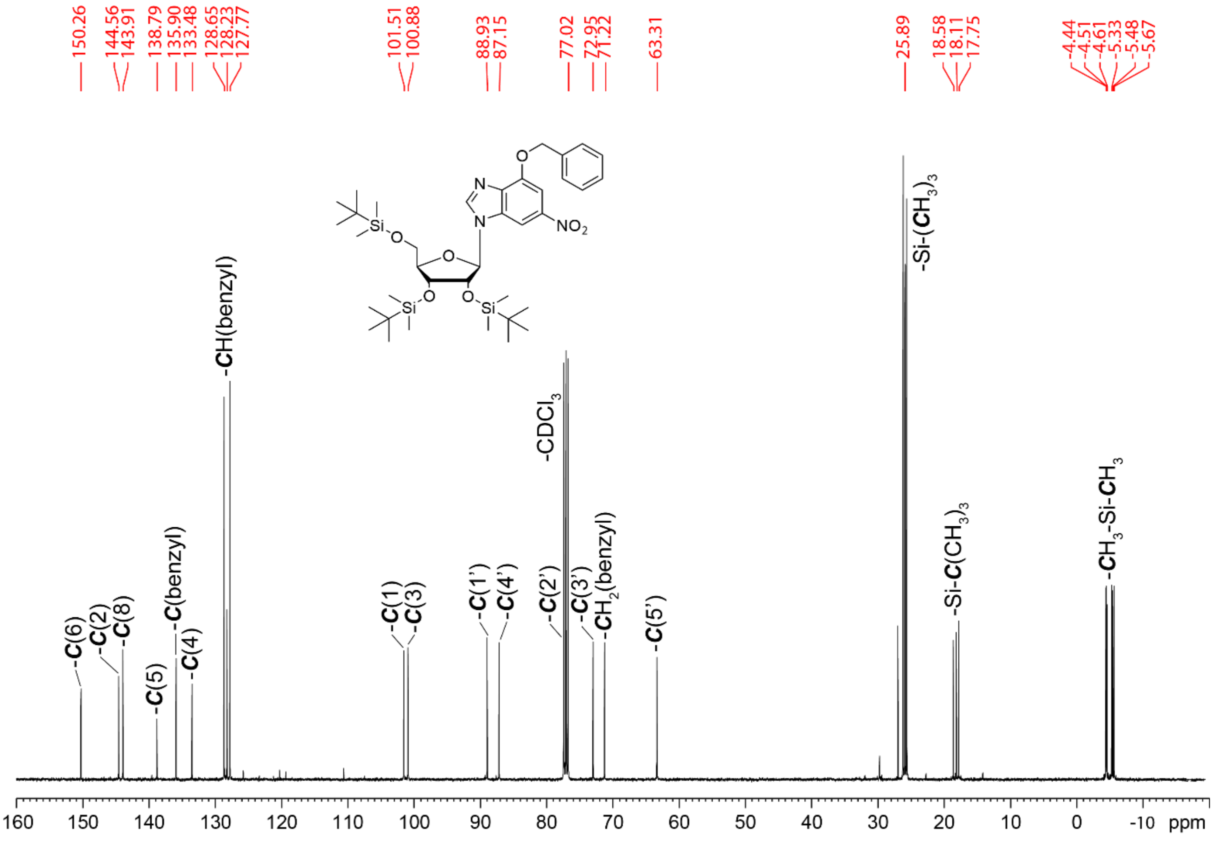

1,3-Dideazaguanosine (6)

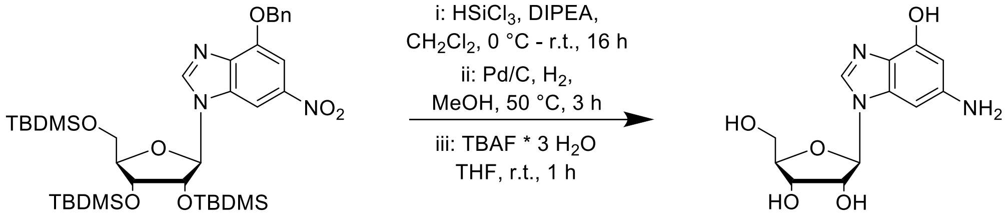

5 **6**

Step i: Compound **5** (1.00 g, 1.34 mmol) was dissolved in dry dichloromethane (11 mL) at 0 °C, and *N*,*N*-diisopropylethylamine (DIPEA, 868 mg, 1.17 mL, 6.72 mmol) was added. Subsequently, a solution made of dry dichloromethane (3.4 mL) and trichlorosilane ($HSiCl_3$, 637 mg, 505 µL, 4.70 mmol) at 0 °C was added dropwise over a period of 10 minutes. After 30 minutes, TLC showed complete consumption of the starting material. The reaction mixture was diluted with dichloromethane and quenched by adding saturated sodium bicarbonate solution. This mixture was allowed to stir for another hour. The resulting suspension was filtered off and the collected layers were transferred into a separatory funnel. The organic layer was collected and the aqueous phase was extracted two times with dichloromethane. The combined organic layers were washed with brine, dried over sodium sulfate and evaporated to dryness. The crude product was purified with silica gel chromatography using a gradient of 0 to 15% ethyl acetate in cyclohexane to yield the amine intermediate (TLC (dichloromethane/methanol 98/2): R_f = 0.56)

Step ii: The amine intermediate (750 mg, 1.05 mmol; obtained from step i) and palladium on carbon 10 % (Pd/C, 150 mg) were suspended in methanol (20 mL) under argon atmosphere. A rubber septum was applied and hydrogen gas (balloon with syringe) was bubbled through the solution for 10 minutes. Stirring was continued under hydrogen atmosphere for further two hours at 50 °C. The catalyst was filtered off, and the filtrate was evaporated to dryness to yield the phenol intermediate was obtained (~93 %) and used without further purification in the next step.

Step iii: The phenol intermediate (610 mg, 977 mmol; obtained from step ii), and tetra-n-butylammonium fluoride trihydrate (TBAF * $3H_2O$, 541 mg, 1.72 mmol) were dissolved in tetrahydrofuran (9 mL) and stirred at room temperature for two hours. Afterwards, the solvent was removed and the crude product was purified by reversed phase chromatography (Lichroprep RP-18 (40-63 µm, ÄktaPrime, run time: 30 min) using a gradient of 0 to 20 % acetonitrile in water to obtain compound **6**.

Yield: 206 mg of compound **6** as a pink solid (55%)
TLC (dichloromethane/methanol, 8/2)**:** R_f = 0.28
HR-ESI-MS (m/z): $[M+H]^+$ calcd. 282.1084; found 282.1079

^{1}H-NMR (400 MHz, $CDCl_3$, 25 °C):
δ = 3.51-3.68 (2H, m, ***H(a)*** & ***H(b)***-C(5')); 3.90 (1H, d, J=3.78 Hz, ***H***-C(4')); 4.06 (1H, t, J=4.26 Hz, ***H***-C(3')); 4.30 (1H, t, J=5.42 Hz, ***H***-C(2')); 4.83 (2H, s, ***H*₂**N); 4.93-5.51 (3H, m, O***H***(5‘, 3‘, 2‘)); 5.57 (1H, d, J=5.81 Hz, ***H***-C(1')); 5.97 (1H, s, ***H***-C(1)); 6.13 (1H, s, ***H***-C(3)); 7.95 (1H, m, ***H***-C(8)); 8.03-9.45 (1H, s, O***H***(aromat.)) ppm.

^{13}C-NMR (100 MHz, $CDCl_3$, 25 °C):

δ = 61.79 (***C***(5’)); 70.55 (***C***(3’)); 73.65 (***C***(2’)); 85.24 (***C***(4’)); 86.25 (***C***(3)); 88.65 (***C***(1’)); 97.40 (***C***(1)); 126.20 (***C***(5)); 136.57 (***C***(4)); 137.69 (***C***(8)); 146.54 (***C***(2)); 149.59 (***C***(6)) ppm.

^{1}H-NMR (400 MHz, $CDCl_3$, 25 °C):

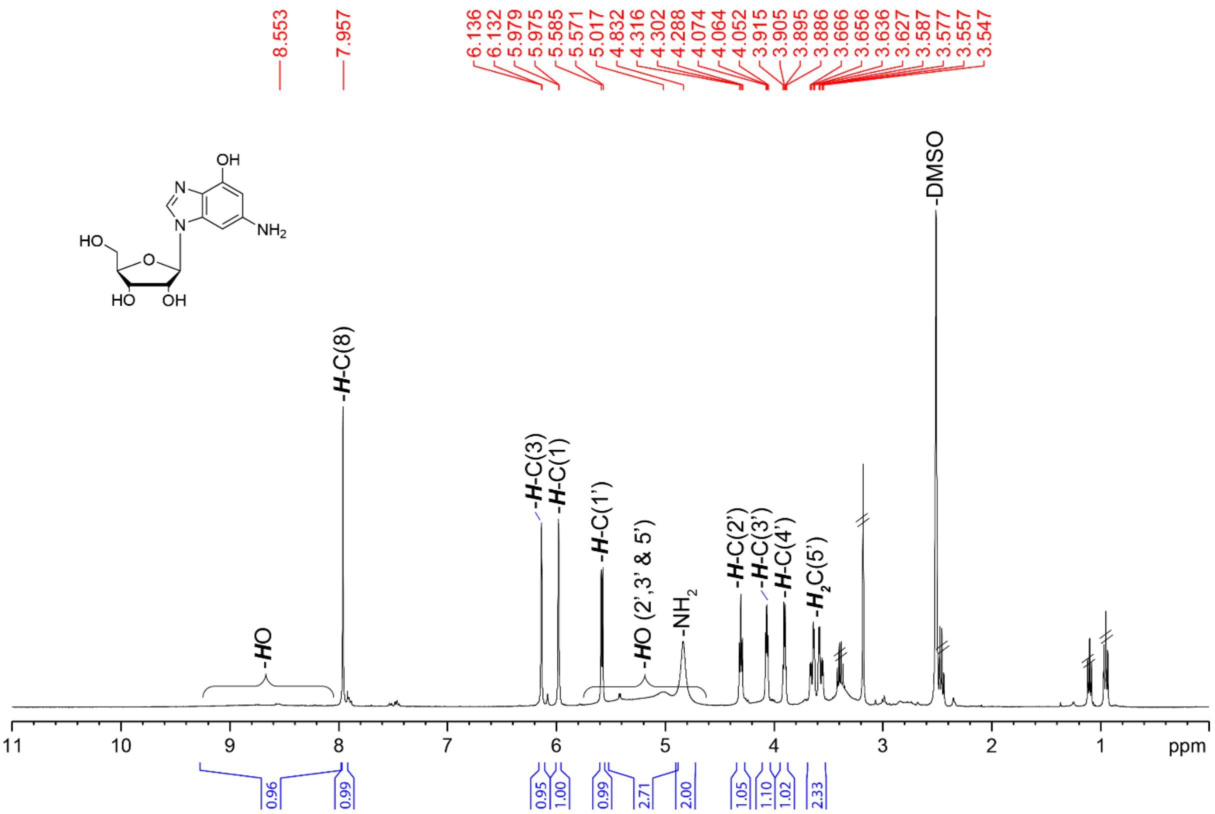

^{13}C-NMR (100 MHz, $CDCl_3$, 25 °C):

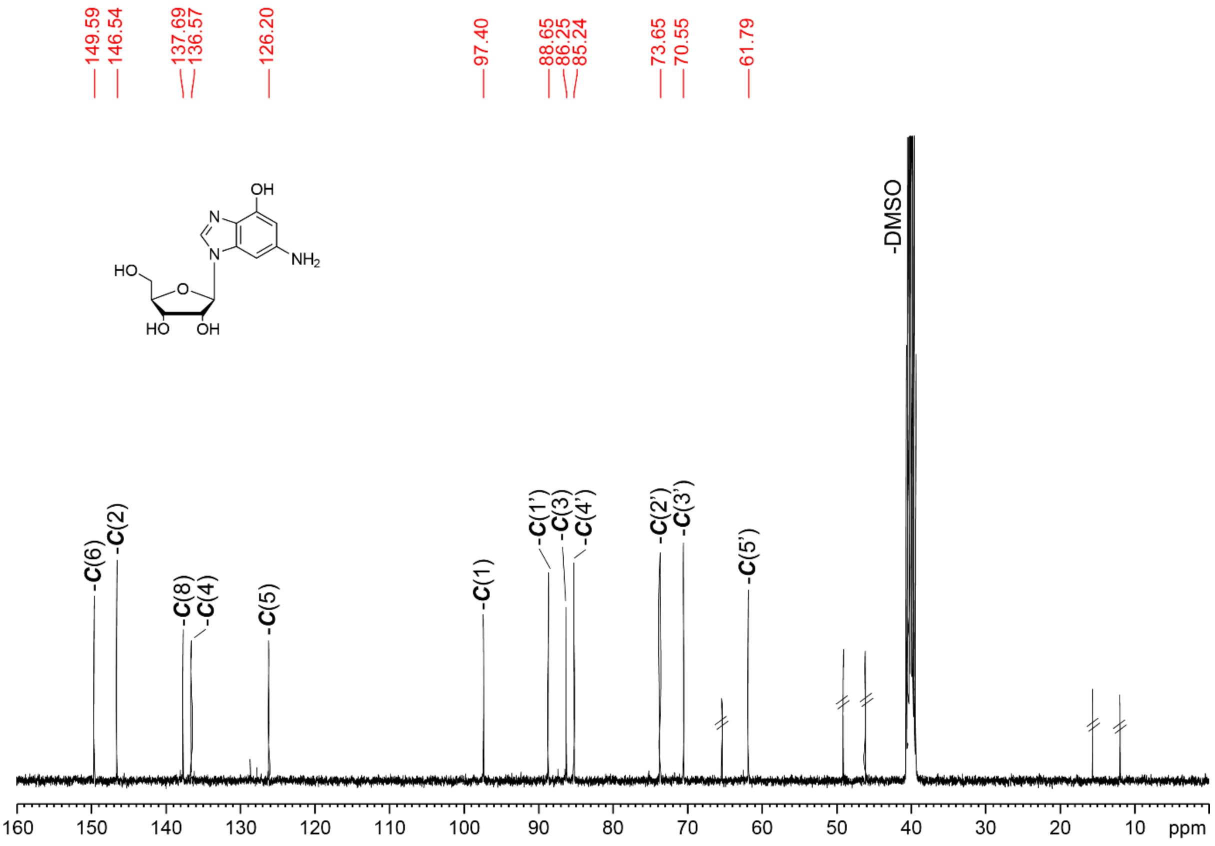

$^{1}H,^{13}C$-HMBC NMR (400 MHz, $CDCl_3$, 25 °C):

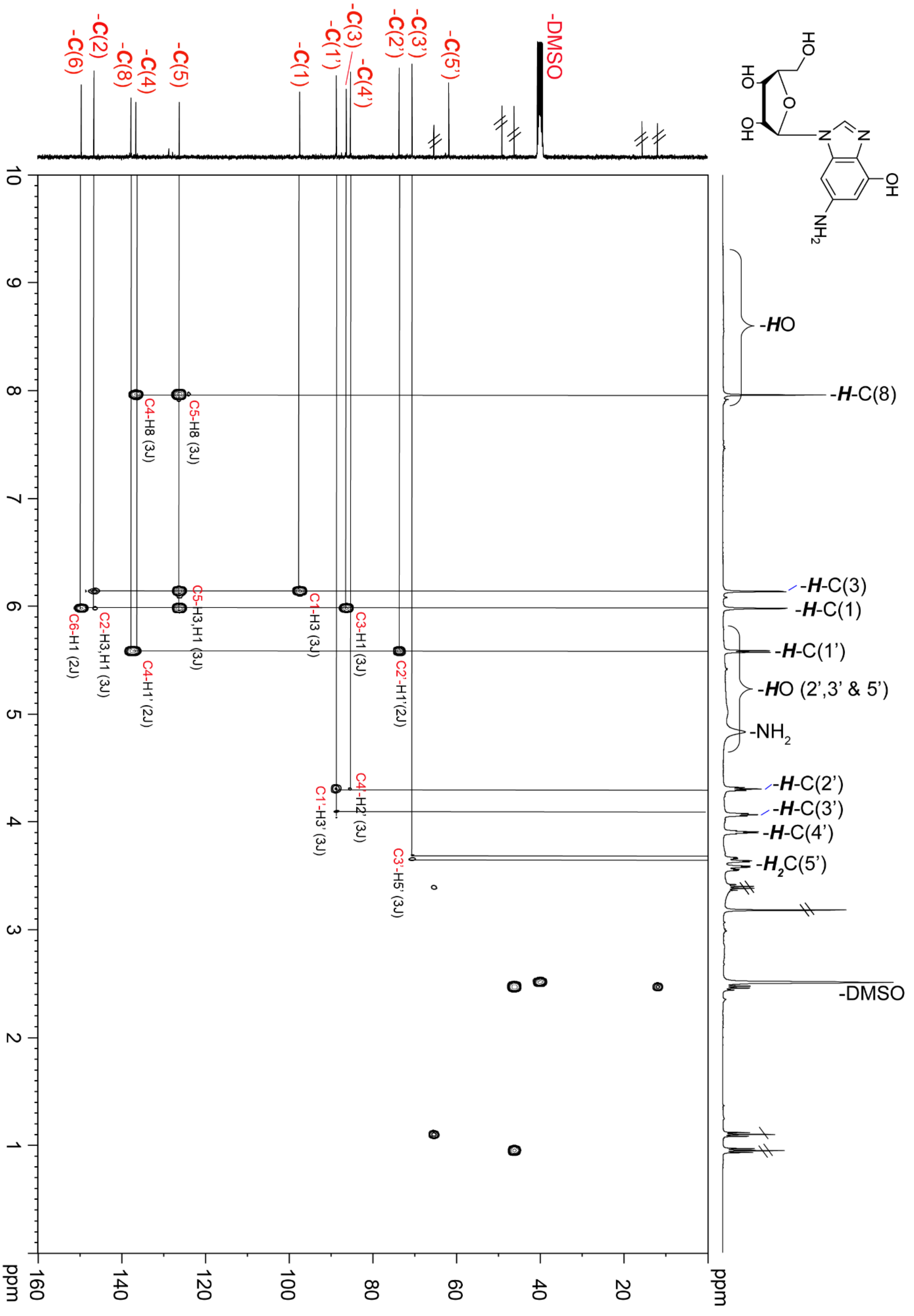

Synthesis of 1,3-dideazaguanosinephosphoramidite 12

***O*[6]-(Benzyl)-*N*[2]-trifluoroacetyl-2',3',5'-*O*-tris(*tert*-butyldimethylsilyl)-1,3-dideazaguanosine (7)**

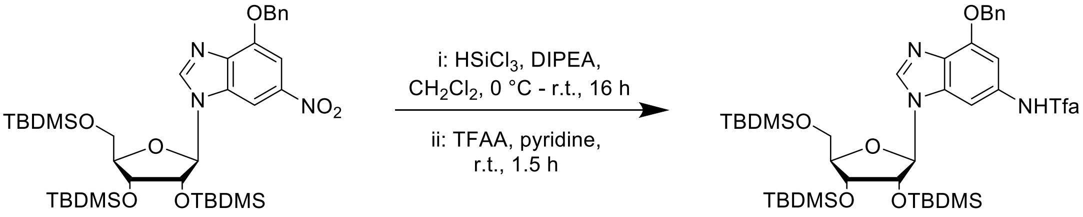

5 **7**

Step i: Compound **5** (550 mg, 0.77 mmol) was dissolved in dry dichloromethane (6 mL) at 0 °C, and *N*,*N*-diisopropylethylamine (DIPEA, 496 mg, 670 µL, 3.84 mmol) was added. Subsequently, a solution made of dry dichloromethane (1.7 mL) and trichlorosilane ($HSiCl_3$, 364 mg, 288 µL, 2.69 mmol) at 0 °C was added dropwise over a period of 10 minutes. After 30 minutes, TLC showed complete consumption of the starting material. The reaction mixture was diluted with dichloromethane (10 mL) and quenched by adding saturated sodium bicarbonate solution (10 mL). This mixture was allowed to stir for another hour. The resulting suspension was filtered off and the collected layers were transferred into a separatory funnel. The organic part was collected and the aqueous phase was extracted two times with dichloromethane. The combined organic layers were washed with brine, dried over sodium sulfate and evaporated to dryness. The residue was purified with silica gel chromatography using a gradient of 0 to 15% ethyl acetate in cyclohexane to obtain the amine intermediate (TLC (dichloromethane/ methanol 98/2): R_f = 0.56) that was directly used in the next step.

Step ii: The amine intermediate (413 mg, 603 µmol; obtained from step i), together with 4-dimethylaminopyrdine (spatula tip) was dissolved in dry pyridine (4.8 mL) and cooled to 0 °C. Then, trifluoroacetic anhydride (TFAA, 152 mg, 101 µL, 724 µmol) was added over a syringe in 10 minutes at 0 °C. The reaction mixture was stirred for 90 minutes at room temperature. Afterwards, the solvent was evaporated and the oily residue was dissolved in ethyl acetate and washed successively with 5% citric acid solution, saturated sodium bicarbonate solution and brine. The organic layer was dried over Na_2SO_4, filtered and evaporated to dryness. The crude product was purified with silica gel chromatography using a gradient of 0 to 15% ethyl acetate in cyclohexane to yield compound **7**.

Yield: 452 mg of compound **7** as a purple foam (71%)
TLC (cyclohexane/ethyl acetate, 8/2)**:** R_f = 0.63
HR-ESI-MS (m/z): $[M+H]^+$ calcd. 810.3971; found 810.3952

^{1}H-NMR (400 MHz, $CDCl_3$, 25 °C):
δ = -0.52 (3H, s, Si-C***H₃***); -0.13 (3H, s, Si-C***H₃***); 0.12 (12H, m, Si-C***H₃***); 0.74 (9H, s, Si-C(C***H₃***)₃); 0.94 (18H, d, J=4.44 Hz, Si-C(C***H₃***)₃); 3.84-3.94 (2H, m, ***H(a)*** & ***H(b)***-C(5')); 4.13 (1H, m, ***H***-C(4')); 4.23 (1H, q, J=1.95 Hz, ***H***-C(3')); 4.38 (1H, q, J=3.80 Hz, ***H***-C(2')); 5.30 (2H, s, C***H₂***(benzyl)); 5.84 (1H, d, J=6.64 Hz, ***H***-C(1')); 7.25-7.44 (5H, m, ***H***-C(benzyl)); 7.58 (1H, d, J=1.64 Hz, ***H***-C(1)); 8.13 (1H, s, ***H***-C(8)); 8.23 (1H, s, N***H***) ppm.

^{13}C-NMR (100 MHz, $CDCl_3$, 25 °C):

δ = -5.02 (***C***H_3-Si-***C***H_3); 18.14 (Si-***C***(CH_3)$_3$); 25.87 (Si-C(***C***H_3)$_3$; 63.340 (***C***(5')); 70.88 (***C***H_2-benzyl)); 72.87 (***C***(3')); 75.97 (***C***(2')); 86.54 (***C***(4')); 88.70 (***C***(1')); 96.66 (***C***(3)); 99.56 (***C***(1)); 114.37-117.24 (***C***F_3); 127.48-128.65 (***C***H(benzyl)); 131.51 (***C***(2)); 132.74 (***C***(5)); 134.56 (***C***(4)); 136.51 (***C***(benzyl)); 141.03 (***C***(8)); 150.72 (***C***(6)); 154.74 (***C***O) ppm.

^{1}H-NMR (400 MHz, $CDCl_3$, 25 °C):

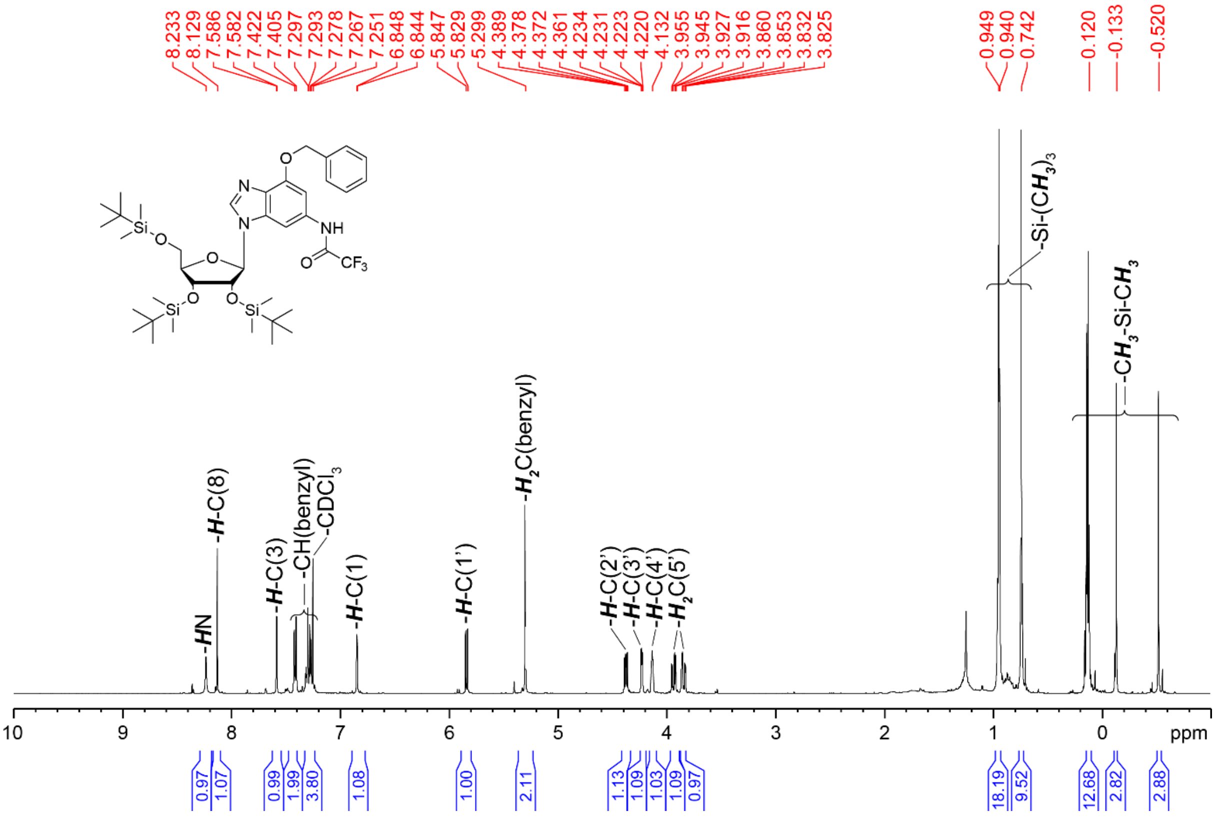

^{13}C-NMR (100 MHz, $CDCl_3$, 25 °C):

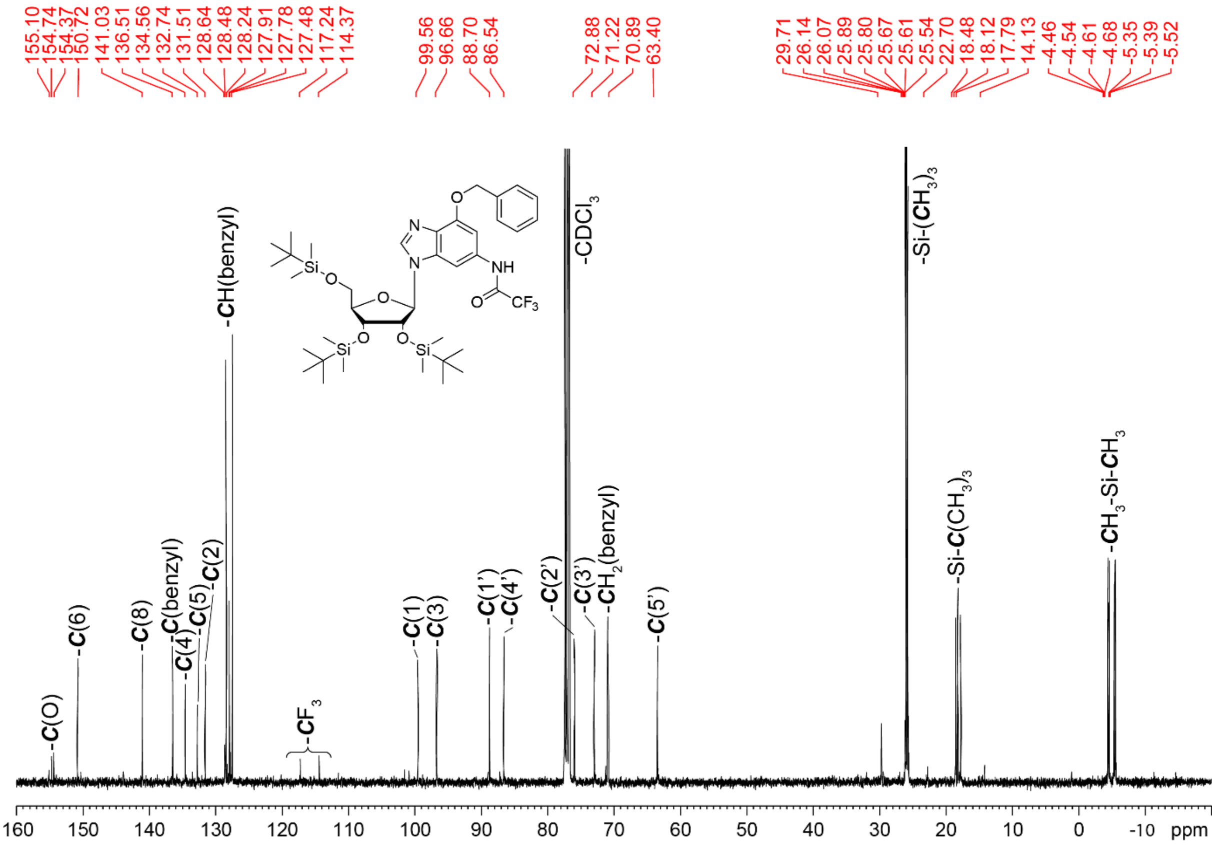

N^2-Trifluoroacetyl-2',3',5'-*O*-tris(*tert*-butyldimethylsilyl)-1,3-dideazaguanosine (8)

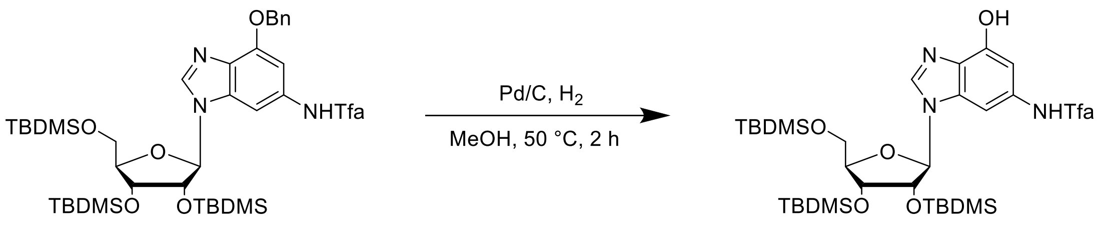

7 **8**

Compound **7** (2.50 mg, 3.09 mmol) and palladium on carbon 10% (Pd/C, 367 mg) were suspended in methanol (80 mL) under argon atmosphere. A rubber septum was applied and hydrogen gas (balloon with syringe) was bubbled through the solution for 10 minutes. Stirring was continued under hydrogen atmosphere for further two hours at 50 °C. The catalyst was filtered off, and the filtrate was evaporated to dryness to yield compound **8** without further purification.

Yield: 2.07 g of compound **8** as a purple foam (93%)
TLC (cyclohexane/ethyl acetate, 8/2)**:** R_f = 0.58
HR-ESI-MS (m/z): $[M+H]^+$ calcd. 720.3502; found 720.3486

^{1}H-NMR (400 MHz, $CDCl_3$, 25 °C):
δ = -0.52 (3H, s, Si-C***H₃***); -0.13 (3H, s, Si-C***H₃***); 0.15 (12H, m, Si-C***H₃***); 0.74 (9H, s, Si-C(C***H₃***)₃); 0.95 (18H, d, J=4.44 Hz, Si-C(C***H₃***)₃); 3.84-3.96 (2H, m, ***H(a)*** & ***H(b)***-C(5')); 4.14 (1H, m, ***H***-C(4')); 4.23 (1H, q, J=1.95 Hz, ***H***-C(3')); 4.38 (1H, q, J=3.80 Hz, ***H***-C(2')); 5.86 (1H, d, J=6.64 Hz, ***H***-C(1')); 7.60 (1H, d, J=1.64 Hz, ***H***-C(1)); 7.84 (1H, s, N***H***); 8.26 (1H, s, ***H***-C(8)); 11.46 (1H, b, O***H***) ppm.

^{13}C-NMR (100 MHz, $CDCl_3$, 25 °C):
δ = -5.04 (***C***H_3-Si-***C***H_3); 18.14 (Si-***C***$(CH_3)_3$); 25.87 (Si-C(***C***$H_3)_3$; 63.36 (***C***(5')); 72.86 (***C***(3')); 76.42 (***C***(2')); 86.62 (***C***(4')); 88.98 (***C***(1')); 94.68 (***C***(3)); 101.97 (***C***(1)); 114.37-120.11 (***C***F_3); 130.70 (***C***(5)); 132.17 (***C***(2)); 134.27 (***C***(4)); 140.91 (***C***(8)); 149.84 (***C***(6)); 154.38 (***C***O) ppm.

^{1}H-NMR (400 MHz, $CDCl_3$, 25 °C):

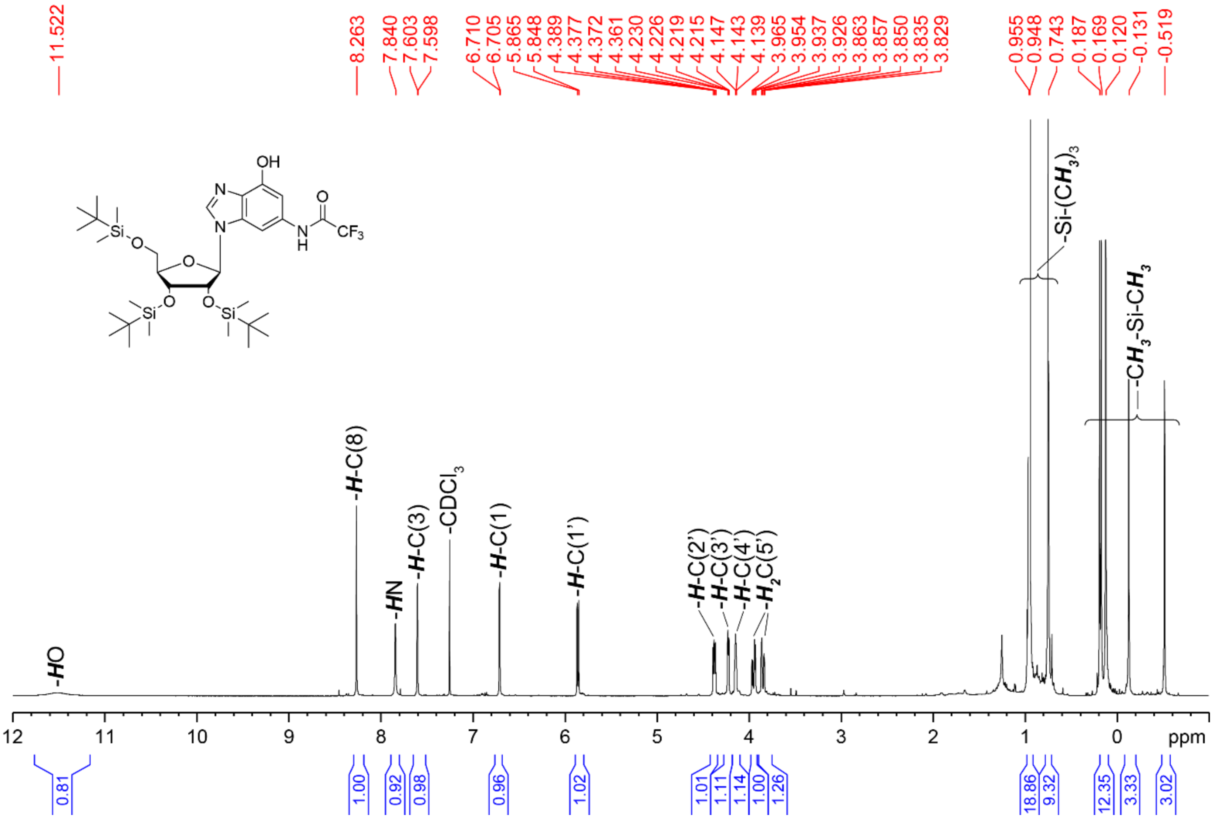

^{13}C-NMR (100 MHz, $CDCl_3$, 25 °C):

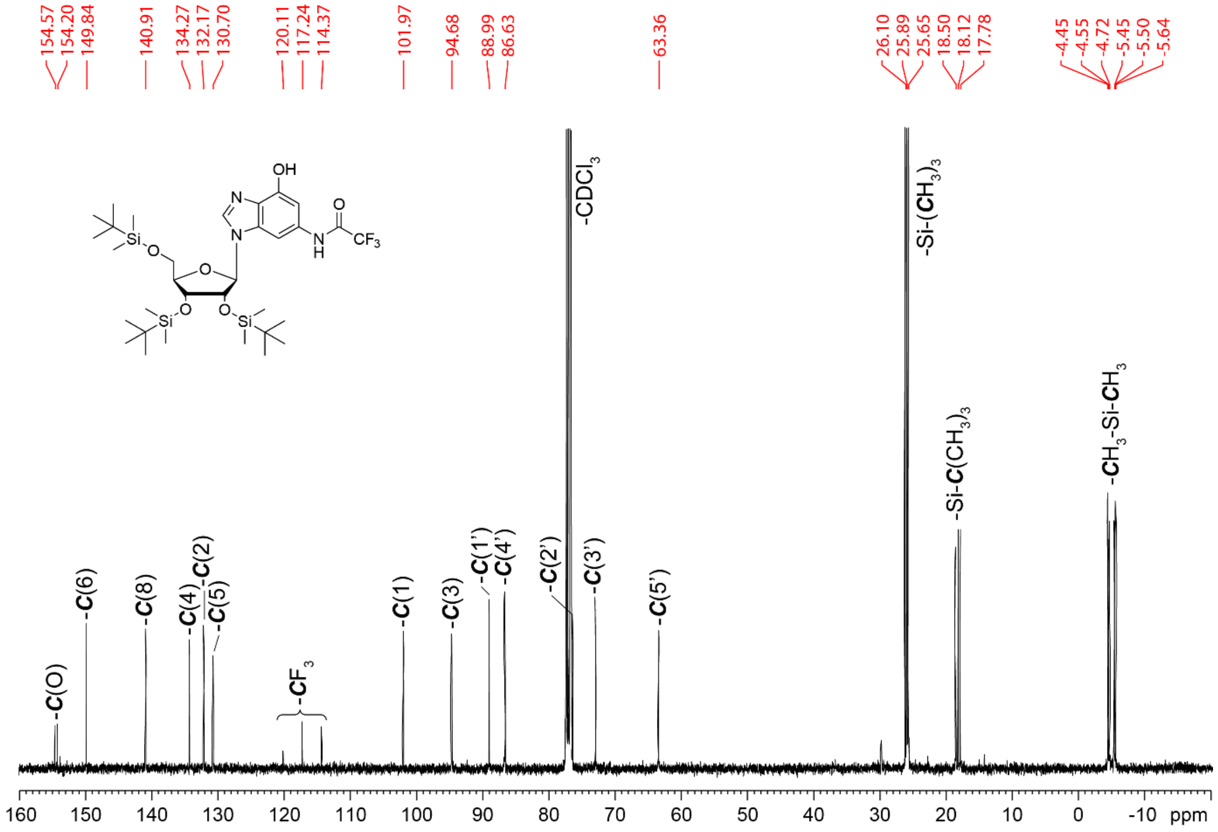

O^6-(*p*-Nitrophenylethyl)-N^2-trifluoroacetyl-1,3-dideazaguanosine (9)

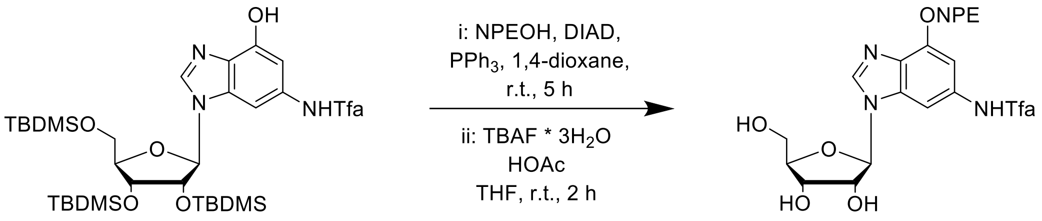

8 **9**

Step i: Compound **8** (3.20 g, 4.44 mmol), triphenylphosphine (PPh_3, 1.75 g, 6.67 mmol) and 2-(4-nitrophenyl)ethanol (NPEOH, 891 mg, 5.33 mmol) were dissolved in dry 1,4-dioxane (24 mL) and stirred for 10 minutes. Then, diisopropyl azodicarboxylate (DIAD, 2.72 g, 2.64 mL, 13.44 mmol) was added over a syringe and the mixture was stirred at room temperature for 4.5 hours. After LC-MS showed full conversion, the solvent was removed under reduced pressure, and the oily residue was purified with silica gel chromatography using 0 to10% ethyl acetate in toluene to obtain the NPE-protected intermediate (TLC (toluene/ethyl acetate 8/2): R_f = 0.45).

Step ii: Subsequently, the NPE-protected intermediate (3.50 g, 4.03 mmol; obtained from step i) was dissolved in tetrahydrofuran (35 mL), and tetra-n-butylammonium fluoride trihydrate (TBAF * $3H_2O$, 5.07 g, 16.1 mmol), was added together with a few drops of acetic acid (five drops per mmol). The reaction mixture was stirred at room temperature for two hours. Afterwards, the solvent was removed and the crude product was purified by silica gel chromatography using a gradient of 0 to 10% methanol in dichloromethane to yield compound **9**.

Yield: 1.90 g of compound **9** as a white solid (89%)
TLC (dichloromethane/methanol, 9/1)**:** R_f = 0.58
HR-ESI-MS (m/z): $[M+H]^+$ calcd. 527.1384; found 527.1402

^{1}H-NMR (400 MHz, DMSO, 25 °C):
δ = 3.30 (2H, t, J=6.64 Hz, C***H₂***(NPE)); 3.54-3.71 (2H, m, ***H(a)*** & ***H(b)***-C(5')); 3.96 (1H, q, J=3.89 Hz, ***H***-C(4')); 4.09 (1H, q, J=4.49 Hz, ***H***-C(3')); 4.35 (1H, t, J=5.84 Hz, ***H***-C(2')); 4.53 (2H, t, J=6.64 Hz, O-C***H₂***(NPE)); 5.04 (1H, t, J=5.64 Hz, O***H***(5')); 5.26 (1H, d, J=5.00 Hz, O***H***(3')); 5.49 (1H, d, J=6.44 Hz, O***H***(2')); 5.77 (1H, d, J=6.12 Hz, ***H***-C(1')); 7.07 (1H, d, J=1.52 Hz, ***H***-C(1)); 7.68 (3H, m, (***H***-C(3)&-***H***-C_{ortho}(NPE)); 8.19 (2H, d, J=2.92 Hz, ***H***-C_{meta}(NPE)); 8.38 (1H, s, ***H***-C(8));11.22 (1H, s, N***H***) ppm.

^{13}C-NMR (100 MHz, DMSO, 25 °C):
δ = 35.21 (***C***H_2(NPE)); 61.75 (***C***(5')); 69.05 (O-***C***H_2(NPE)); 70.69 (***C***(3')); 74.03 (***C***(2')); 85.88 (***C***(4')); 88.85 (***C***(1')); 97.45 (***C***(3)); 100.84 (***C***(1)); 114.89-120.63 (***C***F_3); 123.85 (***C***$_{meta}$(NPE)), 130.81 (***C***$_{ortho}$(NPE)); 132.08 (***C***(5)); 132.73 (***C***(2)); 135.00 (***C***(4)); 141.88 (***C***(8));146.72 (***C***$_{para}$(NPE)); 147.34 ***C***$_{ipso}$(NPE)); 150.06 (***C***(6)); 154.81 (***C***O) ppm.

^{1}H-NMR (400 MHz, CDCl$_3$, 25 °C):

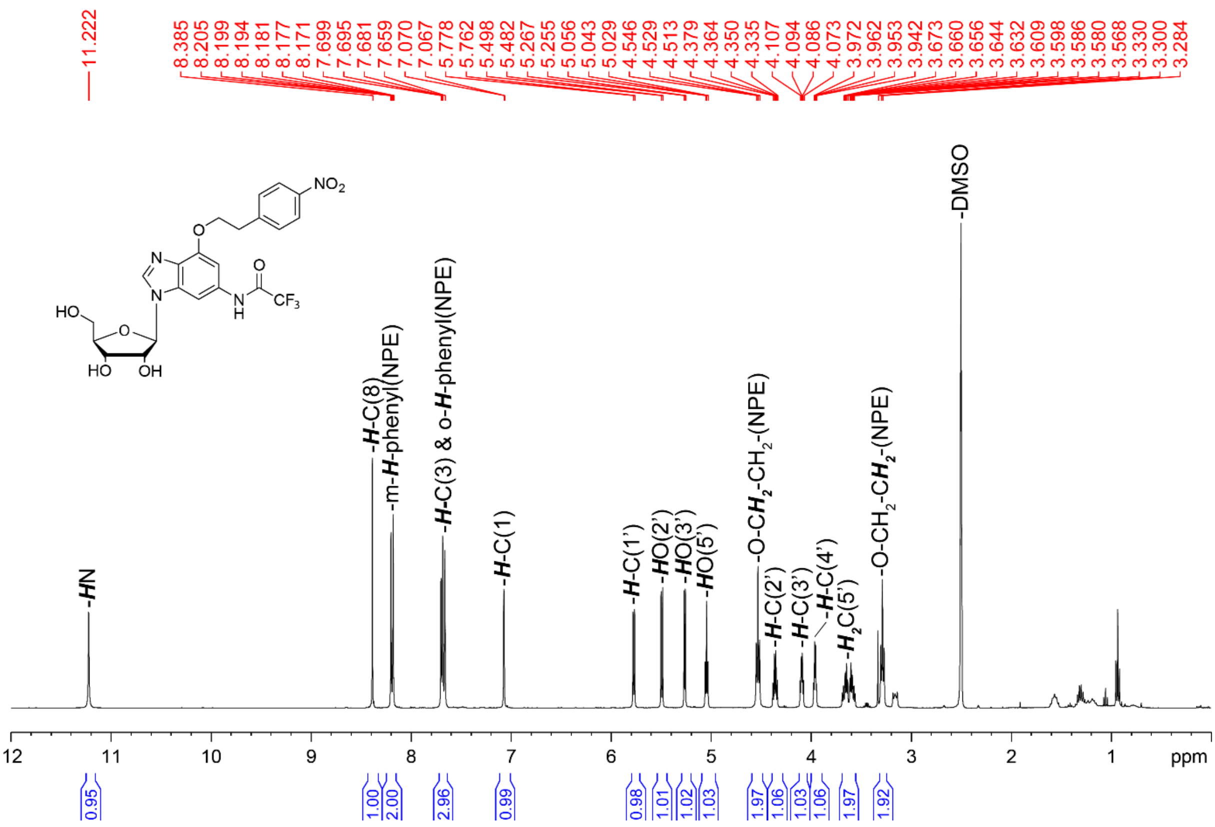

^{13}C-NMR (100 MHz, CDCl$_3$, 25 °C):

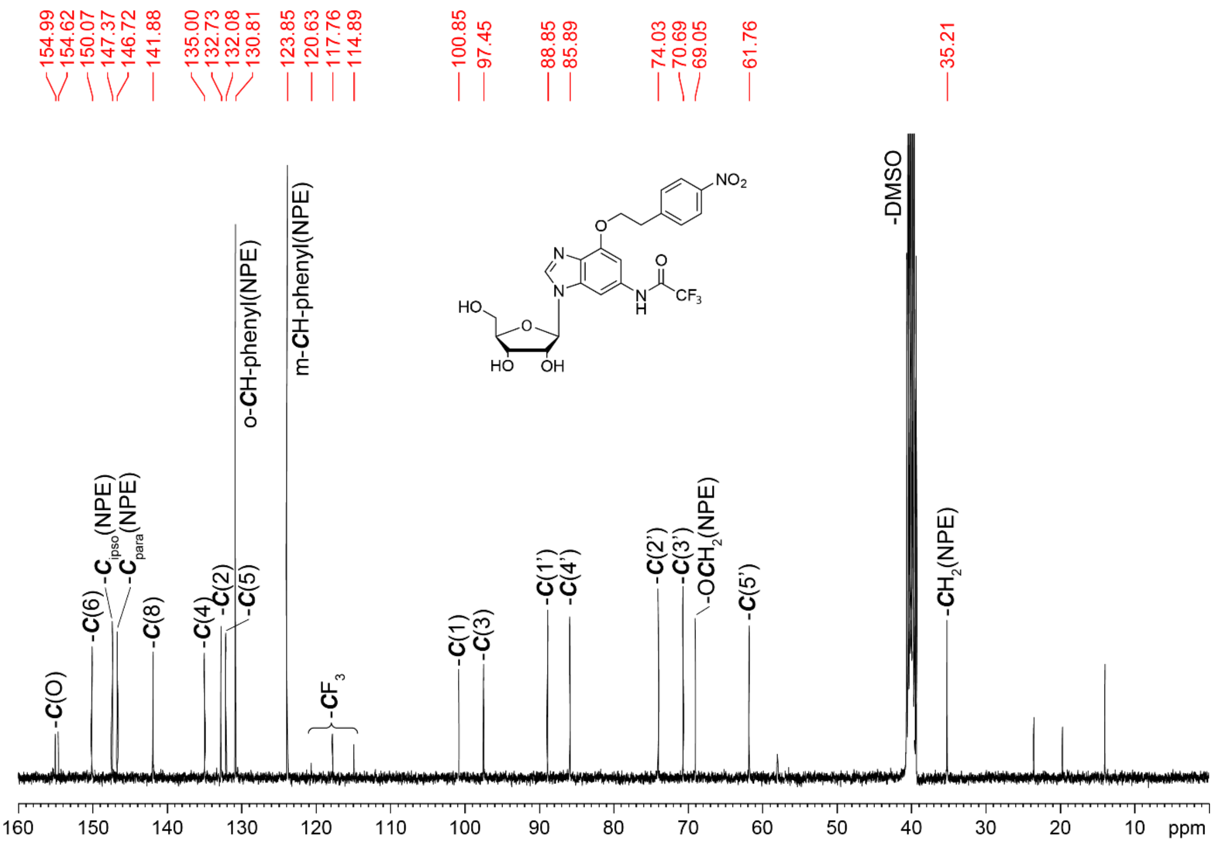

5'-*O*-(4,4'-Dimethoxytrityl)-*O*^{6}-(*p*-nitrophenylethyl)-*N*^{2}-trifluoroacetyl-1,3-dideaza-guanosine (10)

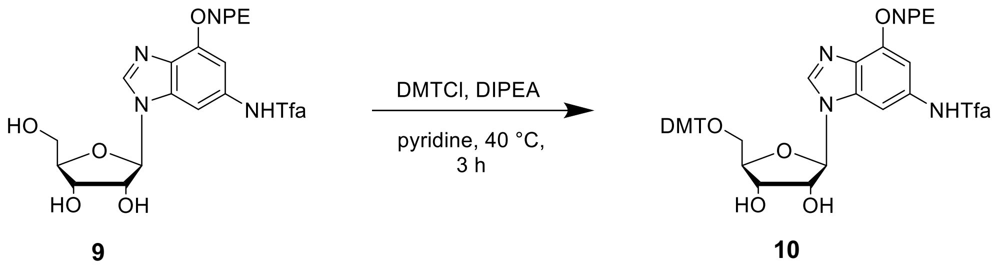

Compound **9** (1.80 g, 3.42 mmol), was coevaporated three times with pyridine, dried under high vacuum for half an hour and dissolved in dry pyridine (35 mL) at 40 °C. Then, *N,N*-diisopropylethylendiamine (DIPEA, 1.75 mL, 10.0 mmol) was added, followed by 4,4'-dimethoxytrityl chloride (DMTCl, 1.70 g, 5.01 mmol), which was added in three portions over the course of one hour. The mixture was allowed to stir for 3 hours at 40 °C. After TLC showed high conversion, the solvent and all volatiles were evaporated, the oily residue was dissolved in chloroform/isopropanol (3:1) and transferred into a separatory funnel. The organic layer was extracted with saturated sodium bicarbonate solution, brine, dried over sodium sulfate and evaporated to dryness. The crude product was purified with silica gel chromatography using a gradient of 50 to 100 % ethyl acetate in cyclohexane to yield compound **10**.

Yield: 2.08 g of compound 1**0** as a slightly beige foam (75%)
TLC (dichloromethane/methanol, 98/2)**:** R_f = 0.58
HR-ESI-MS (m/z): $[M+H]^+$ calcd. 829.2691; found 829.2675

^{1}H-NMR (400 MHz, DMSO, 25 °C):
δ = 3.20 (2H, m, ***H(a)*** & ***H(b)***-C(5')); 3.28 (2H, t, J=6.52 Hz, C***H₂***(NPE)); 3.69 (6H, s, 2xC***H₃***(DMT)); 4.11 (2H, m, ***H***-C(4') & ***H***-C(3')); 4.52 (3H, t, J=6.64 Hz, O-C***H₂***(NPE) & ***H***-C(2')); 5.28 (1H, d, J=5.88 Hz, O***H***(3')); 5.66 (1H, d, J=5.88 Hz, O***H***(2')); 5.85 (1H, d, J=4.84 Hz, ***H***-C(1')); 6.77-7.31 (14H, m, ***H***-C(DMT) & ***H***-C(1)); 7.66 (2H, d, J=1.26 Hz, ***H***-C_{ortho}(NPE)); 7.76 (1H, d, J= 8.68 Hz, ***H***-C(3));8.18 (2H, d, J=2.92 Hz, ***H***-C_{meta}(NPE)); 8.29 (1H, s, ***H***-C(8));11.22 (1H, s, N***H***) ppm.

^{13}C-NMR (100 MHz, DMSO, 25 °C):
δ = 35.21 (***C***H_2(NPE)); 55.40 (2x***C***H_3 (DMT)); 64.15 (***C***(5')); 69.05 (O-***C***H_2(NPE)); 70.80 (***C***(3')); 73.76 (***C***(2')); 83.62 (***C***(4')); 85.93 (***C***$_{quart.}$(DMT)); 89.32 (***C***(1')); 97.24 (***C***(3)); 100.63 (***C***(1)); 113.55 (***C***(DMT)); 114.84-117.71 (***C***F_3); 123.84 (***C***$_{meta}$(NPE)), 127.04-130.14 (***C***(DMT)); 130.81 (***C***$_{ortho}$(NPE)); 131.99 (***C***(5)); 133.05 (***C***(2)); 135.18 (***C***(4)); 135.90 (***C***$_{quart.}$(DMT)); 141.17 (***C***(8)); 145.31 (***C***$_{quart.}$(DMT)); 146.71 (***C***$_{para}$(NPE)); 147.34 ***C***$_{ipso}$(NPE)); 150.09 (***C***(6)); 154.70 (***C***O); 158.46 (***C***$_{quart.}$(DMT)) ppm.

^{1}H-NMR (400 MHz, $CDCl_3$, 25 °C):

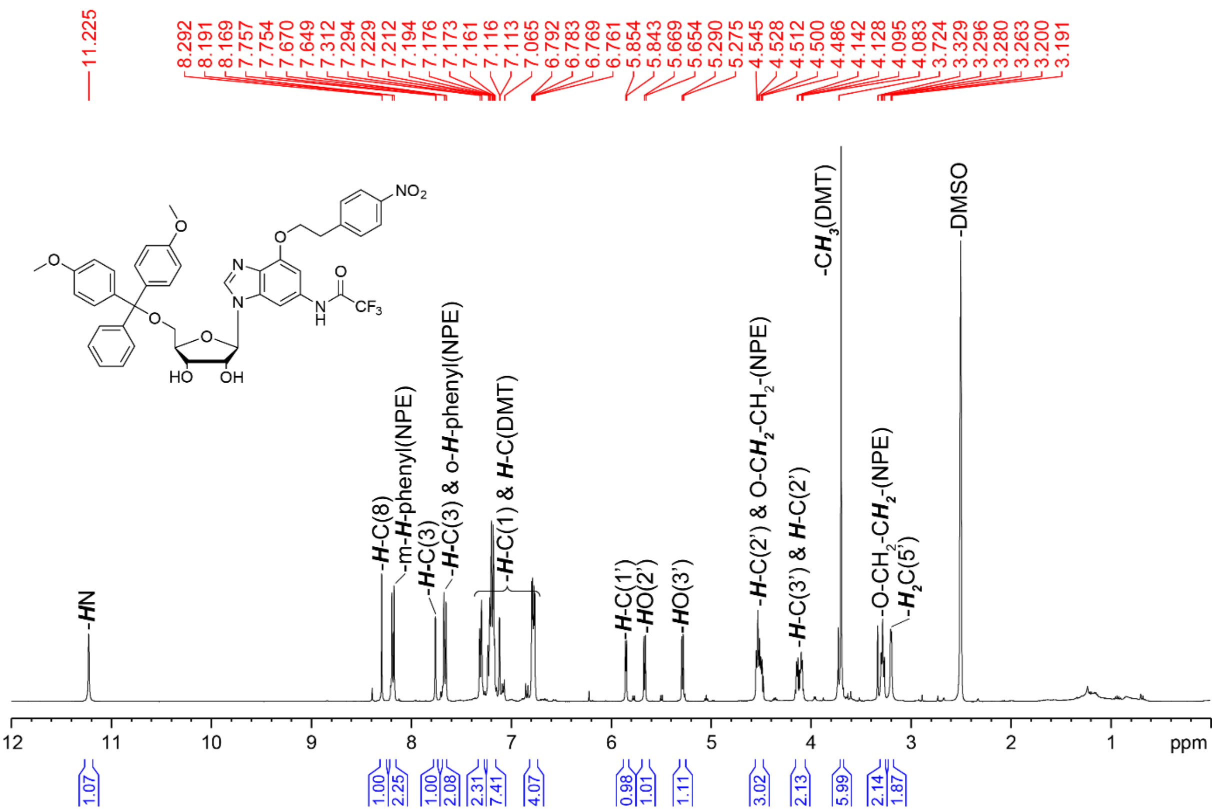

^{13}C-NMR (100 MHz, $CDCl_3$, 25 °C):

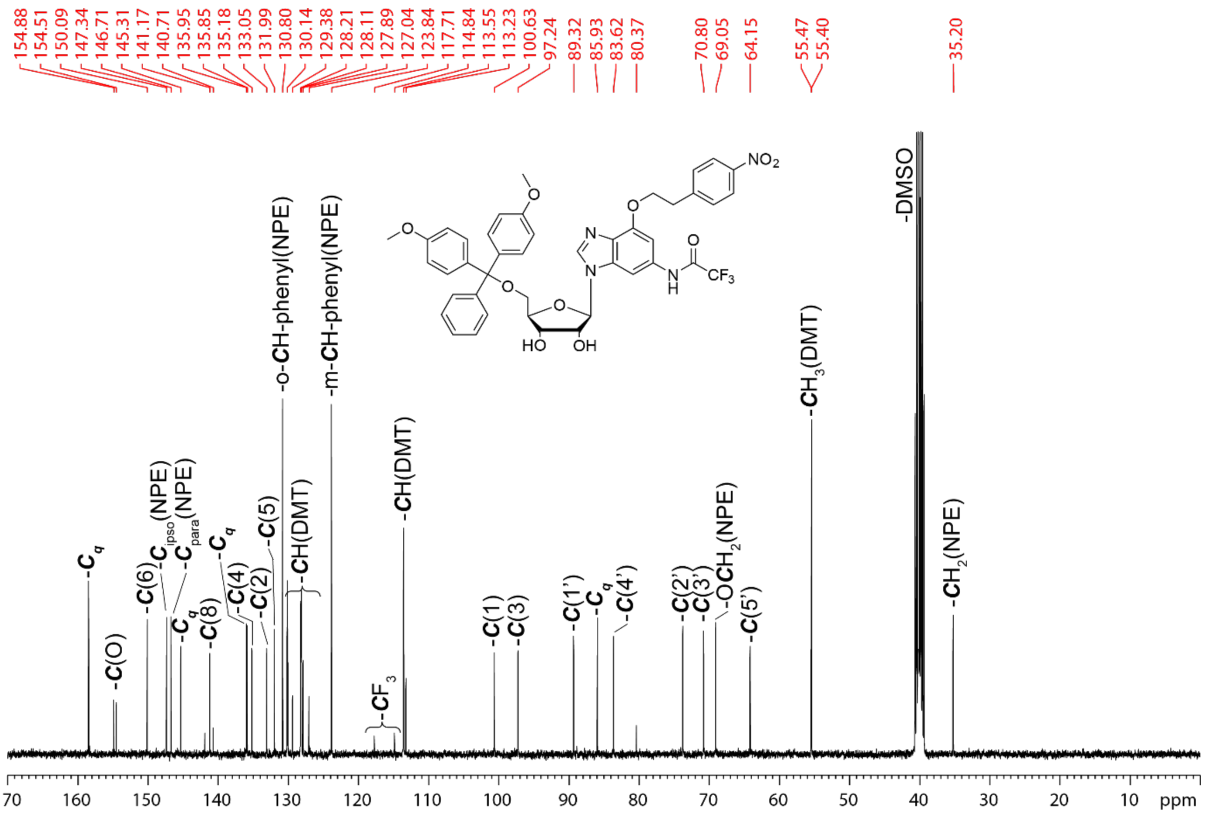

5'-*O*-(4,4'-Dimethoxytrityl)-*O*[6]-(*p*-nitrophenylethyl)-2'-*O*-(*tert*-butyldimethylsilyl)-*N*[2]-trifluoroacetyl-1,3-dideazaguanosine (11)

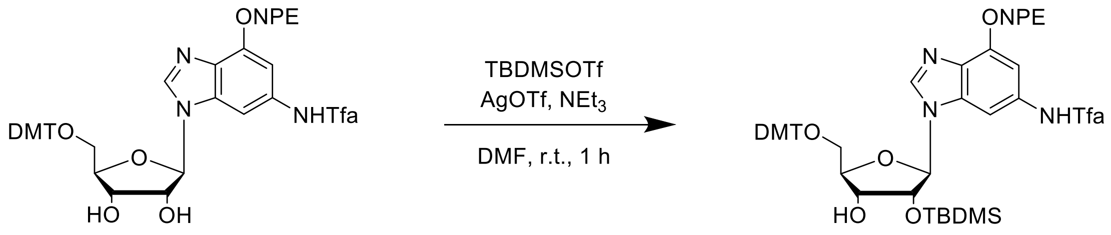

10 **11**

Compound **10** (0.95 g, 1.15 mmol) and silver trifluoromethanesulfonate (AgOTf, 353 mg, 1.43 mmol) were dissolved in *N,N*-dimethylformamide (12.5 mL) under light exclusion. Then, triethylamine (NEt_3, 240 µL, 1.72 mmol) was added, followed by *tert*-butyldimethylsilyl trifluromethanesulfonate (TBDMSOTf, 263 µL, 1.15 mmol). The reaction mixture was stirred for 1 hour at room temperature under light exclusion, predominantly, forming the 3'-O (!) protected derivative. After TLC showed almost full conversion, the solvent and all volatiles were evaporated, the oily residue was dissolved in dichloromethane and transferred into a separatory funnel. The organic layer was extracted with saturated sodium bicarbonate solution, brine, dried over sodium sulfate and evaporated to dryness. The crude product was taken up for 2'/3'-O equilibration using dichloromethane/methanol/ triethylamine (8/1/1) and stirred overnight to provide a one-to-one mixture of the 2'-O and 3'-O protected isomers. The slightly faster migrating 2'-O-TBDMS isomer was isolated by silica gel column chromatography using a gradient of 0 to 20 % ethyl acetate in toluene. All fractions containing the mixture of 2'-O and 3'-O isomers or the 3' isomer were again collected for equilibration and separated by chromatography to isolate the desired 2'-O compound **11**.

Yield: 487 mg of compound **11** as a slightly green foam (45%)
TLC (toluene/ethyl acetate, 7/3)**:** R_f = 0.48
HR-ESI-MS (m/z): $[M+H]^+$ calcd. 943.3556; found 943.3542

^{1}H-NMR (400 MHz, DMSO, 25 °C):
δ = -0.33 (3H, s, Si-C***H₃***); -0.13 (3H, s, Si-C***H₃***); 0.81 (9H, s, Si-C(C***H₃***)₃); 2.76 (1H, s, O***H***(3')); 3.31 (3H, m, ***H(a)***-C(5') & C***H₂***(NPE)); 3.70 (1H, m, ***H(b)***-C(5')); 3.76 (6H, s, 2xC***H₃***(DMT)); 4.20 (1H, d, J=5.00 Hz, ***H***-C(3')); 4.29 (1H, m, ***H***-C(4')); 4.50 (2H, t, J=6.72 Hz, O-C***H₂***(NPE)); 4.76 (1H, t, J=6.28 Hz, (***H***-C(2')); 5.81 (1H, d, J=7.32 Hz, ***H***-C(1')); 6.77-7.53 (17H, m, ***H***-C(DMT) & ***H***-C_{ortho}(NPE)) & ***H***-C(1) & ***H***-N); 7.64 (1H, s, ***H***-C(3)); 8.02 (1H, s, ***H***-C(8)); 8.15 (2H, d, J=2.92 Hz, ***H***-C_{meta}(NPE)) ppm.

^{13}C-NMR (100 MHz, $CDCl_3$, 25 °C):
δ = -5.37 (***C***H₃-Si-***C***H₃); 17.84 (Si-***C***(CH₃)₃); 25.51 (Si-C(***C***H₃)₃; 35.51 (***C***H₂(NPE)); 55.26 (2x***C***H₃ (DMT)); 63.68 (***C***(5')); 68.89 (O-***C***H₂(NPE)); 71.43 (***C***(3')); 74.87 (***C***(2')); 84.51 (***C***(4')); 86.63 (***C***$_{quart.}$(DMT)); 89.06 (***C***(1')); 97.38 (***C***(3)); 99.85 (***C***(1)); 113.39 (***C***(DMT)); 113.35-116.65 (***C***F₃); 123.75 (***C***$_{ortho}$(NPE)), 127.31-129.93 (***C***(DMT)); 130.00 (***C***$_{metha}$(NPE)); 131.86 (***C***(2)); 132.77 (***C***(5)); 133.88 (***C***(4)); 135.21 (***C***$_{quart.}$(DMT)); 135.58 (***C***$_{quart.}$(DMT)); 141.38 (***C***(8)); 144.47 (***C***$_{quart.}$(DMT)); 145.99 (***C***$_{ipso}$(NPE)); 146.85 ***C***$_{para}$(NPE)); 150.61 (***C***(6)); 154.70 (***C***O); 158.83 (***C***$_{quart.}$(DMT)) ppm.

^{1}H-NMR (400 MHz, CDCl$_3$, 25 °C):

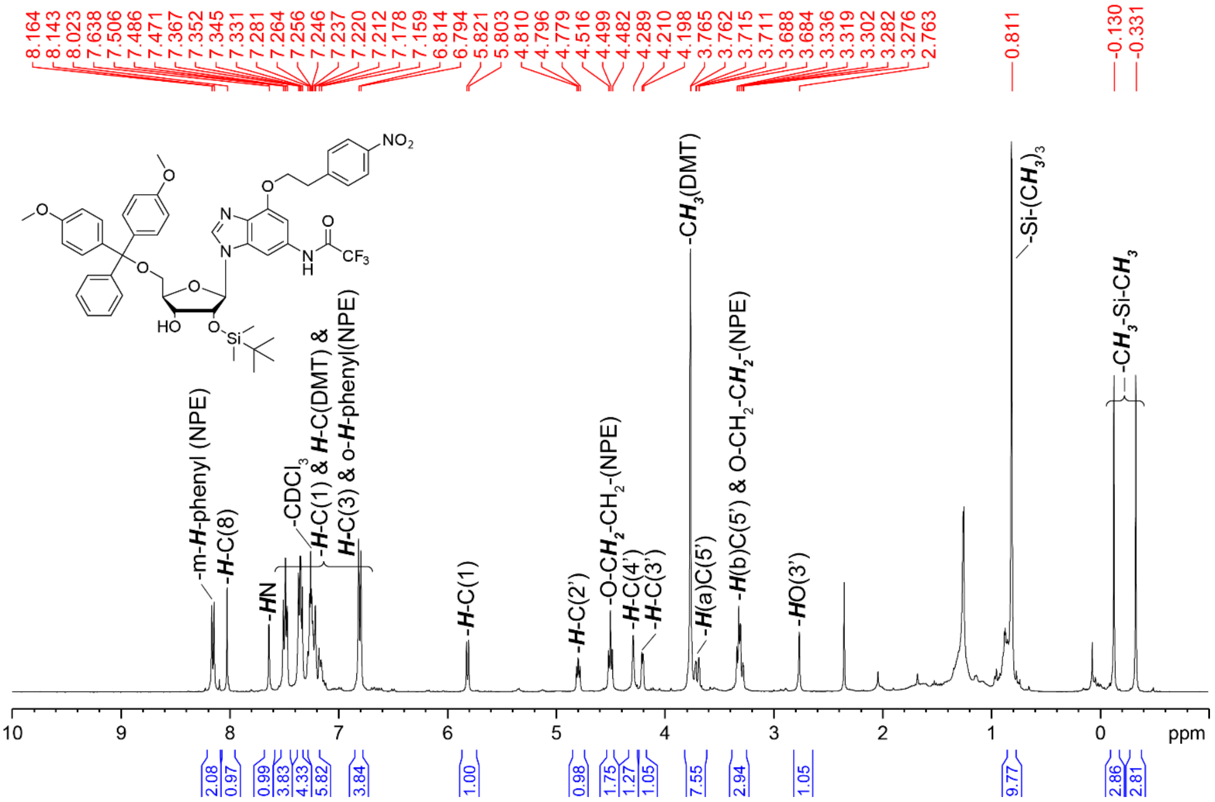

^{13}C-NMR (100 MHz, CDCl$_3$, 25 °C):

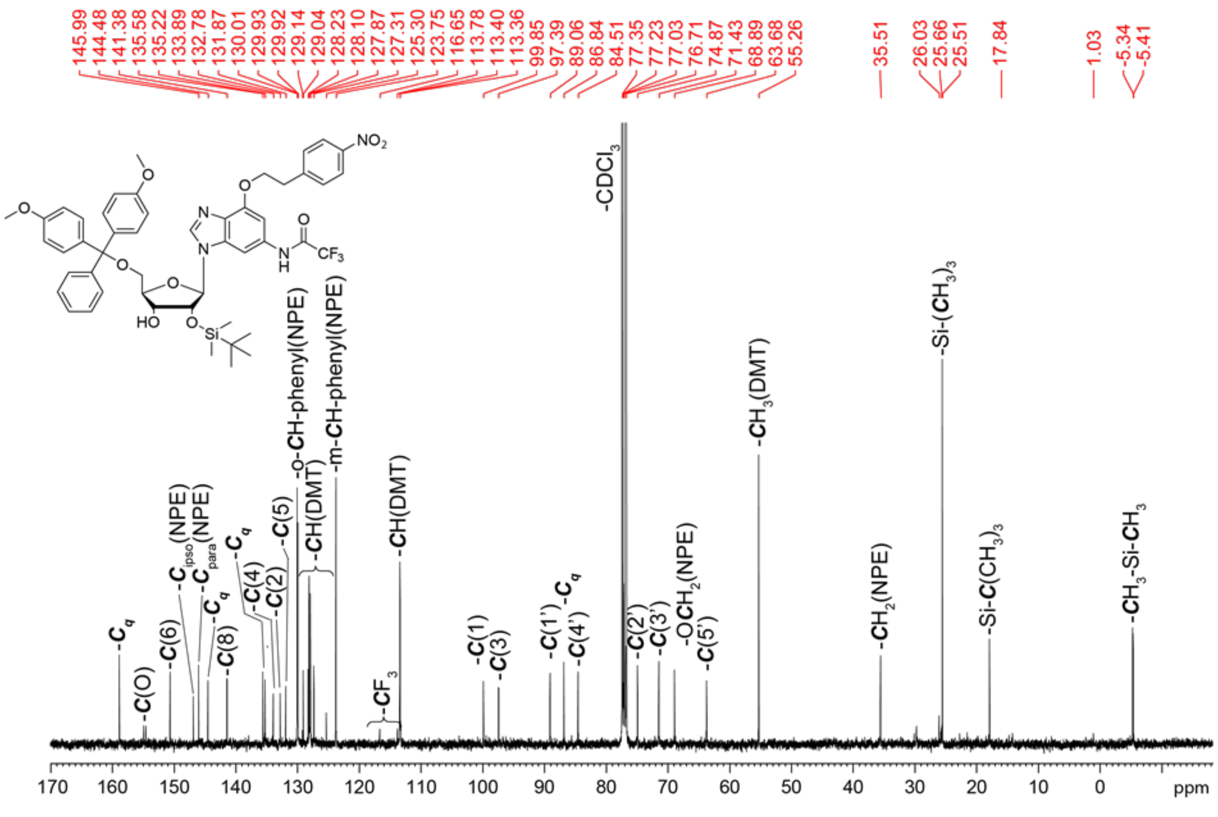

^{1}H,^{1}H-COSY NMR (400 MHz, $CDCl_3$, 25 °C):

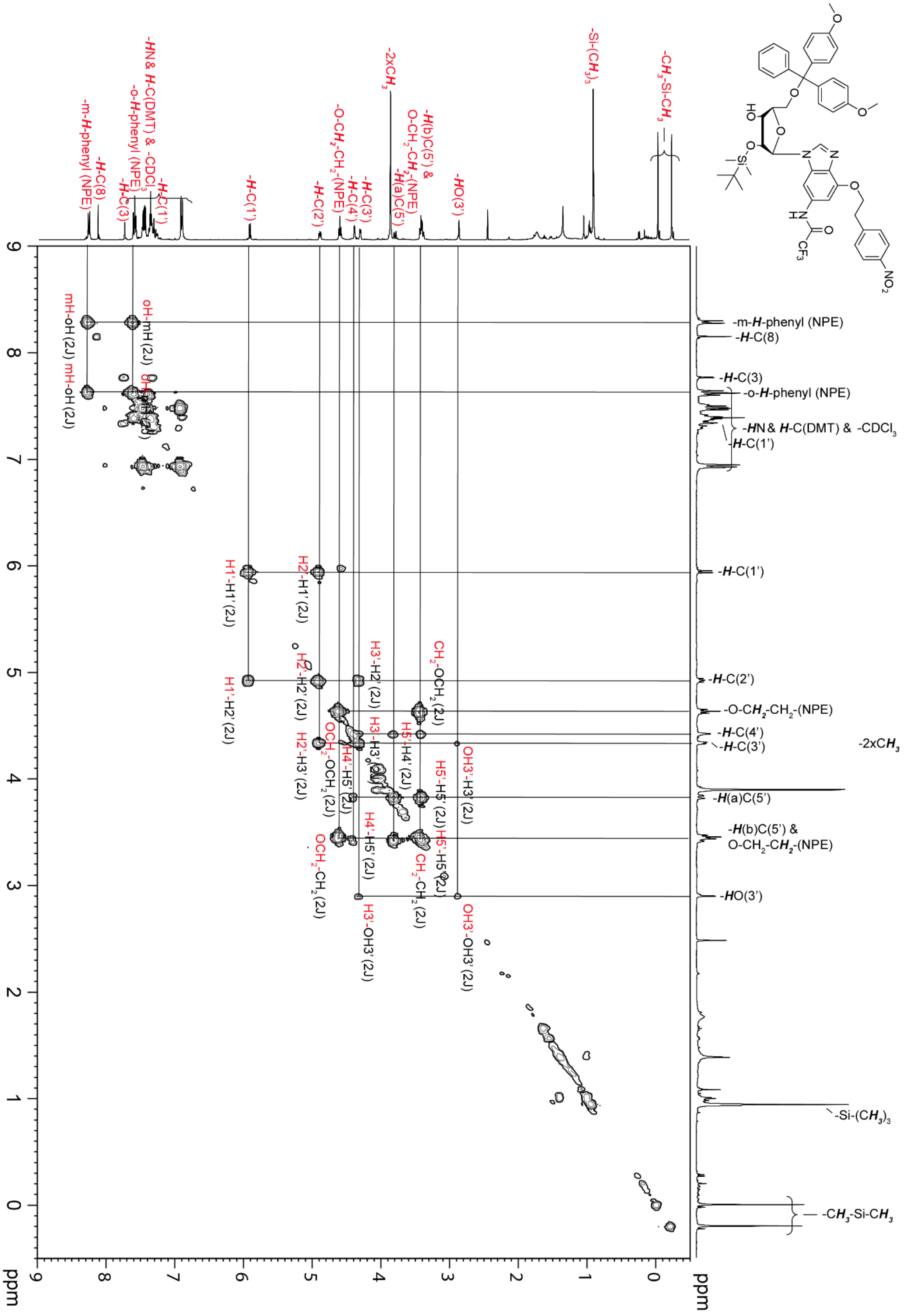

$^{1}H,^{13}C$-HMBC NMR (400 MHz, $CDCl_3$, 25 °C):

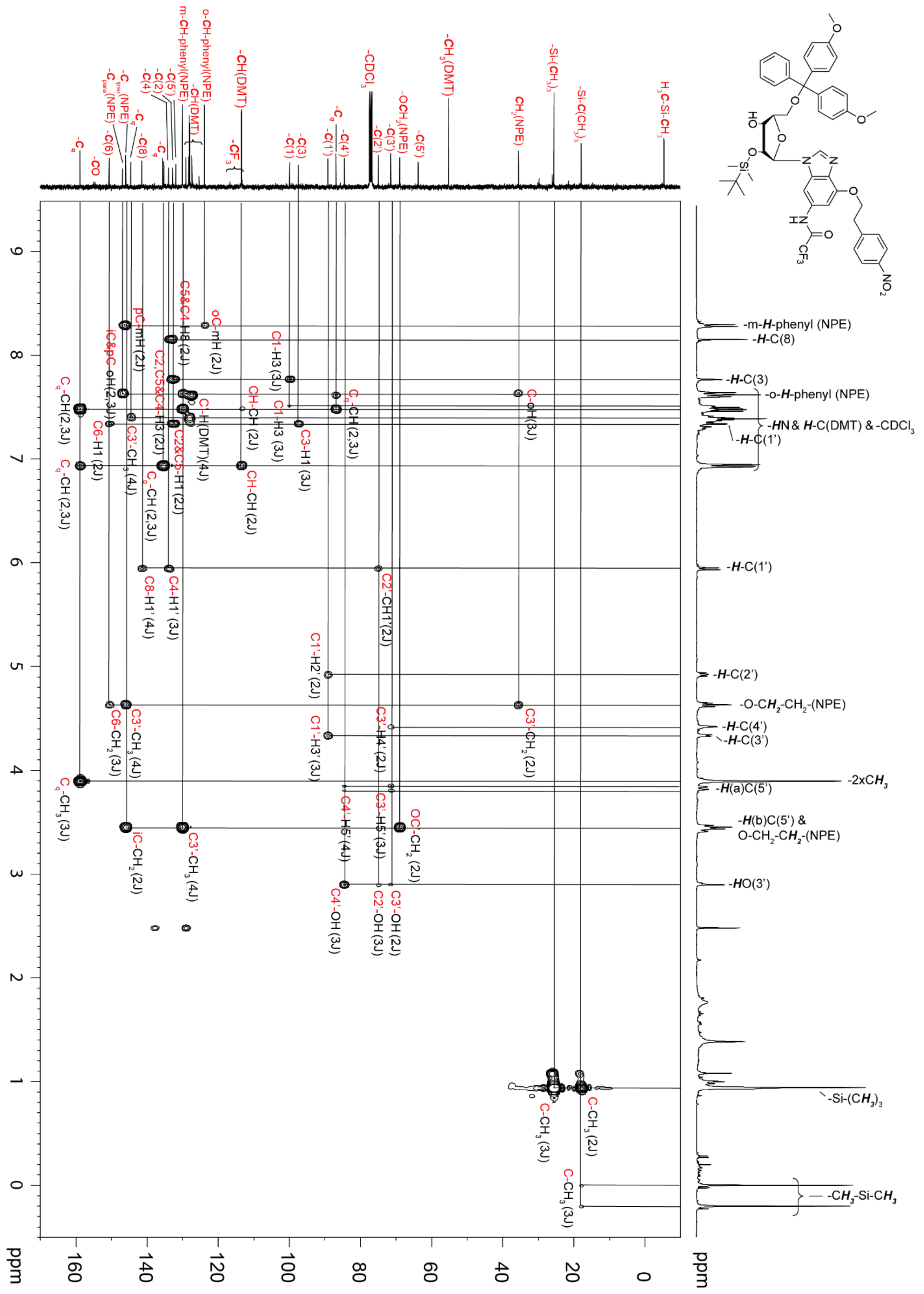

5'-*O*-(4,4'-Dimethoxytrityl)-*O*^{6}-(*p*-Nitrophenylethyl)-2'-*O*-(*tert*-butyldimethylsilyl)-*N*^{2}-trifluoroacetyl-1,3-dideazaguanosine 3'-*O*-(2-cyanoethyl)-*N,N*-diisopropylphosphoramidite (12)

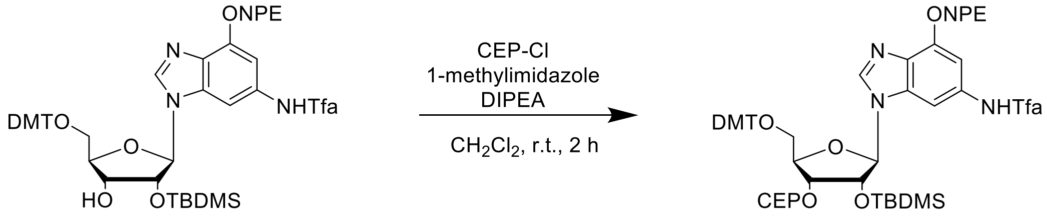

11 **12**

Compound **11** (300 mg, 318 µmol) was coevaporated three times (dry pyridine, toluene, dichloromethane) and dried under high vacuum for 20 min. The dry compound was charged with argon, dissolved in dichloromethane (3 mL) and molecular sieve was added. Subsequently, N,N-diisopropylethylamine (DIPEA, 138 µl, 795 µmol), 1-methylimidazole (13 µl, 159 µmol) and 2-cyanoethyl-*N,N*-diisopropylchlorophosphoramidite (CEP-Cl, 92 µl, 414 µmol) were introduced. The reaction mixture was stirred until TLC showed full conversion of the starting material. The solvent and all volatiles were evaporated and the crude product was purified by silica gel column chromatography using a gradient of 30 to 60 % ethyl acetate in cyclohexane (containing 1 % triethylamine) to yield compound **12**. Further, the so-purified solid phosphoramidite **12** was dissolved in dichloromethane (approximately 1 mL) and precipitated from ice cold n-pentane (20 mL) under vigorous stirring. This was repeated three times to obtain the phosphoramidite **12** without any phosphonate byproduct (that accumulated from hydrolysis of CEP-Cl).

Yield: 210 mg of compound **12** as a white foam (61 %)
TLC (cyclohexane/ethyl acetate, 6/4): R_f = 0.62
HR-ESI-MS (m/z): $[M+H]^+$ calcd. 1143.4634; found 1143.4614

^{1}H-NMR (400 MHz, $CDCl_3$, 25 °C, 2 diastereomers**):**
δ = -0.40-0.36 (3H, s, Si-C$\boldsymbol{H_3}$); -0.17-0.11 (3H, s, Si-C$\boldsymbol{H_3}$); 0.73 (9H, m, Si-C$\boldsymbol{H_3}$); 0.74 (9H, s, Si-C(C$\boldsymbol{H_3}$)$_3$); 0.98-1.29 (12H, m, (((C$\boldsymbol{H_3}$)$_2$-CH)$_2$-N)); 2.20-2.75 (2H, m, (OCH$_2$C$\boldsymbol{H_2}$CN)); 3.24 (1H, m, ***H(a)***-C(5')); 3.33 (2H, m, C$\boldsymbol{H_2}$(NPE)); 3.45-3.66 (4H, m, (((CH$_3$)$_2$-C***H***)$_2$-N) & ***H(b)***-C(5') & C$\boldsymbol{H_2}$(b)-O-P); 3.76 (6H, s, 2xC$\boldsymbol{H_3}$(DMT)); 3.88-4.11 (1H, m, (C$\boldsymbol{H_2}$(a)-O-P)); 4.26 (2H, m, (***H***-C(3') & ***H***-C(4')); 4.51 (2H, m, O-C$\boldsymbol{H_2}$(NPE)); 4.74 (1H, m, ***H***-C(2')); 5.76-5.92 (H, 2xd, J^{Dia1}=7.51 Hz, J^{Dia2}=7.90 Hz, ***H***-C(1')); 6.77-7.59 (17H, m, ***H***-C(DMT) & ***H***-C_{ortho}(NPE)) & ***H***-C(1) & ***H***-C(3)); 8.03 (1H, s, N***H***); 8.11-8.18 (3H, s, ***H***-C(8) & ***H***-C_{meta}(NPE)) ppm.

^{31}P-NMR (162 MHz, $CDCl_3$, 25 °C, 2 diastereomers**):**
δ = 151.50 & 148.31 ppm.

^{1}H-NMR (400 MHz, CDCl₃, 25 °C, 2 diastereomers**):**

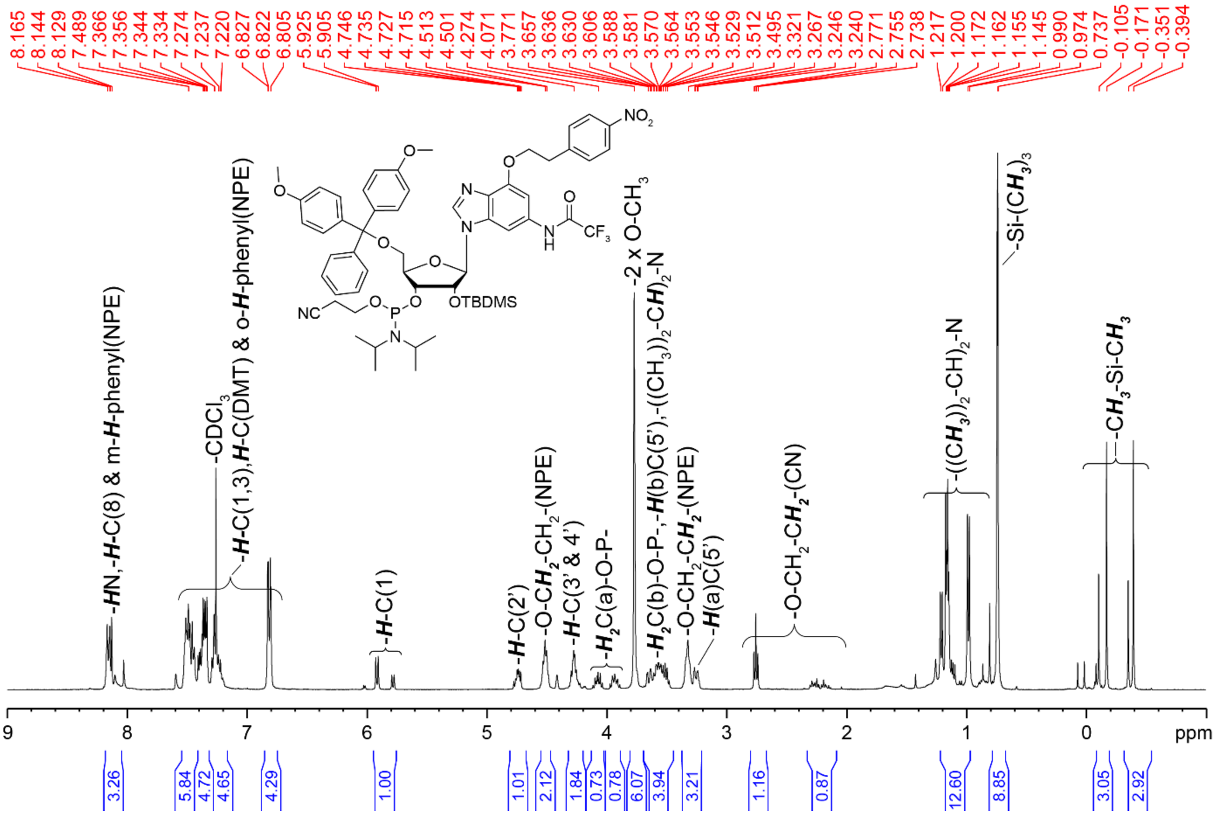

^{31}P-NMR (162 MHz, CDCl₃, 25 °C, 2 diastereomers**):**

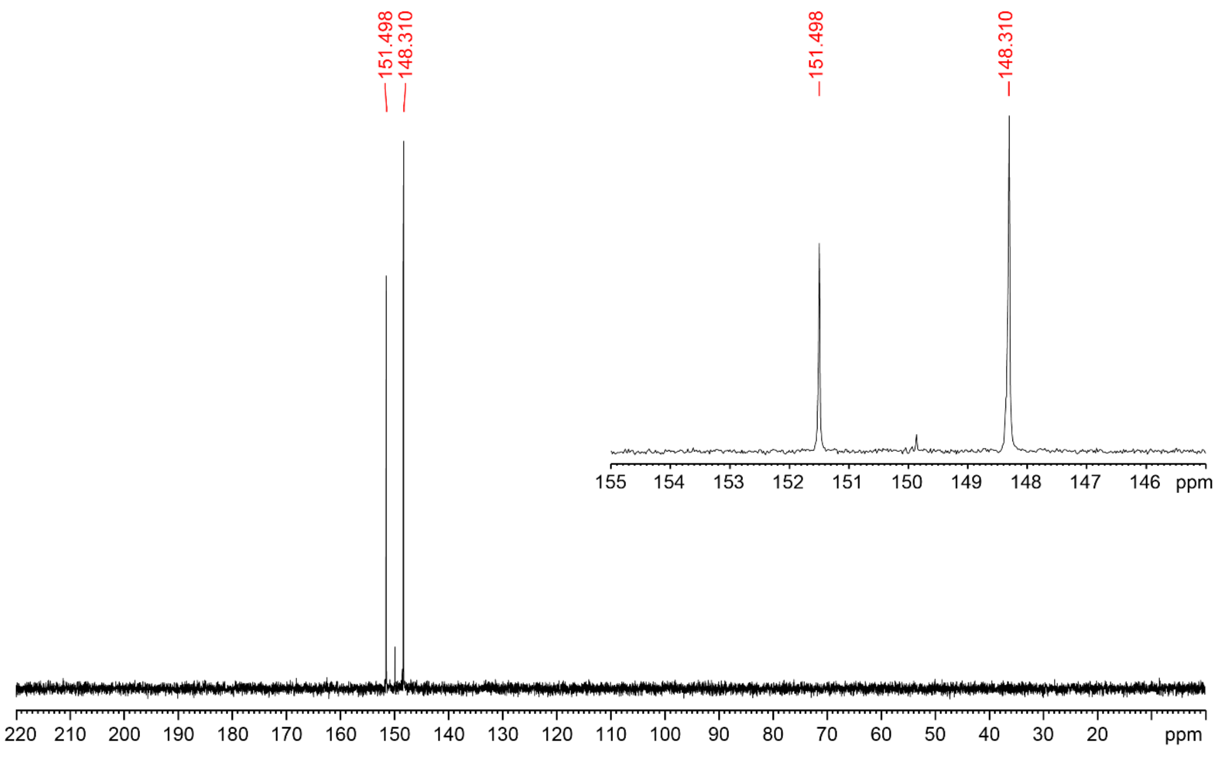

^{1}H,^{1}H-NMR (400 MHz, CDCl$_3$, 25 °C, 2 diastereomers**):**

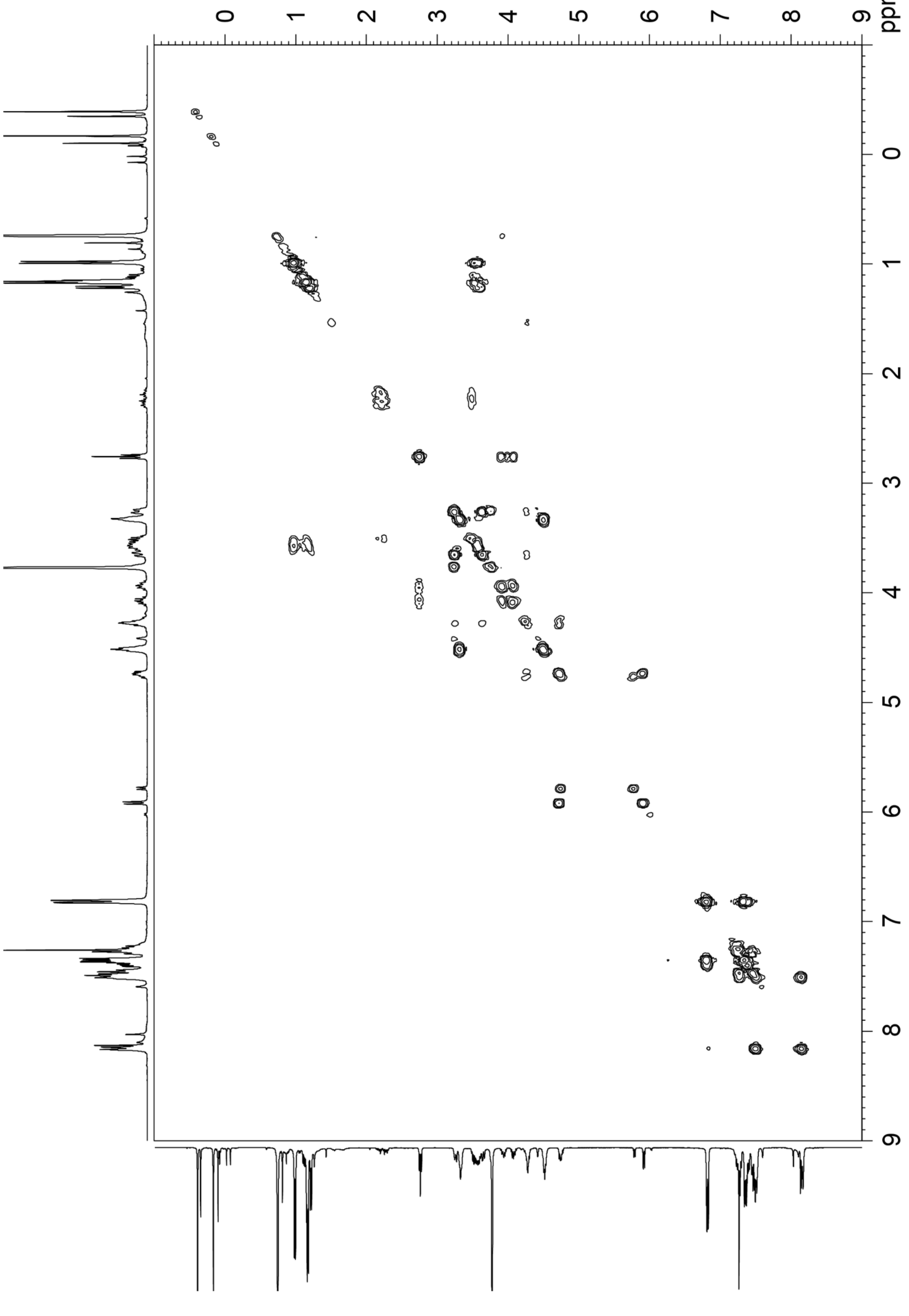

Supporting Table S1. Sequences and mass spectrometric analysis of RNAs used in this study.

Sequence (5' to 3')	nt	$m.w._{calc.}$ [amu]	$m.w._{found}$ [amu]
GGCAGAGGC (**I**)	9	2932.87	2932.59
CCGUCUCCG	9	2766.70	2766.34
GGCAc^1c^3GAGGC (**Ia**)	9	2930.91	2930.47
GGACCGGUCC (**II**)	10	3174.98	3174.48
GGACCGc^1c^3GUCC (**IIa**)	10	3173.02	3172.47
GAAGGGCAACCUUCG (**III**)	15	4813.99	4813.70
GAAGGGCAACCUUCG (**IIIa**)	15	4812.03	4811.68
GAAAGGCAACUUUCG	15	4798.98	4798.70
GGUCGACC	8	2524.59	2524.38
GGUCc^1c^3GACC	8	2522.63	2522.39
CCUGUGAGGGUCCUAAGCCCCUAAUUCAGAAGGGAAA	37	11901.26	11901.48
CCUGUGAGGGUCCUAAGCCCCUAAUUCAc^1c^3GAAGGGAAA (**IV**)	37	11899.30	11899.47
UUUUUAAUGAAGCCACAGG	19	6046.70	6046.42

Supporting Table S2. Thermodynamic parameters of c^{1}c^{3}G-modified RNA (and unmodified references) obtained by UV melting profile analysis (Extension of Table 1).

#	RNA sequence[a]	T_m [°C]	ΔG^0 [b] [kcal mol^{-1}]	ΔH^0 [b] [kcal mol^{-1}]	ΔS^0 [b] [cal mol^{-1} K^{-1}]
**I**	5'GGCA**G**AGGC<br>3'CCGUCUCCG	66.1	-17.0±0.4	-84.5±1.9	-226±5
**Ia**	5'GGCA**c^{1}c^{3}G**AGGC<br>3'CCGUCUCCG	44.1	-10.8±0.3	-68.2±3.6	-193±12
ref. (26)[c]	5'GGCA**c^{1}G**AGGC<br>3'CCGUCUCCG	50.9	-13.1±0.9	-79.9±7.7	-224±23
ref. (27)[d]	5'GGCA**c^{3}G**AGGC<br>3'CCGUCUCCG	62.8	-15.2±0.5	-74.8±4.7	-199±14
ref. (61)[e]	5'GGCA**A**AGGC<br>3'CCGUUUCCG	59.5	-15.4±0.1	-84.0±0.7	-230±2
**II**	5'GGACCG**G**UCC	73.4	-21.3±0.1	-104.5 ±0.6	-279±2
**IIa**	5'GGACCG**c^{1}c^{3}G**UCC	36.1	-9.4±0.3	-74.1±2.5	-217±7
ref. (61)[e]	5'GGAUCG**A**UCC	59.7	-15.8±0.1	-87.0±0.3	-239±1
**III**	5' GAA**G**G-GCAA-CCUUCG	70.9	-6.9±0.5	-54.1±3.5	-159±10
**IIIa**	5'GAA**c^{1}c^{3}G**G-GCAA-CCUUCG	37.1	-1.4±0.1	-32.4±4.7	-105±15
ref. (26)[c]	5'GAA**c^{1}G**G-GCAA-CCUUCG	44.8	-2.8±0.4	-48.5±3.2	-153±9
ref. (27)[d]	5'GAA**c^{3}G**G-GCAA-CCUUCG	64.2	-6.2±0.2	-53.5±1.6	-159±5
	5'GAA**A**G-GCAA-CUUUCG	57.7	-4.9±0.3	-51.8±3.3	-157±10

[a] Buffer: 10 mM Na_2HPO_4, 150 mM NaCl, pH 7.0. T_m values are listed at a concentration of 12 µM RNA (calculated from ln c versus 1/T plots). The estimated errors of UV-spectroscopically determined T_m values are ±0.2 °C. ΔH and ΔS values were obtained by van't Hoff analysis according to refs (39,40). Errors for ΔH and ΔS, arising from noninfinite cooperativity of two-state transitions and from the assumption of a temperature-independent enthalpy, are typically 10–15%. Additional error is introduced when free energies are extrapolated far from melting transitions; errors for ΔG are typically 3–5%. [b] at 298 K. [c] Bereiter, R.; Renard, E.; Breuker, K.; Kreutz, C.; Ennifar, E.; Micura, R. 1-Deazaguanosine-Modified RNA: The Missing Piece for Functional RNA Atomic Mutagenesis. *J. Am. Chem. Soc.* **2022**, *144* (23), 10344–10352. [d] Bereiter, R.; Himmelstoß, M.; Renard, E.; Mairhofer, E.; Egger, M.; Breuker, K.; Kreutz, C.; Ennifar, E.; Micura, R. Impact of 3-Deazapurine Nucleobases on RNA Properties. *Nucleic Acids Res.* **2021**, *49* (8), 4281-4293. [e] Mair, S.; Rázková, A.; Brillet, K.; Gasser, C.; Ennifar, E.; Micura, R. Tailored Watson–Crick Pairing Partners for Xanthosine in RNA. *Chem. A Eur. J.* **2025**, *31* (42), e202501860. Measurements were performed in three independent experiments. Mean values ± s.e.m. are listed.

Supporting Table S3. pH dependent melting point temperatures of c^{1}c^{3}G-modified RNA (and unmodified references) obtained by UV melting profile analysis for Figure 4.

#	RNA sequence[a]	pH 5.0 T_m [°C]	pH 6.0 T_m [°C]	pH 7.0 T_m [°C]
**II**	5’GGACCG**G**UCC	71.15	72.83	73.49
		70.64	73.17	73.60
		70.96	73.23	73.23
**IIa**	5’GGACCG**c^{1}c^{3}G**UCC	42.80	39.86	35.97
		42.29	39.86	36.30
		42.35	39.84	36.30
**III**	5’ GAA**G**G-GCAA-CCUUCG	69.74	70.84	71.10
		69.65	70.75	71.09
		69.65	70.75	71.09
**IIIa**	5’GAA**c^{1}c^{3}G**G-GCAA-CCUUCG	43.15	42.13	37.26
		43.20	42.01	37.09
		43.11	41.92	37.09

[a] Buffer: Melting points used for the palindromic as well as for the hairpin pH dependence measured at 10 mM Na_2HPO_4, 150 mM NaCl, pH 5.0, 6.0, 7.0. T_m values are listed at a concentration of 12 µM RNA (calculated from ln c versus 1/T plots). Measurements were performed in triplicate (see the three entries per RNA above).

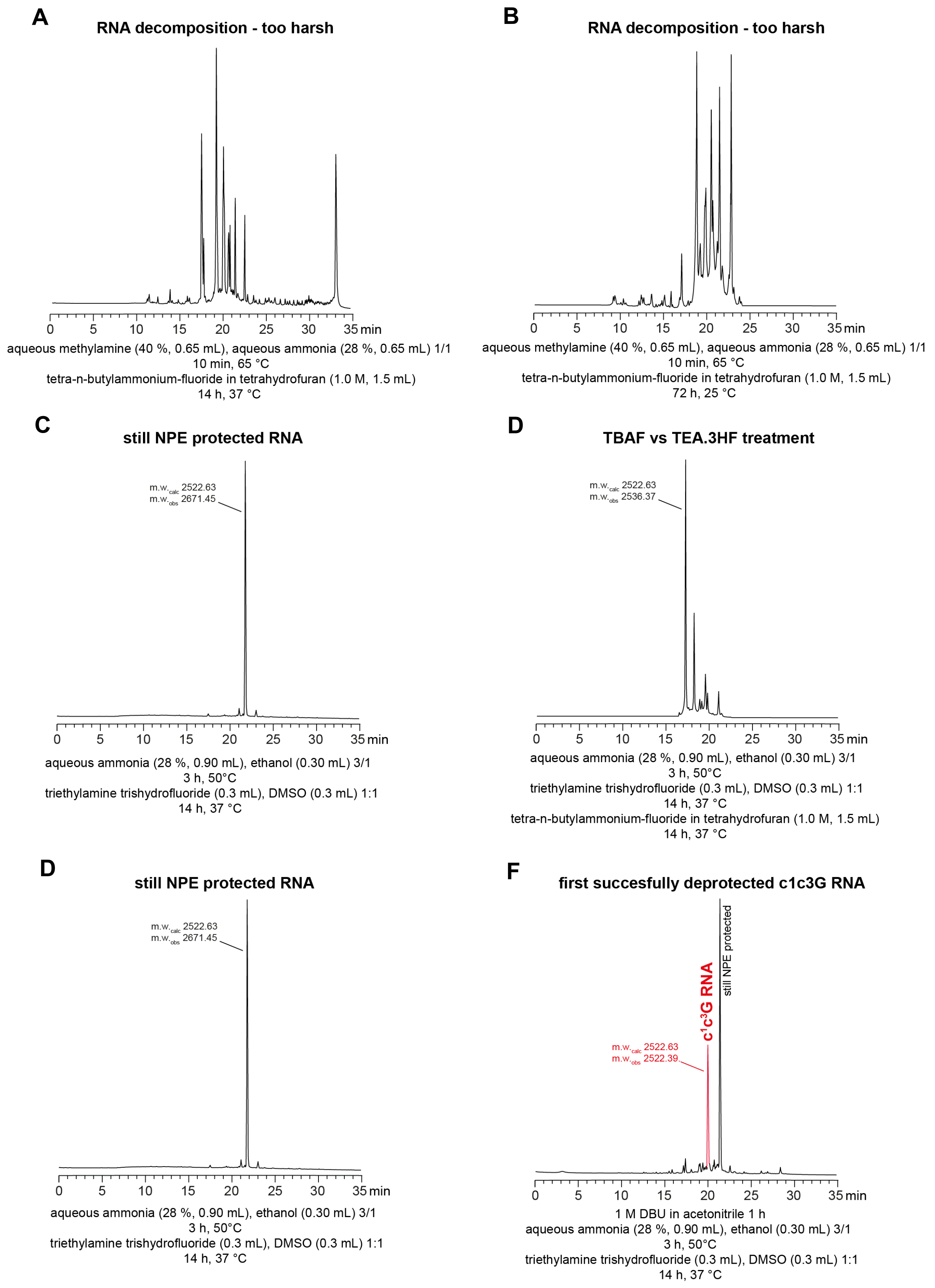

Supporting Figure 1. Deprotection of c^1c^3G containing RNA (synthesized using amidite **12**) – Initial screening of reaction conditions. Crude reaction mixtures of RNA deprotection products (obtained under the deprotection condition as annotated in the individual panels **A** to **F**) were analyzed by anion exchange HPLC. HPLC conditions: Dionex DNAPac column (4x250 mm), 80 °C, 1 ml min^{-1}, 0-60% buffer B in 60 min. Buffer A: Tris-HCl (25 mM), $NaClO_4$ (10 mM), 20% acetonitrile, pH 8.0. Buffer B: Tris-HCl (25 mM), $NaClO_4$ (600 mM), 20% acetonitrile, pH 8.0.

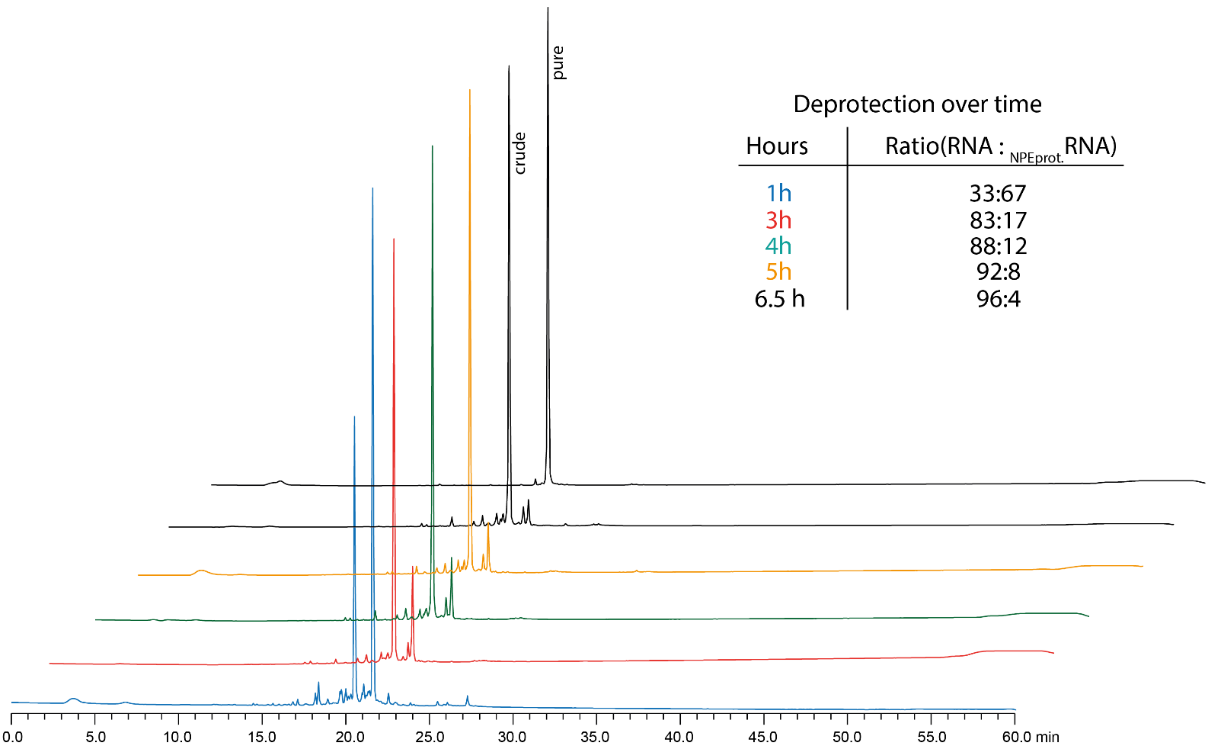

Supporting Figure S2. Deprotection of c[1]c[3]G containing RNA (synthesized using amidite **12**). Reaction time optimization of the first deprotection step: 1 M DBU (1,8-diazabicyclo(5.4.0)undec-7-ene) in acetonitrile at various time points (as indicated); for conditions of the second and third deprotection step see the Supporting Figure S1, panel F). Crude reaction mixtures of RNA deprotection products were analyzed by anion exchange HPLC. HPLC conditions: Dionex DNAPac column (4x250 mm), 80 °C, 1 ml min^{-1}, 0-60% buffer B in 60 min. Buffer A: Tris-HCl (25 mM), $NaClO_4$ (10 mM), 20% acetonitrile, pH 8.0. Buffer B: Tris-HCl (25 mM), $NaClO_4$ (600 mM), 20% acetonitrile, pH 8.0.

A

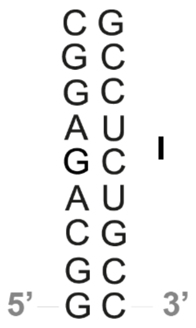

B

concentration / µM	T_m(duplex) / °C
1.6	61.6
5.0	63.7
12.2	66.1
37.7	69.7
98.5	72.6

ΔG^0 kcal mol^{-1}	ΔH^0 kcal mol^{-1}	ΔS^0 cal mol^{-1} K^{-1}
-17.0 ± 0.4	-84.5 ± 1.9	-226 ± 5

C

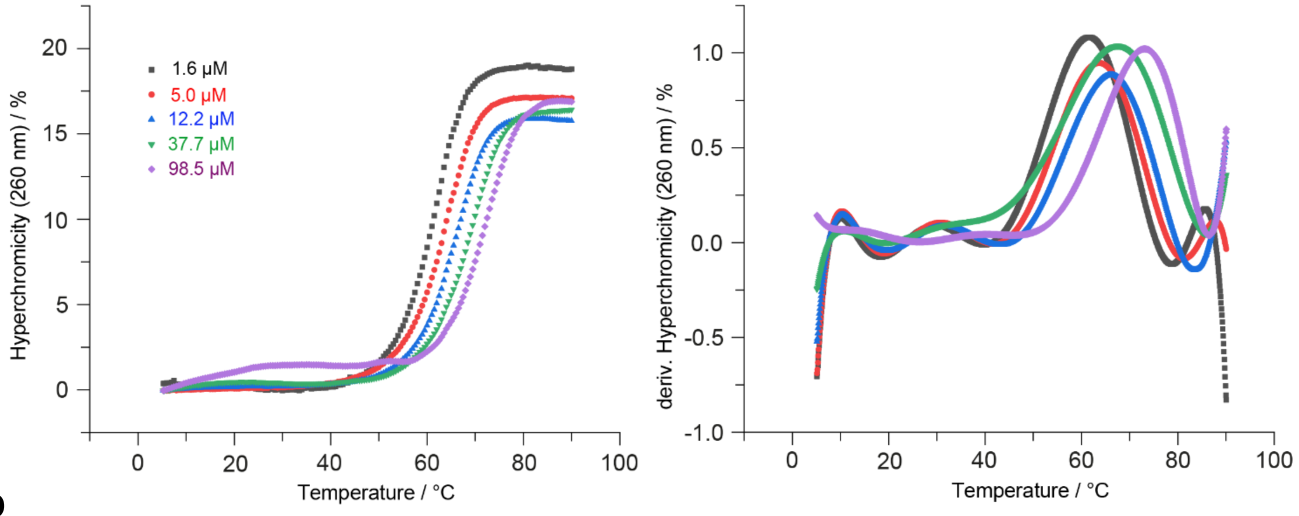

D

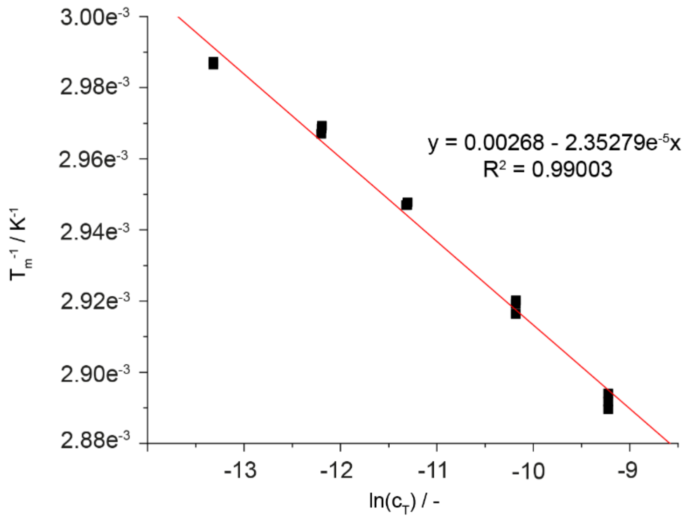

Supporting Figure S3. Thermodynamic analysis of RNA base pairing. **A**) Unmodified 9 bp duplex **I**: Sequence and secondary structure; **B**) Table summarizing RNA concentrations, T_m values, and thermodynamic parameters (van't Hoff analysis); **C**) UV-melting profiles (left) and first derivatives of the melting profiles (right); **D**) Analysis of melting curves by a ln(c) vs 1/T plot (bimolecular concentration dependent melting transitions) assuming a two-state melting process for evaluating the van't Hoff parameters, ΔH_{vH}, ΔS_{vH}, and ΔG_{vH} (summarized in panel B). Conditions: 10 mM Na_2HPO_4, 150 mM NaCl, pH 7.0.

A

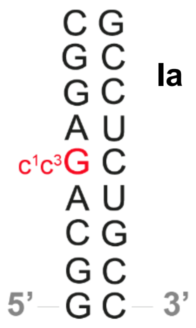

B

concentration / µM	T_m(duplex) / °C
2.2	38.9
5.0	41.5
12.8	44.1
42.0	48.3
83.5	48.8

ΔG^0 kcal mol^{-1}	ΔH^0 kcal mol^{-1}	ΔS^0 cal mol^{-1} K^{-1}
-10.8 ± 0.3	-68.2 ± 3.6	-193 ± 12

C

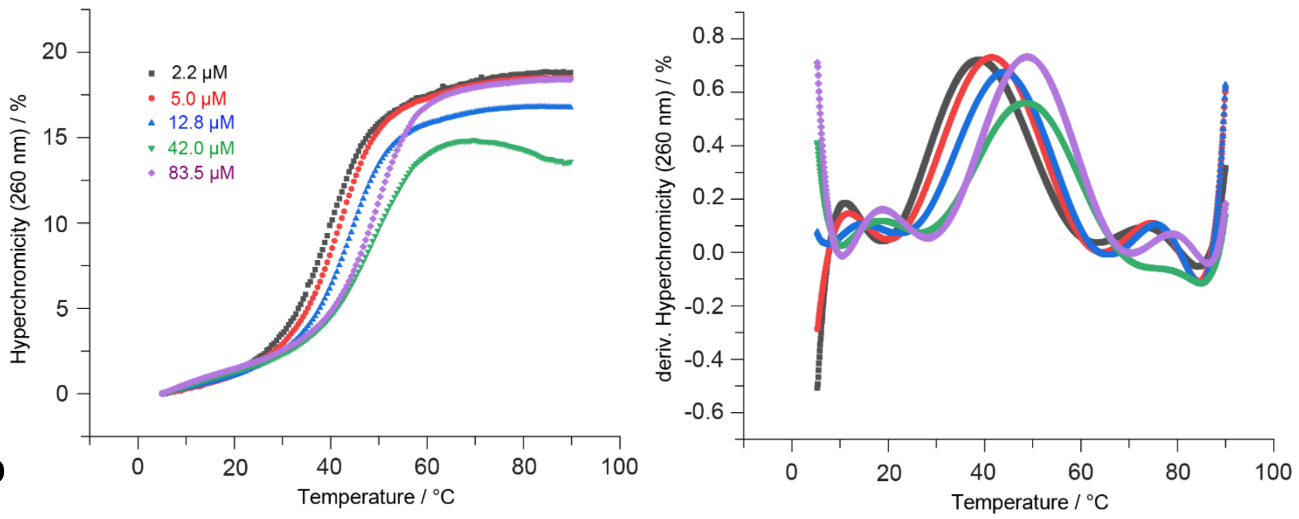

D

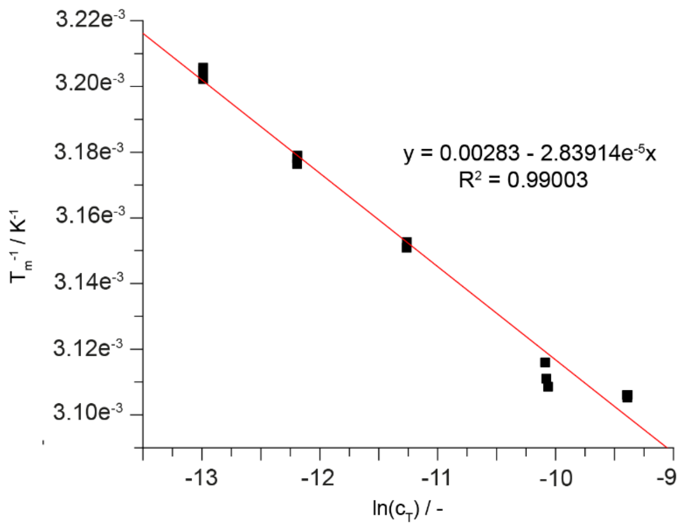

Supporting Figure S4. Thermodynamic analysis of RNA base pairing. **A**) c^1c^3G-modified 9 bp duplex **Ia**: Sequence and secondary structure; **B**) Table summarizing RNA concentrations, T_m values, and thermodynamic parameters (van't Hoff analysis); **C**) UV-melting profiles (left) and first derivatives of the melting profiles (right); **D**) Analysis of melting curves by a ln(c) vs 1/T plot (bimolecular concentration dependent melting transitions) assuming a two-state melting process for evaluating the van't Hoff parameters, ΔH_{vH}, ΔS_{vH}, and ΔG_{vH} (summarized in panel B). Conditions: 10 mM Na_2HPO_4, 150 mM NaCl, pH 7.0.

A

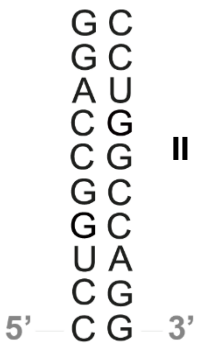

B

concentration / µM	T_m(palindrome) / °C
2.0	69.5
5.9	71.7
16.8	73.8
50.9	76.7
127.7	79.0

ΔG^0 kcal mol^{-1}	ΔH^0 kcal mol^{-1}	ΔS^0 cal mol^{-1} K^{-1}
-21.3 ± 0.1	-104.5 ± 0.6	-279 ± 2

C

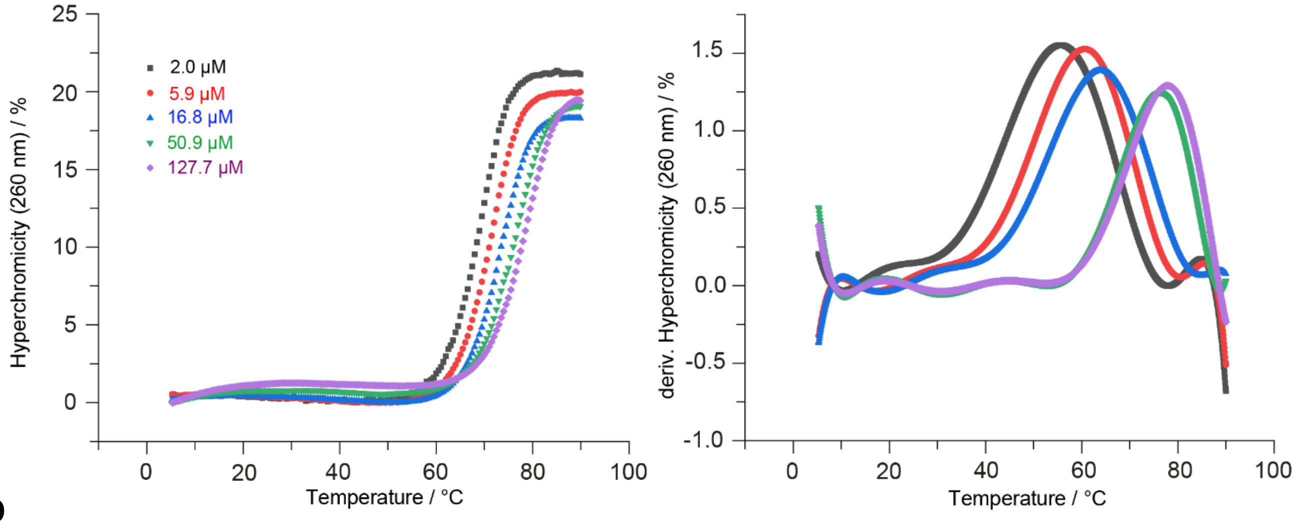

D

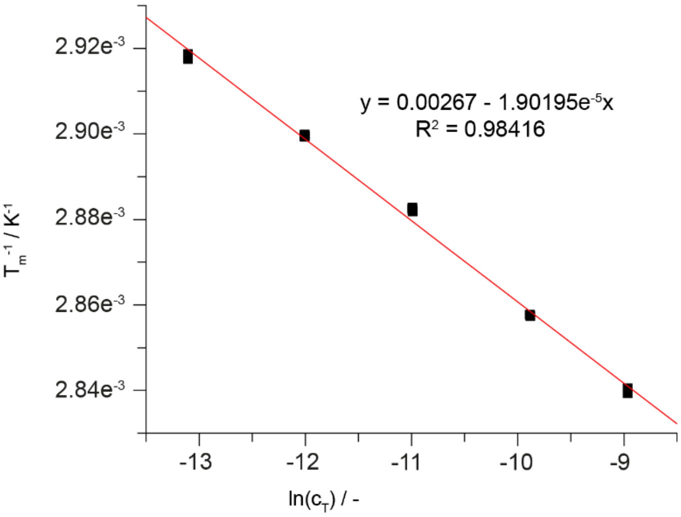

Supporting Figure S5. Thermodynamic analysis of RNA base pairing. **A**) Unmodified 10 bp duplex **II**: Sequence and secondary structure; **B**) Table summarizing RNA concentrations, T_m values, and thermodynamic parameters (van't Hoff analysis); **C**) UV-melting profiles (left) and first derivatives of the melting profiles (right); **D**) Analysis of melting curves by a ln(c) vs 1/T plot (bimolecular concentration dependent melting transitions) assuming a two-state melting process for evaluating the van't Hoff parameters, ΔH_{vH}, ΔS_{vH}, and ΔG_{vH} (summarized in panel B). Conditions: 10 mM Na_2HPO_4, 150 mM NaCl, pH 7.0.

A

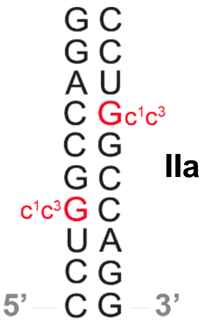

B

concentration / µM	T_m(palindrome) / °C
2.2	32.1
4.7	33.6
13.5	35.6
41.1	39.3
99.5	41.7

ΔG^0 kcal mol^{-1}	ΔH^0 kcal mol^{-1}	ΔS^0 cal mol^{-1} K^{-1}
-9.4 ± 0.3	-74.1 ± 2.5	-217 ± 7

C

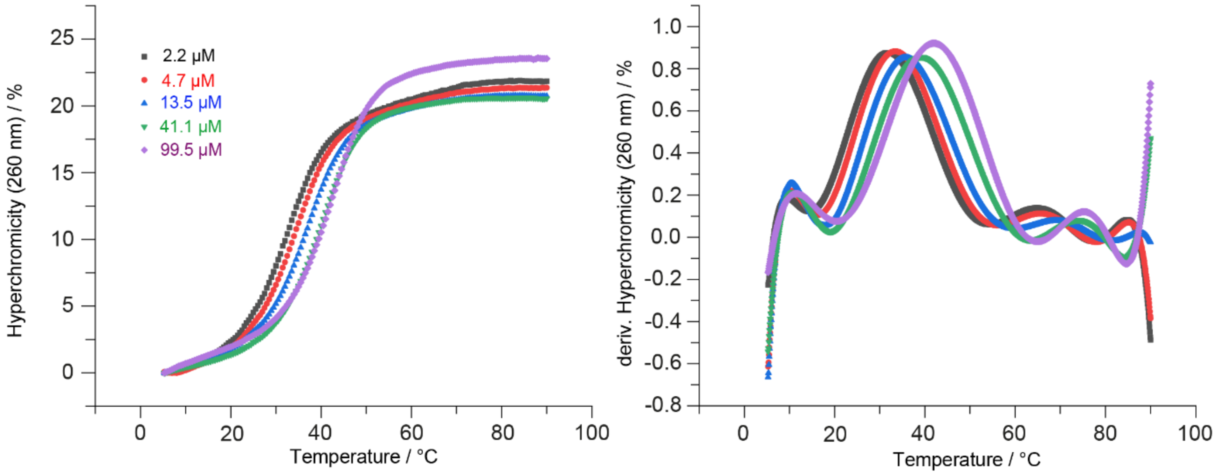

D

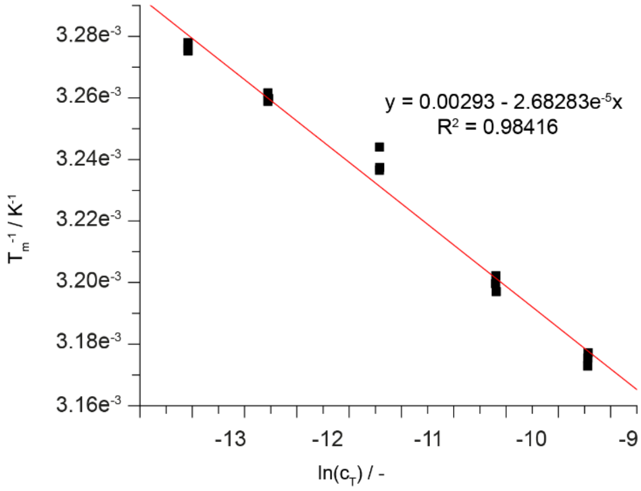

Supporting Figure S6. Thermodynamic analysis of RNA base pairing. **A**) c^1c^3G-modified 10 bp duplex **IIa**: Sequence and secondary structure; **B**) Table summarizing RNA concentrations, T_m values, and thermodynamic parameters (van't Hoff analysis); **C**) UV-melting profiles (left) and first derivatives of the melting profiles (right); **D**) Analysis of melting curves by a ln(c) vs 1/T plot (bimolecular concentration dependent melting transitions) assuming a two-state melting process for evaluating the van't Hoff parameters, ΔH_{vH}, ΔS_{vH}, and ΔG_{vH} (summarized in panel B). Conditions: 10 mM Na_2HPO_4, 150 mM NaCl, pH 7.0.

A

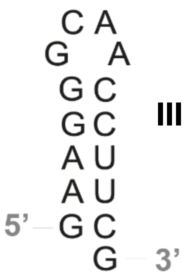

B

concentration / µM	T_m(hairpin) / °C
4.0	70.8
12.4	71.0

ΔG^0 kcal mol^{-1}	ΔH^0 kcal mol^{-1}	ΔS^0 cal mol^{-1} K^{-1}
-6.9 ± 0.5	-54.1 ± 3.5	-159 ± 10

C

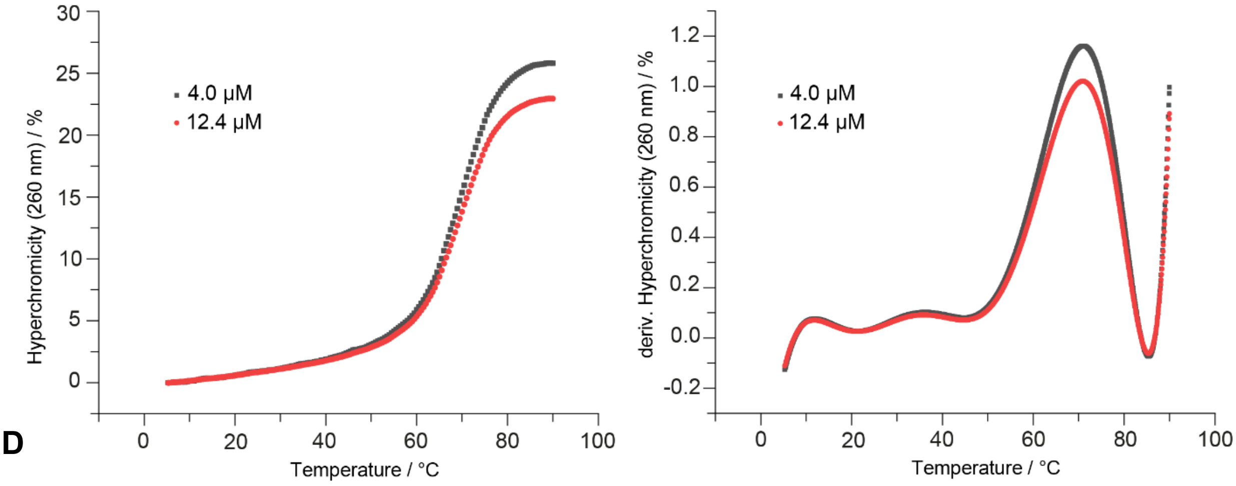

D

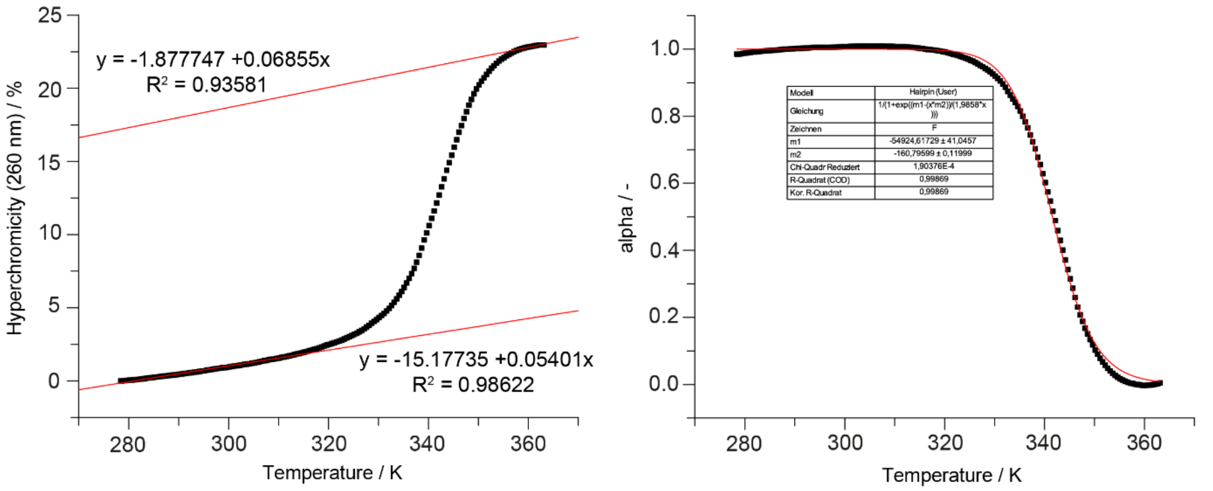

Supporting Figure S7. Thermodynamic analysis of RNA base pairing. **A**) Unmodified 15 nt hairpin **III**: Sequence and secondary structure; **B**) Table summarizing RNA concentrations, T_m values, and thermodynamic parameters (van't Hoff analysis); **C**) UV-melting profiles (left) and first derivatives of the melting profiles (right); **D** Graphical analysis of melting curves (unimolecular concentration independent melting transition) assuming a two-state melting process for evaluating the van't Hoff parameters, ΔH_{vH}, ΔS_{vH}, and ΔG_{vH} (summarized in panel B). Conditions: 10 mM Na_2HPO_4, 150 mM NaCl, pH 7.0.

A

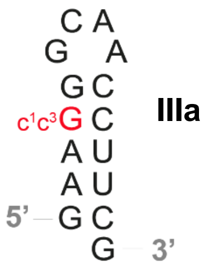

B

concentration / µM	T_m(hairpin) / °C
3.8	36.7
10.8	36.3

ΔG^0 kcal mol^{-1}	ΔH^0 kcal mol^{-1}	ΔS^0 cal mol^{-1} K^{-1}
-1.4 ± 0.1	-32.4 ± 4.7	-105 ± 15

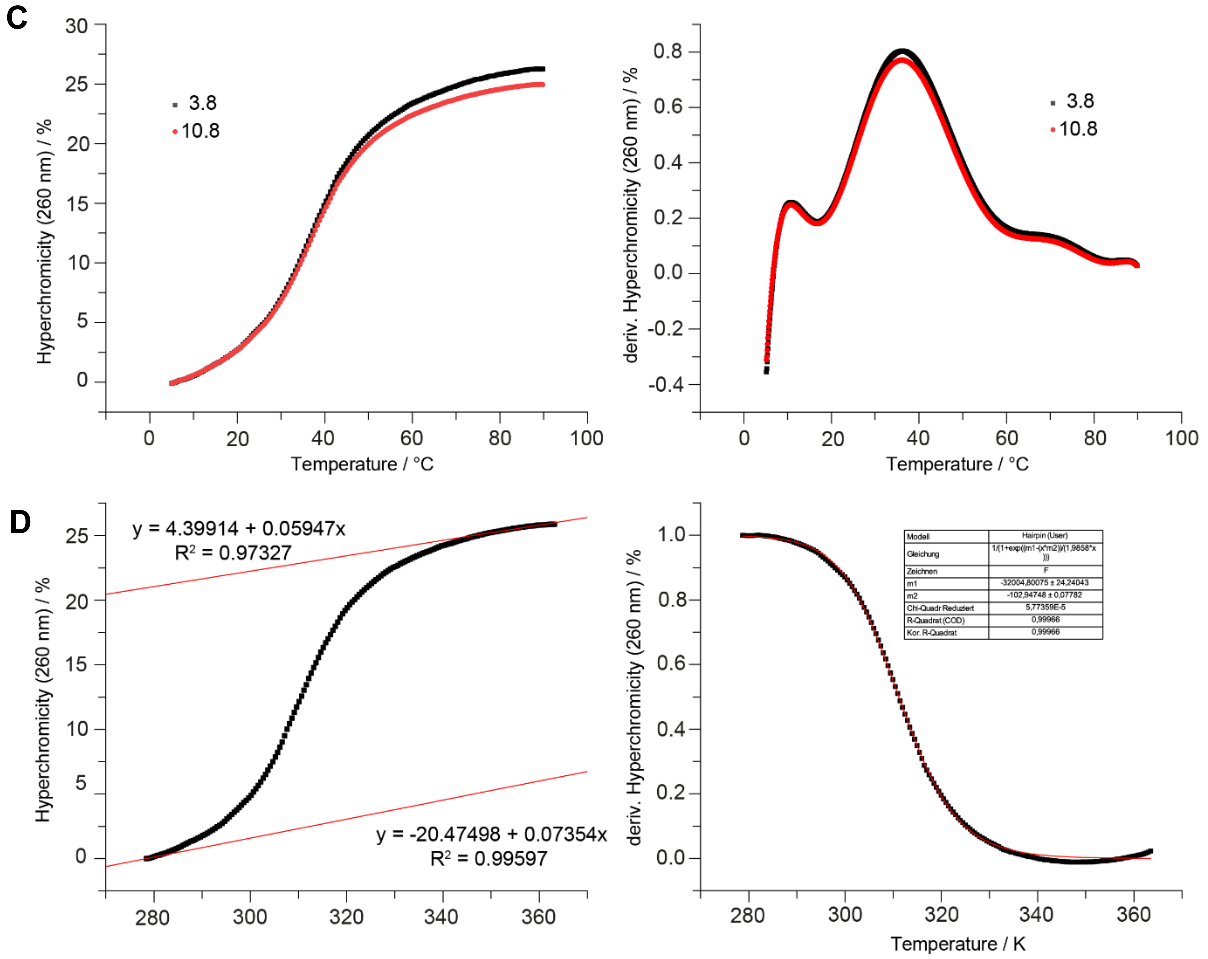

Supporting Figure S8. Thermodynamic analysis of RNA base pairing. **A**) c[1]c[3]G-modified 15 nt hairpin **IIIa**: Sequence and secondary structure; **B**) Table summarizing RNA concentrations, T_m values, and thermodynamic parameters (van't Hoff analysis); **C**) UV-melting profiles (left) and first derivatives of the melting profiles (right); **D**) Graphical analysis of melting curves (unimolecular concentration independent melting transition) assuming a two-state melting process for evaluating the van't Hoff parameters, ΔH_{vH}, ΔS_{vH}, and ΔG_{vH} (summarized in panel B). Conditions: 10 mM Na_2HPO_4, 150 mM NaCl, pH 7.0.

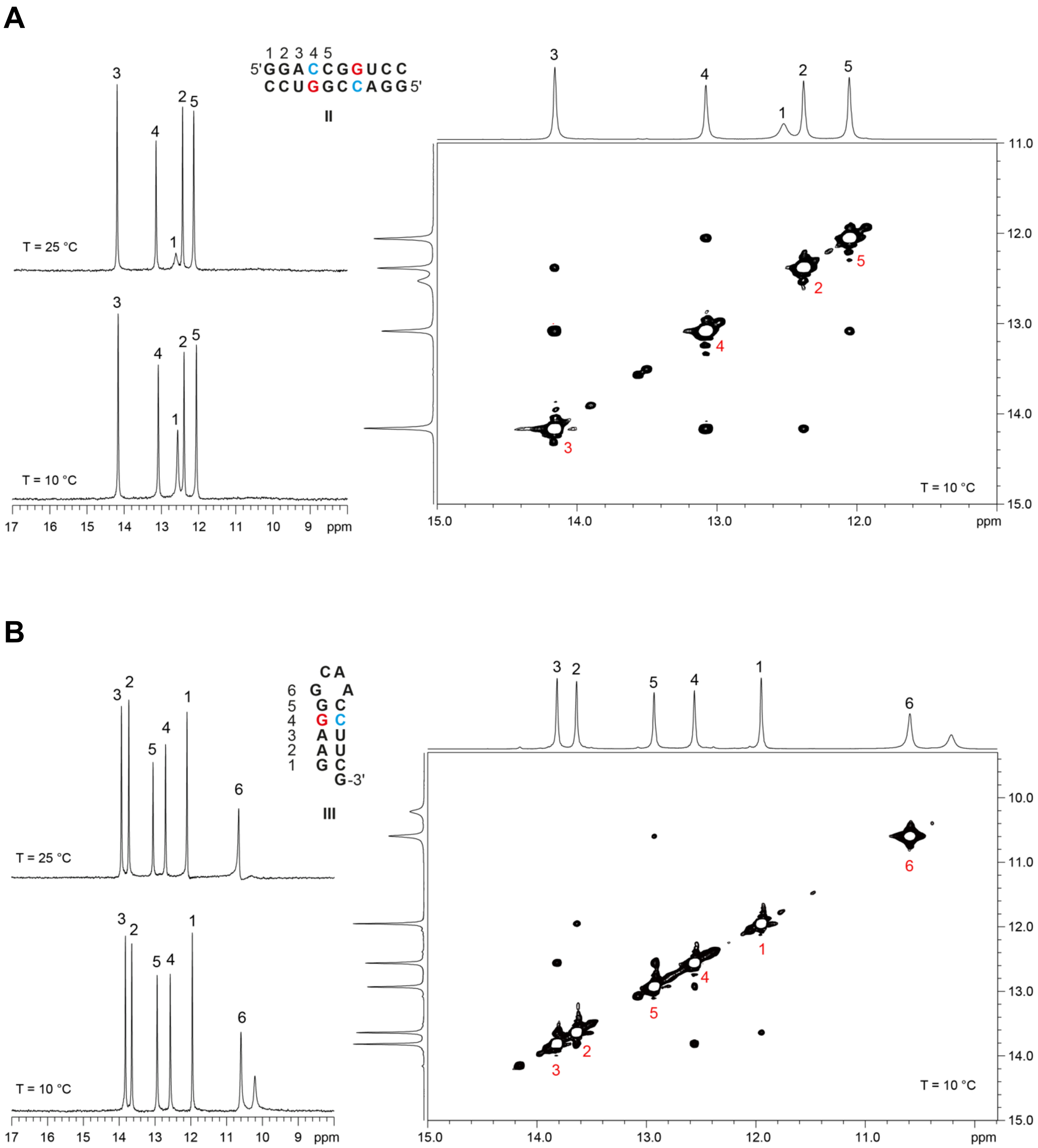

Supporting Figure S9. ^{1}H-NMR spectroscopic analysis. **A**) 10 nt RNA palindrome **II** and **B**) 15 nt RNA hairpin **III**. Sequence, secondary structure, and temperature-dependent spectra of the imino proton pm region (left);^{1}H, ^{1}H-NOESY NMR spectrum used for peak assignment (right). Conditions: c(RNA): 0.4 mM, 15 mM sodium phosphate, 25 mM NaCl, 10 % D_2O, pH 6.5; temperature as indicated.

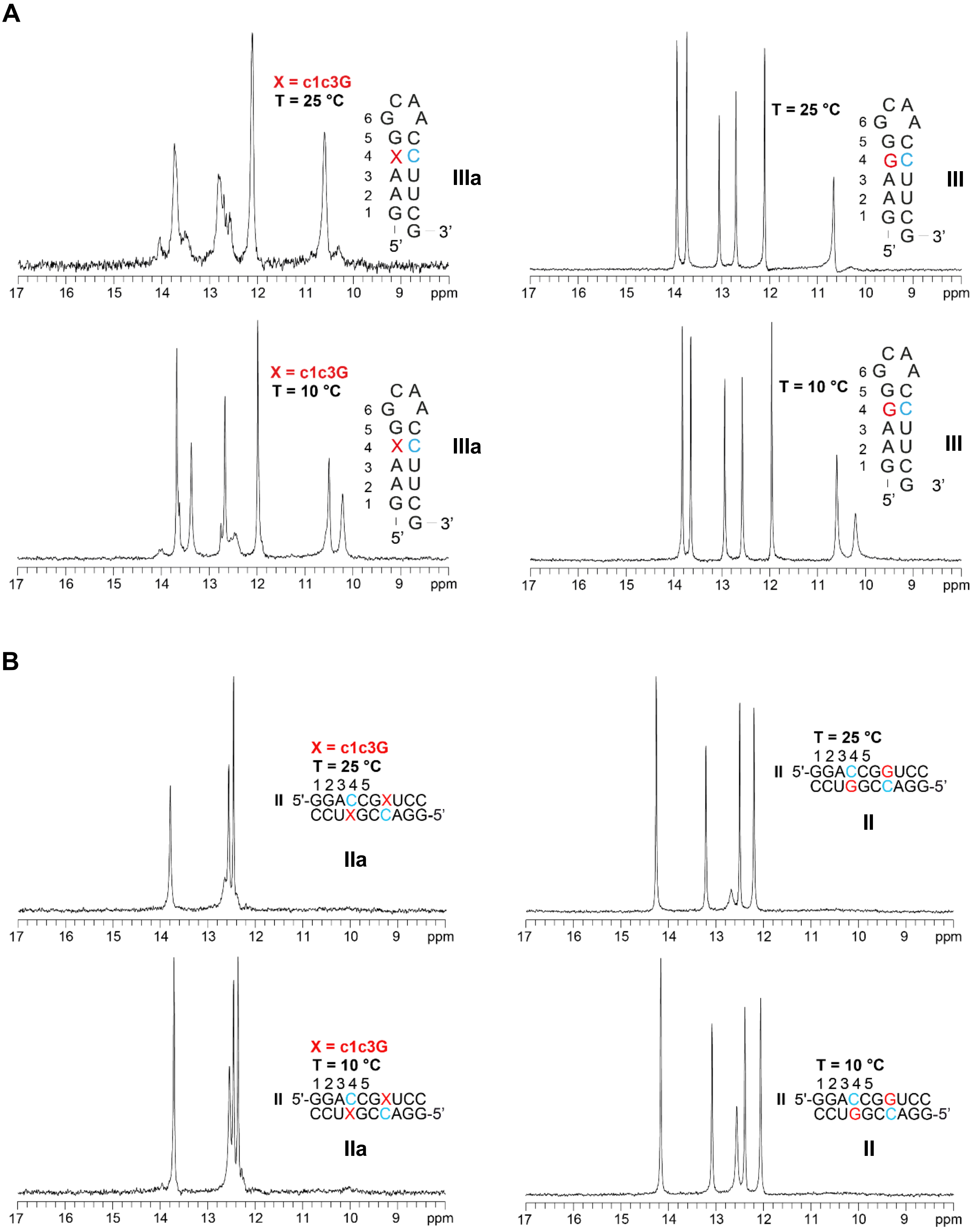

Supporting Figure S10. Comparative ^{1}H-NMR imino proton spectroscopy. **A**) Imino proton spectra of $c^{1}c^{3}G$ modified hairpin **IIIa** (left) and unmodified reference **III** (right) at two different temperatures 25 °C (top) and 10 °C (bottom). **B)** Imino proton spectra of $c^{1}c^{3}G$ modified duplex **IIa** (left) and unmodified reference **II** (right) at two different temperatures 25 °C (top) and 10 °C (bottom). Conditions: c(RNA): 0.2 mM, 15 mM sodium phosphate, 25 mM NaCl, 10 % D_2O, pH 6.5.

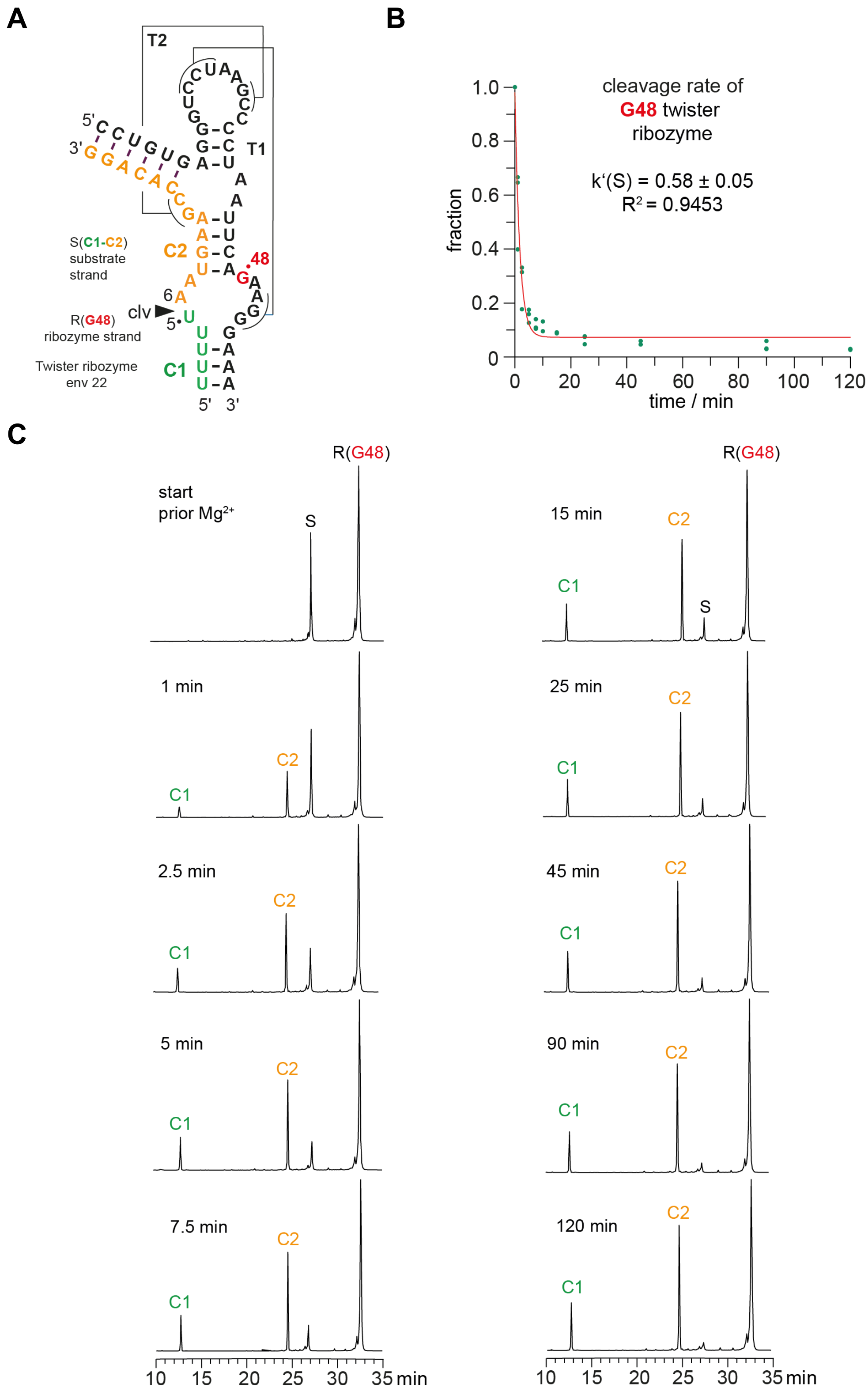

Supporting Figure S11. Self-cleavage of the *env22* twister ribozyme. **A**) Sequence and secondary structure; **B**) Estimation of observed rate from fractions (S) obtained by HPLC analysis **C**) Anion exchange HPLC traces of the reaction time course; reaction conditions: c(RNA) = 55 µM each RNA strand (1:1 ratio); 2 mM $MgCl_2$, 100 mM KCl, 30 mM HEPES, pH 7.5, 23 °C. The reaction was stopped at the indicated time points by drawing a 2 µL sample and mixing it with 4 µL of 40 mM Na_2EDTA, followed by dilution to 100 µL of water. HPLC conditions: Dionex DNAPac column (4x250 mm), 80 °C, 1 ml min^{-1}, 0-60% buffer B in 60 min. Buffer A: Tris-HCl (25 mM), $NaClO_4$ (10 mM), 20% acetonitrile, pH 8.0. Buffer B: Tris-HCl (25 mM), $NaClO_4$ (600 mM), 20% acetonitrile, pH 8.0.

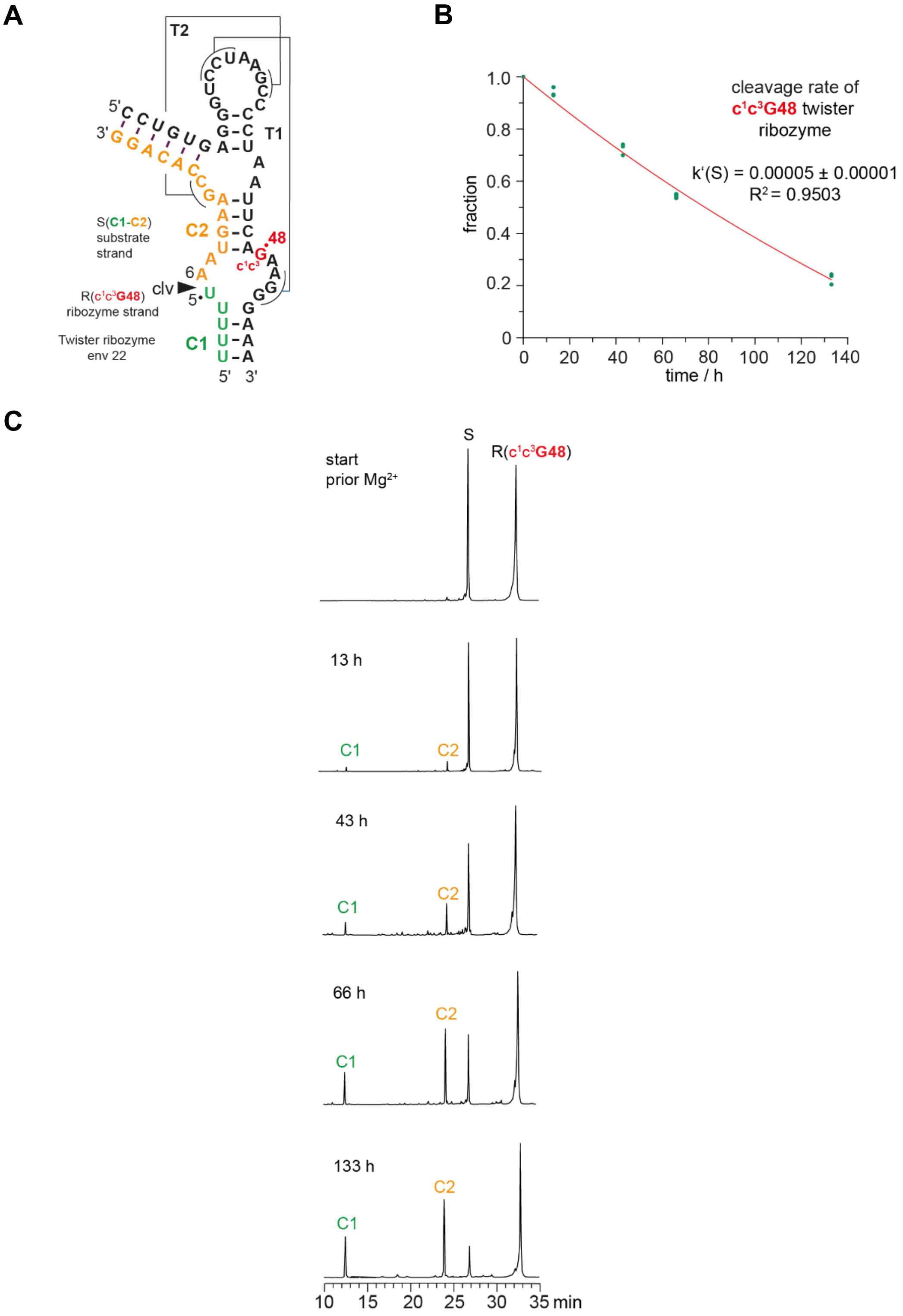

Supporting Figure S12. Self-cleavage of the position 48 c[1]c[3]G modified *env22* twister ribozyme. **A**) Sequence and secondary structure; **B**) Estimation of observed rate from fractions (S) obtained by HPLC analysis **C**) Anion exchange HPLC traces of the reaction time course; reaction conditions: c(RNA) = 55 µM each RNA strand (1:1 ratio); 2 mM $MgCl_2$, 100 mM KCl, 30 mM HEPES, pH 7.5, 23 °C. The reaction was stopped at the indicated time points by drawing a 2 µL sample and mixing it with 4 µL of 40 mM Na_2EDTA, followed by dilution to 100 µL of water. HPLC conditions: Dionex DNAPac column (4x250 mm), 80 °C, 1 ml min^{-1}, 0-60% buffer B in 60 min. Buffer A: Tris-HCl (25 mM), $NaClO_4$ (10 mM), 20% acetonitrile, pH 8.0. Buffer B: Tris-HCl (25 mM), $NaClO_4$ (600 mM), 20% acetonitrile, pH 8.0.
